# *SCARF2* is a target for chronic obstructive pulmonary disease: Evidence from multi‐omics research and cohort validation

**DOI:** 10.1111/acel.14266

**Published:** 2024-07-03

**Authors:** Sai Wang, Yuanyi Yue, Xueqing Wang, Yue Tan, Qiang Zhang

**Affiliations:** ^1^ Department of Otorhinolaryngology The First Hospital of China Medical University Shenyang China; ^2^ Department of Gastroenterology Shengjing Hospital of China Medical University Shenyang China; ^3^ Department of Pulmonary and Critical Care Medicine Shengjing Hospital of China Medical University Shenyang China

**Keywords:** chronic obstructive pulmonary disease, idiopathic pulmonary fibrosis, leukocyte telomere length, Mendelian randomization, *SCARF2*

## Abstract

Age‐related chronic inflammatory lung diseases impose a threat on public health, including idiopathic pulmonary fibrosis (IPF) and chronic obstructive pulmonary disease (COPD). However, their etiology and potential targets have not been clarified. We performed genome‐wide meta‐analysis for IPF with the largest sample size (2883 cases and 741,929 controls) and leveraged the summary statistics of COPD (17,547 cases and 617,598 controls). Transcriptome‐wide and proteome‐wide Mendelian randomization (MR) designs, together with genetic colocalization, were implemented to find robust targets. The mediation effect was assessed using leukocyte telomere length (LTL). The single‐cell transcriptome analysis was performed to link targets with cell types. Individual‐level data from UK Biobank (UKB) were used to validate our findings. Sixteen genetically predicted plasma proteins were causally associated with the risk of IPF and 6 proteins were causally associated with COPD. Therein, genetically‐elevated plasma level of *SCARF2* protein should reduce the risk of both IPF (odds ratio, OR = 0.9974 [0.9970, 0.9978]) and COPD (OR = 0.7431 [0.6253, 0.8831]) and such effects were not mediated by LTL. Genetic colocalization further corroborated these MR results of SCARF2. The transcriptome‐wide MR confirmed that higher expression level of *SCARF2* was associated with a reduced risk of both. However, the single‐cell RNA analysis indicated that *SCARF2* expression level was only relatively lower in epithelial cells of COPD lung tissue compared to normal lung tissue. UKB data implicated an inverse association of serum *SCARF2* protein with COPD (hazard ratio, HR = 1.215 [1.106, 1.335]). The *SCARF2* gene should be a novel target for COP.

## Graphical abstract

This study integrated genome, transcriptome, and proteome data to explore the potential target for chronic obstructive pulmonary disease (COPD) and idiopathic pulmonary fibrosis (IPF), and found the robust associations of *SCARF2*with them. Furthermore, single cell transcriptome analysis implicated that *SCARF2*was lowly expressed in epithelial cells for COPD lung tissues while not for IPF tissues. Such results were validated using protein data from UK biobank, strengthening the role of *SCARF2* in COPD.

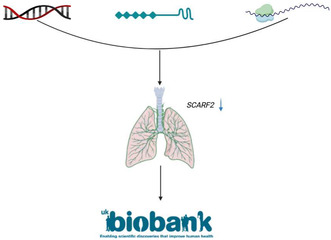

**Abbreviations**
APOA5: apolipoprotein A5
CAD: coronary artery disease
CHD: coronary heart disease
COPD: obstructive pulmonary disease
CVD: cardiovascular diseases
eQTL: expression quantitative trait locus
FDR: false discovery rate
FLRT3: fibronectin leucine rich transmembrane protein 3
FN1: fibronectin 1
GTEx: genotype‐tissue expression
GWAS: genome‐wide association study
HR: hazard ratio
IPF: idiopathic pulmonary fibrosis
IVW: inverse‐variance weighted
LD: linkage disequilibrium
LTL: leukocyte telomere length
MAF: minor allele frequency
MNP: mononuclear phagocytes
MR: Mendelian randomization
OR: odds ratio
PC: principal component
PCSK9: proprotein convertase subtilisin/kexin type 9
PPH3: posterior probability of H3
PPH4: posterior probability of H4
pQTL: protein quantitative trait locus
RCT: randomized controlled trial
SCARF2: scavenger receptor class F, member 2
SE: standard error
SMR: Summary‐data‐based Mendelian Randomization
SNP: single nucleotide polymorphism
UKB: UK Biobank
UMAP: uniform manifold approximation and projection

## 1 INTRODUCTION

Ageing‐related chronic inflamatory lung diseases impose heavy economic and social burden on public health, including idiopathic pulmonary fibrosis (IPF) and chronic obstructive pulmonary disease (COPD). IPF, characterized by interstitial fibrosis without definite cause, occurs primarily in aged adults and is usually accompanied by a poor prognosis, with a median survival of 3.8 years (Lederer & Martinez, 2018). COPD is usually diagnosed when it is pathologically advance and clinically irreversible, and the age at initial diagnosis is often over 60 years old (Christenson et al., 2022). Similar characteristics were shared by COPD and IPF, such as older age, environmental pollutants, remodeling of lung tissue, reduced lung function and genetic risk factors, and their incidences are expected to rise annually (Singla et al., 2023). Although the causes of COPD are obvious and those of IPF are unknown, the therapeutics for them are still limited currently and the potential mechanism is not well understood.

It has been reported that cellular senescence plays a key role in chronic lung disease and can be treated as candidate therapeutic targets (Barnes et al., 2019). Furthermore, a recent Mendelian randomization (MR) study implicated that leukocyte telomere length (LTL), an important biomarker associated with ageing, is causally associated with IPF while not with COPD, suggesting the distinct mechanism behind these two diseases (Duckworth et al., 2021). Such results are thought‐provoking as it suggested that these two diseases might be affected differently by the same biomarker mechanistically. Notably, MR is a method for casual inference and has been recommended to provide clinical evidence only inferior to a randomized controlled trial (RCT) (Davies et al., 2018), and the recently developed proteome‐wide MR design, which uses genetic variants (usually single nucleotide polymorphism, SNP) associated with serum protein level as the instruments (also called as protein quantitative trait locus, pQTL), can even facilitate the therapeutic target discovery and drug development, for instance, Gaziano et al. reported actionable druggable targets for COVID‐19 like *IFNAR2* and *ACE2 (*Gaziano et al., 2021) and another proteome‐wide MR study implicated candidate targets for heart failure as well (Henry et al., 2022). However, there is no proteome‐wide MR design to explain the divergent mechanisms between IPF and COPD. What's more, it is little known about how LTL can affect COPD and IPF differently in a molecular level.

Due to the rapid development in genome‐wide association studies (GWAS), the proteome‐wide GWAS summary statistics are accessible, together with GWAS summary statistics for LTL, IPF, and COPD. Thus, we aimed to leverage the transcriptome, proteome, LTL, IPF and COPD GWAS data to explore the druggable targets for these two diseases and explain how telomere‐associated proteins affect them differently, hoping to elucidate the divergent mechanisms behind them.

## 2 MATERIALS AND METHODS

### 2.1 Genome‐wide summary statistics for gene expression, serum proteins, LTL, IPF, and COPD

Genetic associations with gene expression were obtained from two databases: one was from the eQTLGen database (https://www.eqtlgen.org/) (Võsa et al., 2021) and the other was from the GTEx (genotype‐tissue expression) database (https://www.gtexportal.org/home/index.html) (Urbut et al., 2019). Therein, only cis‐eQTLs (expression quantitative trait locus) were used as genetic instruments if the false discovery rate (FDR) was <0.05. The eQTLs from the eQTLGen database were measured in blood and those from the GTEx database were measured in blood as well.

Genetic associations with serum proteins were retrieved from two sources: one was from the publication by Zheng et al. (2020) and the other was from a proteome‐wide GWAS by Ferkingstad et al. (2021). Therein, the former study consisted of 1064 reliable pQTLs of 955 proteins from 5 independent proteome‐wide GWAS and the maximum sample size was 6861 (Yao et al., 2018; Zheng et al., 2020), and the latter included 10,727 reliable pQTLs of 2611 proteins from an independent proteome‐wide GWAS with 35,559 Icelanders (Ferkingstad et al., 2021).

The GWAS of LTL was performed using 472,174 well‐characterized UK Biobank European participants, adjusting for age, sex, SNP array chip and the first 10 principal components (PCs) (Codd et al., 2021). Genetic associations with IPF were obtained from two studies: one is a GWAS performed using 1369 cases and 435,866 controls, adjusting for baseline age, sex, UK Biobank, assessment center, and genetic PCs (Duckworth et al., 2021); the other GWAS was performed in the FinnGen population consisting of 1514 cases and 306,063 controls (Kurki et al., 2023). The GWAS summary statistics of COPD were retrieved from 17,547 cases and 617,598 controls, adjusting for age, age^2^, sex, age × sex, age^2^ × sex and the top 20 genetic PCs (Sakaue et al., 2021). The diagnostic criteria for IPF and COPD can be found in each original GWAS article. All GWAS summary statistics are publicly available and can be freely downloaded without attempting to unveil individual‐level information.

### 2.2 Genome‐wide meta‐analysis of IPF summary statistics

To maximize the power of this study, we meta‐analyzed two IPF GWAS summary statistics using METAL software with the fixed‐effects model (https://genome.sph.umich.edu/wiki/METAL) (Willer et al., 2010). This meta‐analysis enrolled a total of 2883 IPF cases and 741,929 controls. Before meta‐analysis, we conducted quality control for each GWAS separately where SNPs with low minor allele frequency (MAF <0.01) were removed and SNPs with low imputation quality (INFO score <0.9) were filtered out. The duplicated SNPs were removed as well. The variants' “rsid” were imputed using the dbSNP 144 data. We used the standard error (SE) as the weight in the model and applied genomic control to the results.

### 2.3 Summary‐level Mendelian randomization design

Traditional MR design should be conducted abide by three core assumptions: (1) genetic instruments are closely associated the exposure; (2) there are no common confounders between genetic instruments and the outcome; (3) genetic variants are not associated with the outcome except via the way of exposure (Emdin et al., 2017). If the exposure is a molecular quantitative trait, the genetic instruments should be located in or near the gene that encodes the drug target (Walker et al., 2017). In this study, we treated the pQTLs as genetic instruments, protein levels as exposures, and LTL, IPF, and COPD as outcomes. The genetic instruments were selected using the criteria: MAF >0.01, INFO >0.9 and associated with <5 proteins. Then, the instruments were pruned using the linkage disequilibrium (LD) threshold *r* ^2^ <0.001. A SNP proxy (*r* ^2^ > = 0.9) would be used if the genetic association was missing in the outcome dataset. In the discovery stage, we used pQTLs from the Iceland population as instruments to estimate the causal effects of serum protein levels on LTL, IPF, and COPD separately due to a relatively large number of proteins and study participants. In the validation stage, we used pQTLs from Zheng et al. to assess the causal effects separately again. Only cis‐pQTLs were included in the analysis as they offered the direct biological insights. The MR estimate was considered robustly significant if it passed the FDR control in the discovery stage and indicated the same effect direction as in the validation set with a suggestive significance (*p* < 0.05).

### 2.4 Assessing associations of gene expression with COPD and IPF

For these significant causal proteins, we assessed the causal associations of corresponding mRNA levels with IPF and COPD to explain the potential mechanisms using eQTLs. The summary statistics of eQTLGen were used in the discovery stage and those of GTEx were analyzed in the validation stage. Only targets displaying significant associations with these two diseases (*p* < 0.05) and concordant directions with proteins were deemed to be qualified for explaining potential mechanisms. According to those validated targets, we could explain that the protein levels may be directly impacted by the gene expression.

### 2.5 Colocalization analysis of cis‐pQTL with two diseases

Genetic colocalization was performed to detect the shared causal variant by two traits and was established to test 5 hypotheses using “coloc” package (https://github.com/chr1swallace/coloc): (1) H_0_: the variant is not associated with either trait; (2) H_1_: the variant is associated with only trait 1; (3) H_2_: the variant is associated with only trait 2; (4) H_3_: the variant is associated with both traits (two independent mechanisms); (5) H_4_: the variant is associated with both traits (one shared SNP) (Giambartolomei et al., 2014). A higher posterior probability of H_3_ (PPH_3_ >80%) indicated that the significant MR estimate might rise from LD and a higher posterior probability of H_4_ (PPH_4_ >80%) would give support to the significant MR result. We adopted the default priors as p1 = 1 × 10^−4^, p2 = 1 × 10^−4^ and p12 = 1 × 10^−5^. The priors were changed to 1 = 1 × 10^−5^, p2 = 1 × 10^−5^ and p12 = 1 × 10^−6^ to test the robustness of colocalization.

### 2.6 Mediation analysis with LTL as the mediator

Considering that LTL is a biomarker of aging and according to the study by Duckworth et al., shorter LTL can lead to IPF instead of COPD (Duckworth et al., 2021). Such results indicated that LTL should mediate the pathogenesis of IPF and COPD differently via distinct proteins and pathways. Thus, we implemented the mediation analysis to answer whether LTL is a mediator that affects IPF and COPD differently. This mediation analysis is implemented in the context of MR design which requires full summary statistics of both exposures and outcomes (Carter et al., 2021). The indirect effect of plasma protein on the outcome is calculated by multiplying the direction effect of plasma protein on LTL and the direct effect of LTL on IPF/COPD after adjusting for the protein level (Carter et al., 2021). We downloaded the full summary statistics of potential proteins which should be causally associated with LTL and IPF/COPD, and used the MR design to answer whether these proteins could affect IPF/COPD via the way of LTL.

### 2.7 Phenome‐wide association lookup, enrichment analysis, and druggable target search

To assess the genetic effects of our identified targets on whole human phenotypes, we looked up for all potential phenotypes that were associated with SNPs located in the target gene in the open GWAS database (*p* < 5 × 10^−8^). The open GWAS database consisted of 245,615,011,379 genetic associations from 42,346 GWAS summary datasets till the end of 2023 and can be queried for scientific research without restriction (https://gwas.mrcieu.ac.uk/). The significant proteins were enriched for possible pathway in the Reactome database and for possible diseases in the DisGeNET using the WebGestalt tool (http://www.webgestalt.org/) (Liao et al., 2019). The potential druggable targets were evaluated in the DrugBank (https://go.drugbank.com/).

### 2.8 Single‐cell transcriptome analysis of significant targets associated with IPF and COPD

We tried to determine whether the significant targets discovered by MR analyses and colocalization were expressed in specific cell types in lung tissues using single‐cell transcriptome data. Two datasets were retrieved from single cell database (ID SCP2155) (Fabre et al., 2023). Gene expression matrices were processed by Seurat (4.3.0.1), and cells were filtered using cutoffs of a minimum of 300 genes per cell and a maximum of 20% mitochondrial reads. Dimensionality reduction, cell clustering, and differential analysis between different disease ontologies were processed by Seurat (4.3.0.1). Harmonization was used to correct for patient batch effects and the top 40 PCs were used for RunUAP and FindNeighbors. For cluster selection, the resolution value was set as 0.6.

### 2.9 Validation of discovered proteins using individual‐levels from UK biobank

To strengthen the findings from abovementioned analyses, we obtained the individual‐level data of serum proteins and clinical information from UK Biobank via the required application. The COPD cases were determined using the ICD‐10 (J41, J42, J43, and J44) and the IPF cases were defined by ICD‐10J84.1, J84.0, J84.8, and J84.9. The proteins were quantified using the Olink panels and we estimated the associations of Normalized Protein eXpression (NPX values) with COPD and IPF using the Cox proportional hazard model with adjustment of age, sex, BMI and socioeconomic status. The detailed information of clinical information and proteins can be found in the website: https://www.ukbiobank.ac.uk/. The UK Biobank received ethical approval from the North‐West Multi‐Centre Research Ethics Committee (11/NW/0382). All participants provided written informed consent and got informed before taking part in.

### 2.10 Statistical analysis and data visualization

Preliminarily, we calculated the *F* statistics for each pQTL using the formula (*F* = beta^2^/SE^2^, beta is the effect size representing the genetic association with exposure) and used MR Steiger test to remove pQTLs that explained more variance of outcome (Hemani et al., 2017). For single pQTL, we used Wald ratio method to estimate the causal effect and then combined multiple effect sizes using the inverse‐variance weighted (IVW) method with a random‐effects model. For multiple pQTLs, the weighted‐median method was adopted as a supplement. The eQTLs were analyzed using the same method called “SMR (Summary‐data‐based Mendelian Randomization)” (Zhu et al., 2016). In the mediation analysis, the indirect effect size was calculated using two methods called “Product of coefficients method” and “Difference in coefficients method” suggested by Carter et al., and the 95% confidence interval was calculated using the bootstrap method (Carter et al., 2021). The heterogeneity was assessed by Cochran's Q value and horizontal pleiotropy was assessed by MR‐PRESSO methods (Verbanck et al., 2018). The FDR was calculated to control inflated false positive rate.

**FIGURE 1:**
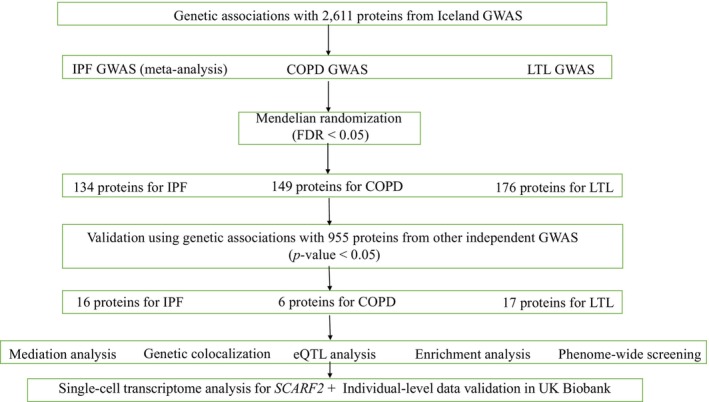
The main flowchart of this study. COPD, chronic obstructive pulmonary disease; FDR, false discovery rate; GWAS, genome‐wide association study; IPF, idiopathic pulmonary fibrosis; LTL, leukocyte telomere length. The “meta‐analysis” indicates that a GWAS meta‐analysis was performed for IPF in this study. The “PP of H4” refers to the posterior probability of hypothesis 4 from the genetic colocalization analysis.

The SNP proxy was found using the R package “LDlinkR” and the SNP rsid was imputed using the R package “MungeSumstats.” The main MR analyses were performed using the R packages “TwoSampleMR” and “MR‐PRESSO.” The R package “coloc” was used to perform genetic colocalization and the “locuscomparer” was used to plot the locus zoom. The single‐cell analysis was performed using Seurat (4.3.0.1). All statistical analyses and data visualization were conducted using R programing software 4.1.0. The whole study workflow is illustrated in Figure 1.

## 3 RESULTS

Briefly, we identified 16 proteins causally associated with IPF (7 positively associated with IPF and 9 inversely associated with IPF), 6 proteins associated with COPD (2 positively associated with IPF and 4 inversely associated with COPD) and 17 proteins associated with LTL (9 positively associated with IPF and 8 associated with COPD). When using eQTLs as instruments, 8 mRNAs displayed concordant effects on IPF (*FLRT3*, *FN1*, *SELPLG*, *ST3GAL1*, *CCL15*, *SCARF2*, *SERPINA1*, and *UROS*) and 3 on COPD (*APOA5*, *PCSK9*, and *SCARF2*). Therein, the higher levels of *SCARF2* protein should reduce the risk of both IPF (OR = 0.997[0.997, 0.998]) and COPD (OR = 0.743[0.625, 0.883]) and further genetic colocalization implicated that the *SCARF2* protein should be shared with IPF (PPH_4_ = 90.89%) and COPD (PPH_4_ = 99.50%) and such results were still robust when changing priors for IPF (PPH_4_ = 93.20%) and COPD (PPH_4_ = 98.10%). The mediation analysis suggested that the *SCARF2* protein level should affect the risk of IPF and COPD independent of LTL while higher *FN1* protein level should increase the risk of IPF via the way of LTL. The single‐cell transcriptome analysis observed reduced *SCARF2* levels in epithelial cells in COPD lung tissues compared to normal lung tissues.

### 3.1 Identification of serum proteins associated with IPF

**FIGURE 2:**
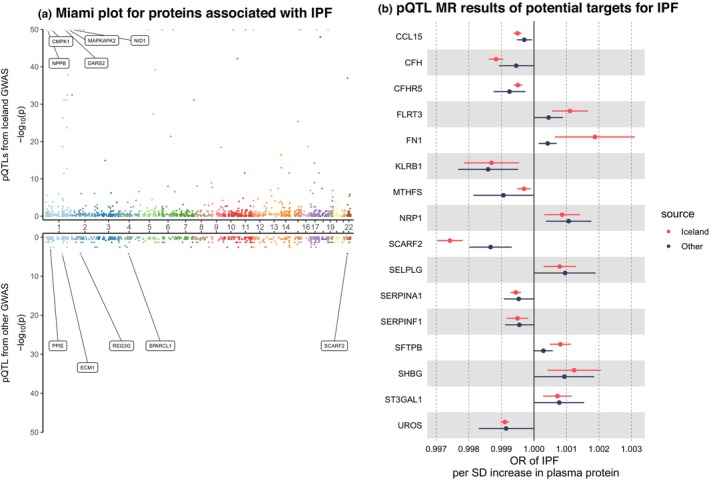
The causal effects of plasma proteins on IPF. (a) Miami plot for proteins associated with IPF. (b) pQTL MR results of potential targets for IPF. IPF, idiopathic pulmonary fibrosis; MR, Mendelian randomization; OR, odds ratio; pQTL, protein quantitative trait locus; SD, standard deviation. In the Miami plot, each point represents a protein and the minimum *p*‐value has been set to 1 × 10^−50^. The “Iceland” represents the results from the discovery stage while “other” represents the results from the validation stage.

After FDR control in the discovery stage and replication in the validation stage, we discovered that genetically‐elevated serum levels of 7 proteins should increase the risk of IPF (*FLRT3*, *FN1*, *NRP1*, *SELPLG*, *SFTPB*, *SHBG*, and *ST3GAL1*) while the genetically‐elevated serum levels of 9 proteins might decrease its risk (*CCL15*, *CFH*, *CFHR5*, *KLRB1*, *MTHFS*, *SCARF2*, *SERPINA1*, *SERPINF1*, and *UROS*) (Figure 2). The genetic colocalization implicated that the locus in the *SCARF2* gene was shared by serum *SCARF2* protein and IPF, suggesting that the *SCARF2* was more likely to be a causal gene of IPF (the posterior probability of H4 was 90.89% using 1966 SNPs) and elevating serum *SCARF2* protein level protect against IPF (Figure 5). Furthermore, the SNP rs9610955 contributed to 86.70% of the total of posterior probability of H4, suggesting that it should be a causal variant in this locus. All associations of plasma proteins with IPF (*p* < 0.05) are displayed in Table S1.

When enriching these proteins in the Reactome database, three pathways were identified, including fibronectin matrix formation, cell surface interactions at the vascular wall, and signaling by receptor tyrosine kinases. The information of DisGeNET suggested that these proteins were closely associated with interstitial lung diseases, pulmonary alveolar proteinosis, spontaneous abortion and neoplasm invasiveness. When mapping these proteins to DrugBank database, 9 potential drugs were discovered, including estriol (DB04824), phenolphthalein (DB00039), palifermin (DB00539), toremifene (DB01094), hesperetin (DB01185), fluoxymesterone (DB02342), 2‐methoxyestradiol (DB11619), and gestrinone (DB01593) (Table S2).

### 3.2 Identification of serum proteins associated with COPD

**FIGURE 3:**
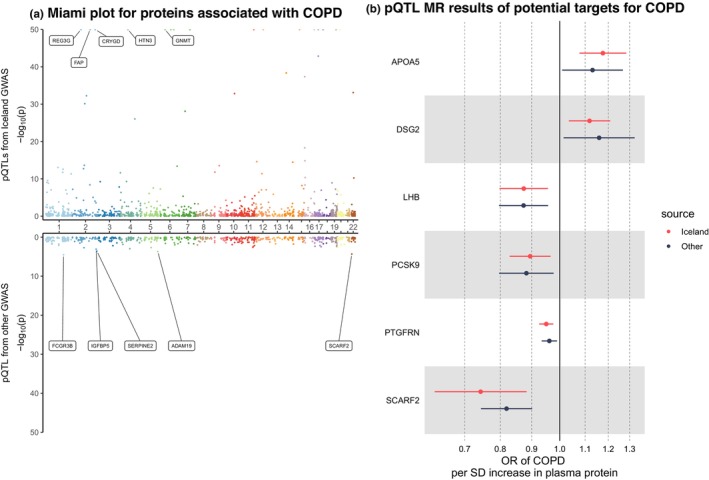
The causal effects of plasma proteins on COPD. (a) Miami plot for proteins associated with COPD. (b) pQTL MR results of potential targets for COPD. COPD, chronic obstructive pulmonary disease; pQTL, protein quantitative trait locus; MR, Mendelian randomization; OR, odds ratio; SD, standard deviation. In the Miami plot, each point represents a protein and the minimum *p*‐value has been set to 1 × 10^−50^. The “Iceland” represents the results from the discovery stage while “other” represents the results from the validation stage.

Genetically‐elevated serum levels of *APOA5* and *DSG2* should increase the risk of COPD while the genetically‐predicted serum levels of *LHB*, *PCSK9*, *PTGFRN*, and *SCARF2* was inversely associated with its risk (Figure 3). The genetic colocalization indicated that both *PTGFRN* (the posterior probability of H4 was 92.57% using 2861 SNPs) and *SCARF2* (the posterior probability of H4 was 99.50% using 2220) were causally associated with COPD, which should be candidate targets for COPD. For *PTGFRN*, two SNPs made substantial contributions to the overall posterior probability of H4, including SNP rs10923180 (54.93%) and rs10923181 (45.07%) (Figure S1). As for the *SCARF2*‐COPD pair, 6 SNPs mainly contributed to the shared locus, including rs738086 (25.03%), rs738087 (22.32%), rs362031 (13.55%), rs361566 (13.29%), rs5763025 (10.67%), and rs9610955 (5.77%) (Figure 5). It should be noted that the *SCARF2* was causally associated with both IPF and COPD and the SNP rs9610955 should be the shared causal variant, highlighting the great potential application of targeting it. All associations of plasma proteins with COPD (*p* < 0.05) are displayed in Table S3.

The top 2 pathways in Reactome database were mineralocorticoid biosynthesis and chylomicron remodeling. The DisGeNET database suggested that these proteins were associated with coronary artery disease (CAD), hypothermia and hypertriglyceridemia (Table S4). The DrugBank did not give potential drugs.

### 3.3 Identification of serum proteins associated with LTL

**FIGURE 4:**
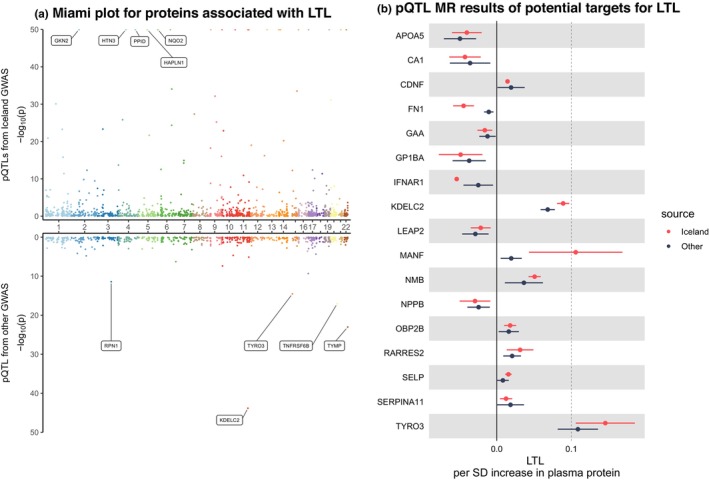
The causal effects of plasma proteins on LTL. (a) Miami plot for proteins associated with LTL. (b) pQTL MR results of potential targets for LTL. LTL, leukocyte telomere length; MR, Mendelian randomization; OR, odds ratio; pQTL, protein quantitative trait locus; SD, standard deviation. In the Miami plot, each point represents a protein and the minimum *p*‐value has been set to 1 × 10^−50^. The “Iceland” represents the results from the discovery stage while “other” represents the results from the validation stage.

We identified 9 proteins positively associated with LTL, including *CDNF*, *KDELC2*, *MANF*, *NMB*, *OBP2B*, *RARRES2*, *SELP*, *SERPINA11*, and *TYRO3* (Figure 4). Besides, genetically‐determined serum levels of 8 proteins were inversely associated with LTL, including *APOA5*, *CA1*, *FN1*, *GAA*, *GP1BA*, *IFNAR1*, *LEAP2*, and *NPPB* (Figure 4). Notably, the genetically‐elevated *FN1* protein level was associated with an increased risk of IPF while be associated with a shorter LTL, and the genetically‐elevated *APOA5* proteins was associated with as increased risk of COPD while being associated with a shorter LTL as well. However, the genetic colocalization did not find robust evidence of shared causal genes. For *FN1*, the highest posterior probability was H1 (92.24%) and so was the *SCARF2* (posterior probability of H1: 55.54%) (Figure S2). For *APOA5*, the highest posterior probability was that of H3 (48.46%), suggesting that the *APOA5* protein and LTL should be affected via different mechanisms (Figure S3). All associations of plasma proteins with LTL (*p* < 0.05) are displayed in Table S5.

**FIGURE 5:**
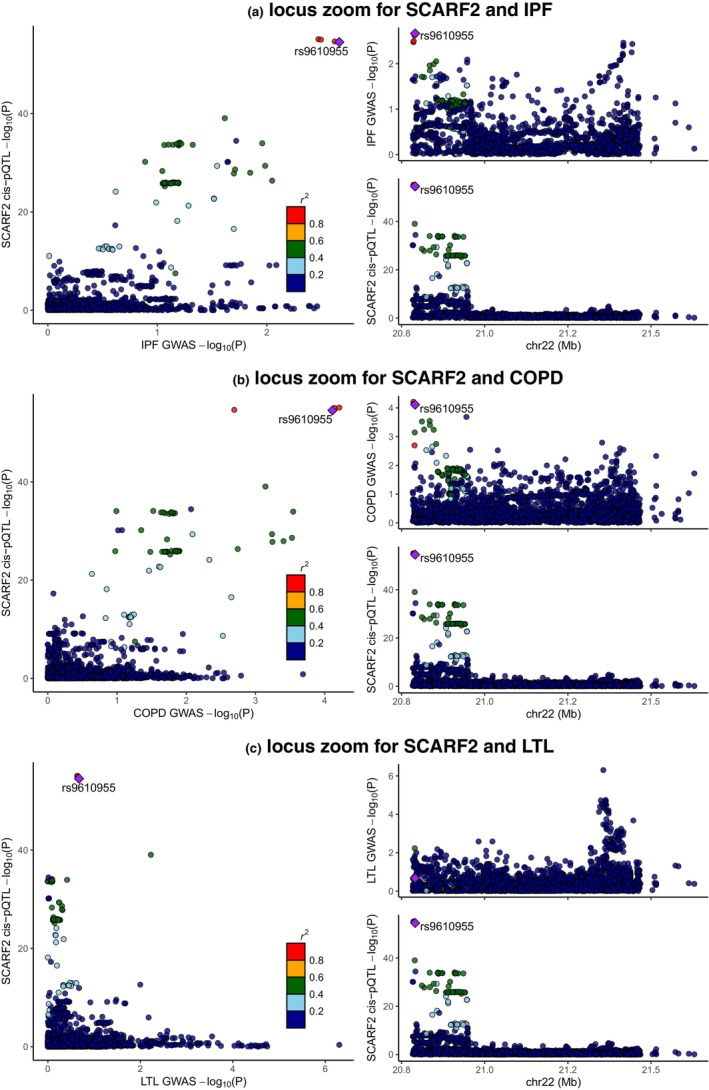
The comparisons of genetic associations in the SACRF2 locus. (a) locus zoom for SCARF2 and IPF. (b) locus zoom for SCARF2 and COPD. (c) locus zoom for SCARF2 and LTL. SCARF2, scavenger receptor class F, member 2; COPD, chronic obstructive pulmonary disease; GWAS, genome‐wide association study; LTL, leukocyte telomere length; IPF, idiopathic pulmonary fibrosis. Each point represents a single nucleotide polymorphism (SNP) and the *r* ^2^ is the measurement of linkage disequilibrium. The original *p*‐value was transformed using −log_10_. The purple SNP rs9610955 is the index SNP of SCARF2.

**FIGURE 6:**
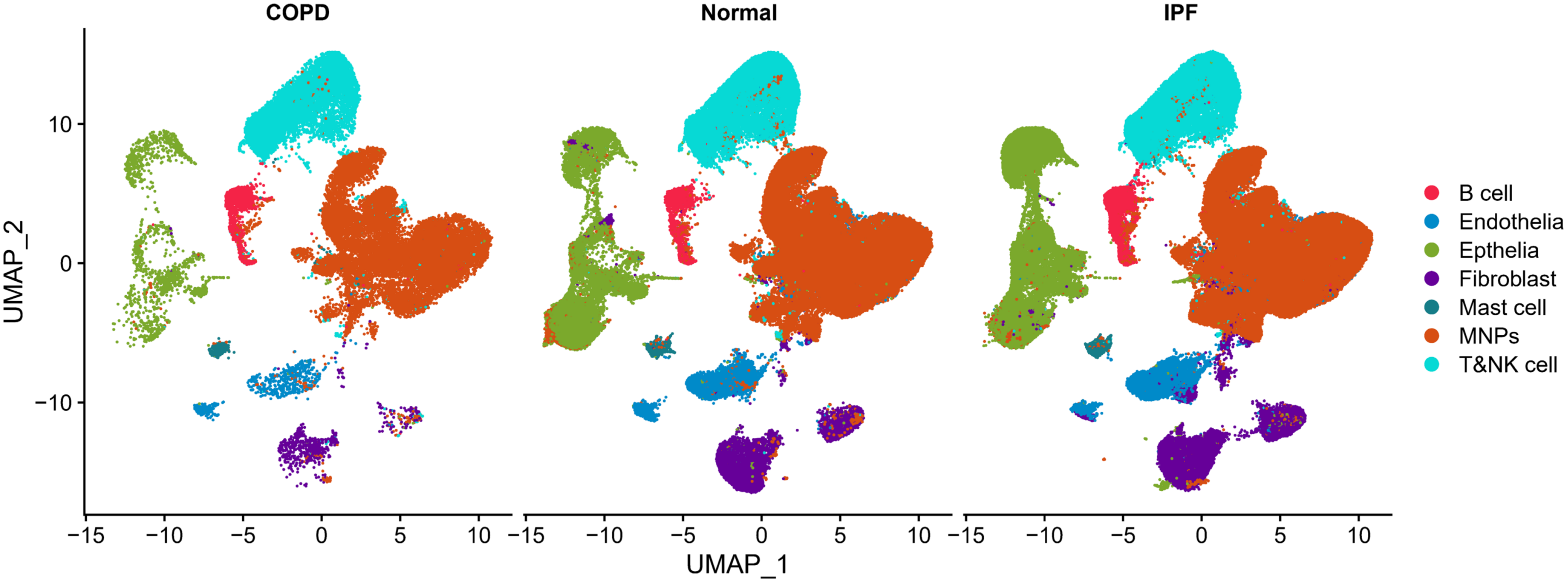
Single‐cell transcriptome data analysis of *SCARF2* levels in COPD, normal and IPF lung tissues. COPD, chronic obstructive pulmonary disease; IPF, idiopathic pulmonary fibrosis; MNP, mononuclear phagocytes; UMAP, uniform manifold approximation and projection.

In the Reactome database, the main pathways are closely associated with fibronectin matrix formation and platelet activation, signaling and aggregation. Furthermore, these genes are mainly associated with thrombosis, aortic valve Insufficiency, left ventricular hypertrophy dyspnea, and coronary artery disease. The DrugBank gave five potential drugs, including miglitol (DB00491), hydrochlorothiazide (DB00999), quinethazone (DB01325), dalteparin (DB06779), and zinc (DB01593) (Table S6).

### 3.4 Effects of mRNA levels on IPF and COPD for significant targets

For 16 proteins associated with IPF, 8 corresponding mRNA levels displayed concordant causal effects where the directions of eQTLs/pQTLs were the same and 3 corresponding mRNA levels were consistent with protein levels for COPD (Table 1). The genetically‐elevated mRNA levels of *FLRT3*, *FN1*, *SELPLG*, and *ST3GAL1* were associated with an increased risk of IPF while the risk of IPF decreased with the elevation of *CCL15*, *SCARF2*, *SERPINA1*, and *UROS*. For COPD, higher mRNA level of *APOA5* should increase its risk while higher mRNA levels of *PCSK9* and *SCARF2* might reduce such a risk. Concordant evidence implicated that these targets should strongly have a causal link to IPF or COPD.

**TABLE 1:** Mendelian randomization analysis and colocalization of circulating proteins with idiopathic pulmonary fibrosis and chronic obstructive pulmonary disease.

Proteins	Mendelian randomization using pQTL				Mendelian randomization using eQTL				Colocalization (PH4, %)
	Discovery		Validation		Discovery		Validation		
	OR (95% CI)	P	OR (95% CI)	P	OR (95%CI)	P	OR (95% CI)	P	
IPF									
*FLRT3*	1.0011 (1.0006, 1.0017)	9.70E‐05	1.0005 (1.0001, 1.0009)	4.55E‐02	1.2524 (1.1591, 1.3532)	1.21E‐08	1.4197 (1.2003, 1.6791)	4.27E‐05	0.08
*FN1*	1.0019 (1.0007, 1.0031)	2.70E‐03	1.0004 (1.0001, 1.0007)	2.70E‐03	1.2760 (1.2171, 1.3378)	5.10E‐24	1.5282 (1.2476, 1.8720)	4.19E‐05	0.15
*NRP1*	1.0009 (1.0003, 1.0014)	1.88E‐03	1.0011 (1.0004, 1.0018)	2.70E‐03	0.4476 (0.2110, 0.9496)	3.62E‐02	0.8511 (0.7306, 0.9914)	3.84E‐02	4.11
*SELPLG*	1.0008 (1.0003, 1.0013)	1.82E‐03	1.0010 (1.0001, 1.0019)	4.55E‐02	14.7814 (5.5224, 39.5644)	8.24E‐08	4.6242 (2.2142, 9.6575)	4.59E‐05	0.37
*SFTPB*	1.0008 (1.0005, 1.0011)	8.80E‐07	1.0003 (1.0001, 1.0006)	4.55E‐02	0.9090 (0.8440, 0.9791)	1.18E‐02	0.7275 (0.5295, 0.9997)	4.98E‐02	0.36
*SHBG*	1.0012 (1.0004, 1.0021)	3.14E‐03	1.0009 (1.0001, 1.0019)	4.55E‐02	1.0957 (0.9755, 1.2307)	1.23E‐01	0.8894 (0.8403, 0.9414)	5.22E‐05	0.37
*ST3GAL1*	1.0007 (1.0003, 1.0012)	1.29E‐03	1.0008 (1.0001, 1.0015)	4.55E‐02	1.1158 (1.0674, 1.1664)	1.29E‐06	1.2258 (1.1102, 1.3535)	5.61E‐05	0.42
*CCL15*	0.9995 (0.9994, 0.9996)	6.19E‐15	0.9997 (0.9995, 0.9999)	1.24E‐02	0.9370 (0.9125, 0.9622)	1.51E‐06	0.6866 (0.5716, 0.8249)	5.88E‐05	0.92
*CFH*	0.9988 (0.9986, 0.9991)	1.98E‐24	0.9995 (0.9989, 0.9999)	4.55E‐02	0.8681 (0.7137, 1.0559)	1.57E‐01	1.3424 (1.0789, 1.6702)	8.26E‐03	0.46
*CFHR5*	0.9995 (0.9994, 0.9996)	1.70E‐13	0.9993 (0.9988, 0.9997)	2.70E‐03	1.2688 (1.1512, 1.3985)	1.62E‐06	1.3094 (1.1168, 1.5352)	8.96E‐04	0.08
*KLRB1*	0.9987 (0.9978, 0.9995)	2.70E‐03	0.9986 (0.9977, 0.9995)	2.70E‐03	1.2326 (1.0392, 1.4620)	1.63E‐02	1.3053 (0.9566, 1.7812)	9.29E‐02	4.28
*MTHFS*	0.9997 (0.9995, 0.9999)	3.30E‐03	0.9991 (0.9981, 0.9999)	4.55E‐02	1.3584 (1.1099, 1.6625)	2.96E‐03	1.8241 (0.8498, 3.9157)	1.23E‐01	0.37
*SCARF2*	0.9974 (0.9970, 0.9978)	1.07E‐37	0.9987 (0.9980, 0.9993)	6.33E‐05	0.8108 (0.7425, 0.8854)	3.02E‐06	0.8950 (0.8457, 0.9472)	1.25E‐04	90.89
*SERPINA1*	0.9994 (0.9993, 0.9996)	2.56E‐12	0.9995 (0.9991, 0.9999)	4.55E‐02	0.7895 (0.7116, 0.8758)	8.08E‐06	0.5534 (0.4086, 0.7497)	1.33E‐04	0.37
*SERPINF1*	0.9995 (0.9992, 0.9998)	2.99E‐03	0.9996 (0.9991, 0.9999)	4.55E‐02	0.7987 (0.6165, 1.0348)	8.89E‐02	0.5498 (0.2489, 1.2144)	1.39E‐01	0.35
*UROS*	0.9991 (0.9990, 0.9992)	6.41E‐43	0.9991 (0.9983, 0.9999)	4.55E‐02	0.9201 (0.8868, 0.9547)	9.62E‐06	0.6756 (0.5515, 0.8276)	1.52E‐04	0.11
COPD									
*APOA5*	1.1759 (1.0771, 1.2837)	2.94E‐04	1.1311 (1.0092, 1.2677)	3.42E‐02	1.1802 (1.0965, 1.2702)	1.01E‐05	2.4766 (1.5457, 3.9682)	1.63E‐04	12.70
*DSG2*	1.1184 (1.0348, 1.2088)	4.75E‐03	1.1594 (1.0147, 1.3249)	2.97E‐02	0.8953 (0.7833, 1.0234)	1.05E‐01	0.7756 (0.6622, 0.9084)	1.63E‐03	1.73
*LHB*	0.8737 (0.7978, 0.9568)	3.59E‐03	0.8731 (0.7962, 0.9574)	3.89E‐03	1.8011 (0.8682, 3.7364)	1.14E‐01	0.8030 (0.5805, 1.1107)	1.85E‐01	65.43
*PCSK9*	0.8949 (0.8286, 0.9664)	4.65E‐03	0.8824 (0.7965, 0.9774)	1.65E‐02	0.7939 (0.7160, 0.8803)	1.19E‐05	0.6053 (0.4644, 0.7890)	2.05E‐04	25.08
*PTGFRN*	0.9506 (0.9260, 0.9760)	1.60E‐04	0.9618 (0.9345, 0.9899)	7.98E‐03	0.8462 (0.6863, 1.0433)	1.18E‐01	0.5819 (0.2528, 1.3394)	2.03E‐01	92.57
*SCARF2*	0.7431 (0.6253, 0.8831)	7.47E‐04	0.8190 (0.7440, 0.9016)	4.60E‐05	0.8454 (0.7841, 0.9114)	1.20E‐05	0.9464 (0.9191, 0.9745)	2.21E‐04	99.50

Abbreviations: CI, confidence interval; COPD, chronic obstructive pulmonary disease; eQTL, expression quantitative trait locus; IPF, idiopathic pulmonary fibrosis; OR, odds ratio; *p*, the *p*‐value of odds ratio; pQTL, protein quantitative trait locus.

### 3.5 Mediation effects of LTL on IPF and COPD

We estimated whether the LTL mediated the causal effects of serum proteins on IPF and COPD for three overlapped proteins *SCARF2*, *FN1*, and *APOA5*. Preliminarily, we fitted the multivariable MR (MVMR) model where IPF/COPD was the outcome and the protein and LTL were the exposures. The MVMR suggested that only the genetically‐predicted *SCARF2* protein level was significantly associated with the risk of IPF (OR = 0.9981 [0.9962, 0.9991]) and COPD (OR = 0.7610 [0.6331, 0.9142]) after adjusting for LTL. The indirect effects of *SCARF2* protein level on IPF/COPD were not significant (IPF: OR = 1.0001 [0.0003, 1.3150 × 10^4^]; COPD: OR = 1.0224 [0.5880, 1.6151]). The genetically‐predicted *FN1* and *APOA5* protein levels were not associated with either IPF or COPD after adjusting for LTL; however, the indirect effect of *FN1* on IPF was significant (OR = 1.1740 [1.0271, 1.3360]).

### 3.6 Phenome‐wide association screening for *SCARF2* , *FN1* , and *APOA5*

In the open GWAS database, we identified that 3 genetic variants located in the *SCARF2* gene region were associated with lung function (FEV1/FVC) (Shrine et al., 2019), namely rs5763025‐C, rs2108746‐G, and rs9619753‐A (C, G and A are the effect‐increasing allele). Therein, SNP rs5763025 and rs2108746 are in high LD (R^2^ = 0.993). Besides, the *SCARF2* variants were associated with FEV1 as well (Table S7). Such results implicated that the *SCARF2* gene should be associated with lung function. Two SNPs in the *FN1* gene region (rs13423742‐C and rs1250258‐T) were associated with peak expiratory flow. No *APOA5* variant was associated with lung function or lung‐associated phenotypes.

### 3.7 Low expression of *SCARF2* in epithelial cells in COPD lung tissues

A total of 716,074 cells derived from normal (*n* = 28), COPD (*n* = 18), and IPF (*n* = 32) have been integrated to characterize the expression of *SCARF2*. The analysis of this dataset demonstrated that *SCARF2* was mainly expressed in epithelial cells and *SCARF2* expression in COPD patients reduced significantly, compared to fibrotic and normal patients (Figure 6). However, the difference of *SCARF2* levels between IPF and normal lung tissues was not significant. Such results supported the possibility of targeting *SCARF2* in lung epithelia to treat COPD.

### 3.8 Inverse associations of serum *SCARF2* protein with COPD in UKB

A total of 47,410 participants with both clinical and protein information were included in the analysis and 3909 developed COPD. The baseline level of serum *SCARF2* was inversely associated with the COPD risk (HR = 1.215 [1.106, 1.335]). The association of *SCARF2* with IPF (549 cases) was not significant in the Cox model (HR = 1.546 [0.814, 2.936]). These results highlighted the role of lower *SCARF2* protein in the pathogenesis of COPD.

## 4 DISCUSSION

This study gave novel insights into the potential targets for IPF and COPD using multi‐omics data and highlighted the potential value of elevating *SCARF2* level to decrease the risk COPD. Besides, the LTL can be used to explain the part of possible mechanisms, for example, cellular senescence.

The *SCARF2* (scavenger receptor class F, member 2) protein is a scavenger receptor protein that mediates the binding and degradation of acetylated low‐density lipoprotein, which is similar to *SCARF1* (Ishii et al., 2002). Currently, the biological function of *SCARF2* has not been sufficiently explored. A well‐established disease association is van den Ende‐Gupta syndrome (VDEGS) and several missense mutations on it have been identified (Anastasio et al., 2010; Karaer & Karaer, 2022). Besides, this gene has been reported to be associated with glioblastoma, gastric cancer and mood disorders (Kim et al., 2022; Vysotskiy et al., 2021; Zhao et al., 2013). However, such associations with cancer and mood disorders need further validation. No studies directly pinpointed the role *SCARF2* in COPD or IPF and only one study suggested that the higher plasma level of *SCARF2* protein should increase FEV1/FVC (*β* = 0.300, *p* = 4.79 × 10^−21^) and such a result was consistent with that of phenome‐wide screening, implicating the higher *SCARF2* protein should alleviate the symptoms of COPD and IPF (Shrine et al., 2019). Furthermore, the SNP rs9610955 in the *SCARF2* gene is closely associated with lung function and it might be the causal variant as well according to the genetic colocalization (Shrine et al., 2019). Intriguingly, a recent small‐sample study identified that the *SCARF2* protein was downregulated in the nasal mucus of chronic rhinosinusitis without nasal polyps (Pesold et al., 2022). This study should be informative as the nasal sinus is a part of upper respiratory tract and the chronic rhinosinusitis shared the basic pathological mechanism (inflammation) with COPD and IPF.

According to our results, the plasma level of *SCARF2* protein was lower in both COPD and IPF, which was corroborated by gene expression data and individual‐level data from UKB. Also, we observed that such associations were significant after adjusting for LTL, but we did not observe a direct association between *SCARF2* and LTL. The single‐cell transcriptome analysis suggested that *SCARF2* was lowly expressed in epithelium cells in COPD lung tissues compared to normal lung tissues. Thus, we hypothesized that increasing *SCARF2* protein level should be a candidate therapeutic for COPD via the way of alleviating inflammation and reversing the modelling of lung tissues. However, the overexpression of *SCARF2* was associated with the increased risk of cancer (Kim et al., 2022), and the safety of elevating *SCARF2* protein should be well‐assessed in further drug development and clinical investigations.

The *FN1* (fibronectin 1) is a profibrotic factor that involves in cell adhesion and migration processes, and its deposition in the lung interstitium is the main characteristic of IPF (Bueno et al., 2020). In this study, we discovered that the higher plasma level of *FN1* protein might increase the risk of IPF while shortening the LTL. However, such an association was not significant after adjusting for LTL, and the its indirect effect on IPF mediated by LTL is significant. It was probable that the higher plasma of *FN1* protein should shorten LTL and further increase the risk of IPF since shorter LTL was casually associate with an increased IPF risk (Duckworth et al., 2021). The *FN1* has been shown to promote the recruitment of immune cells to sites of inflammation and to facilitate their activation and proliferation (Heerspink et al., 2019). It can also interact with other molecules involved in inflammation, such as integrins, growth factors, and extracellular matrix proteins. Meanwhile, the inflammation status caused by life stress and cigarette smoking should shorten the LTL (Epel et al., 2004; Valdes et al., 2005). Thus, the higher plasma level of *FN1* protein might shorten the LTL via the inflammation possibly. Combining all the evidence, we postulated that it is feasible to prevent and ameliorate IPF via the way of inhibiting *FN1* or maintaining LTL.

The genetic variants in the *APOA5* (apolipoprotein A5) gene should upregulate the serum triglyceride levels via an increased plasma *APOA5* concentration and contribute to the increased risk of coronary heart disease (CHD) (Cui et al., 2014), which is frequently associated with COPD and these two diseases share common risk factors, pathophysiological processes, and clinical manifestations (André et al., 2019). Our MR analyses suggested that higher plasma level of *APOA5* is causally associated with an increased risk of COPD and a shorter LTL, and the effect of *APOA5* on COPD might not be mediated by LTL. A meta‐analysis implicated that higher levels of triglycerides were associated with a higher risk of COPD (Xuan et al., 2018). What's more, a recent study suggested that triglyceride‐glucose index was associated with chronic lung diseases and might be a good measure of pulmonary outcomes combined with metabolic dysfunction (Wu et al., 2021). This may be due to the fact that triglycerides can contribute to inflammation and oxidative stress, which are known to play a role in COPD pathogenesis. Thus, it is plausible that the higher plasma level of *APOA5* protein might increase the levels of triglycerides, and further contributed to the pathogenesis of COPD. Besides, triglycerides should be closely associated with CHD and metabolic dysfunctions, suggesting that *APOA5* might be a candidate target for COPD, CHD and metabolic dysfunctions.

Aside from *SCARF2*, *FN1*, and *APOA5*, we also discovered several candidate targets that might work for treating IPF or COPD. For example, the higher plasma level of *FLRT3* (fibronectin leucine rich transmembrane protein 3) was causally associated with an increased risk of IPF, which was similar to FN1, and the top pathway is fibronectin matrix formation, suggesting targeting this pathway should ameliorate IPF. As for COPD, we unexpectedly observed an inverse association of plasma level of *PCSK9* (proprotein convertase subtilisin/kexin type 9) protein with COPD risk as the association of higher plasma level of *PCSK9* protein with CHD has been well established; however, the association of *PCSK9* with COPD is still unknown. What's more, we discovered that the significant proteins were mainly involved in mineralocorticoid biosynthesis and chylomicron remodeling, and they were closely associated with cardiovascular diseases (CVD). Such results indicate that it is appropriate to balancing the lipid metabolism to ameliorate COPD and it is probable that COPD and CVD should be considered together in clinical practice.

Our study leveraged the current transcriptome, proteome and genome data with the largest sample size to identify candidate targets for two ageing‐related chronic inflammatory lung disease (IPF and COPD) in a robust way, and furthermore explained the mediation effects of LTL on them. This study gave not only association information, but also provided mechanistic insights. Despite of these, several cautions should be pointed out to help further investigations: (1) this study only used cis‐pQTL as the instrument and it can reduce the potential bias caused by horizontal pleiotropy, however, horizontal pleiotropy cannot be completely eliminated and the results should be interpreted with caution; (2) over 2000 proteins were included in the discovery stage while the number of proteins in the validation stage was less than 1000, implicating that some potential targets might not be identified as they were missing in the validation stage; (3) all summary statistics were mainly obtained from European GWAS and it can reduce the bias caused by population stratification, but it can limit the extrapolation of our findings to other populations. What's more, the genetic characteristics of Iceland individuals is different from that of European mainland individuals. At last, it should be noted that the identified target in this study can be informative in disease prevention and its role in disease treatment should be investigated in the future.

In a word, we have identified a robust target *SCARF2* for COPD. Besides, we demonstrated that higher plasma level of *FN1* protein should probably affect the risk of IPF via the way of shortening LTL.

## 5 CONCLUSION

The *SCARF2* gene should be a novel target for COPD.

## AUTHOR CONTRIBUTIONS

Qiang Zhang conceived and designed the paper; Sai Wang, Yuanyi Yue and Xueqing Wang analyzed the data; Yuanyi Yue, Xueqing Wang and Yue Tan contributed reagents/materials/analysis tools; Sai Wang drafted the manuscript and all the other authors reviewed and revised the manuscript. Qiang Zhang is responsible for the integrity of the entire research.

## FUNDING INFORMATION

This study was supported by the grants from Department of Science and Technology of Liaoning Province (No. 2018225006), Shenyang Science and Technology Plan Project (No. 21‐173‐9‐43) and 345 Talent Project of Shengjing Hospital.

## CONFLICT OF INTEREST STATEMENT

All authors declared that no potential conflicts of interest should be disclosed in this study. All authors gave consent to the publication of this work.

## Supporting information


Data S1:

## Data Availability

All GWAS summary statistics are publicly available and can be accessed freely. The pQTL information from Zheng et al. can be obtained from their publication's supplementary information. The pQTL information from Iceland can be obtained from the website https://www.decode.com/summarydata/. The eQTL data can be obtained from eQTLGen database (https://www.eqtlgen.org/) and GTEx database (https://www.gtexportal.org/home/index.html). The single‐cell transcriptome data can be found from https://singlecell.broadinstitute.org/single_cell/study/SCP2155/. The UK Biobank data can be accessed via the link https://www.ukbiobank.ac.uk/.
