# Supplementary material for: SCARF2 is a target for chronic obstructive pulmonary disease: Evidence from multi‐omics research and cohort validation
Source: Aging Cell. 2024 Jul 3;23(10):e14266. doi: 10.1111/acel.14266 (PMC11464143; doi:10.1111/acel.14266)

**Supplementary Table 1: Associations of circulation proteins with idiopathic pulmonary fibrosis.**

**Supplementary Table 2: The results of enrichment analyses using circulating proteins closely associated with idiopathic pulmonary fibrosis.**

**Supplementary Table 3: Associations of circulation proteins with chronic obstructive pulmonary disease.**

**Supplementary Table 4: The results of enrichment analyses using circulating proteins closely associated with chronic obstructive pulmonary disease.**

**Supplementary Table 5: Associations of circulation proteins with leukocyte telomere length.**

**Supplementary Table 6: The results of enrichment analyses using circulating proteins closely associated with leukocyte telomere length.**

**Supplementary Table 7:** **Phenome-wide screening for traits associated with variants located in the target genes.**

**Supplementary Figure 1: The comparisons of genetic associations in the PTGFRN locus. A. locus zoom for PTGFRN and IPF. B. locus zoom for PTGFRN and COPD. C. locus zoom for PTGFRN and LTL.** Notes: PTGFRN = Prostaglandin F2 Receptor Inhibitor; IPF = idiopathic pulmonary fibrosis; COPD = chronic obstructive pulmonary disease; LTL = leukocyte telomere length; GWAS = genome-wide association study. Each point represents a single nucleotide polymorphism (SNP) and the r2 is the measurement of linkage disequilibrium. The original p-value was transformed using -log10.

**Supplementary Figure 2: The comparisons of genetic associations in the FN1 locus. A. locus zoom for FN1 and IPF. B. locus zoom for FN1 and COPD. C. locus zoom for FN1 and LTL.** Notes: FN1 = Fibronectin 1; IPF = idiopathic pulmonary fibrosis; COPD = chronic obstructive pulmonary disease; LTL = leukocyte telomere length; GWAS = genome-wide association study. Each point represents a single nucleotide polymorphism (SNP) and the r2 is the measurement of linkage disequilibrium. The original p-value was transformed using -log10.

**Supplementary Figure 3: The comparisons of genetic associations in the APOA5 locus. A. locus zoom for APOA5 and IPF. B. locus zoom for APOA5 and COPD. C. locus zoom for APOA5 and LTL.** Notes: APOA5 = Apolipoprotein A5; IPF = idiopathic pulmonary fibrosis; COPD = chronic obstructive pulmonary disease; LTL = leukocyte telomere length; GWAS = genome-wide association study. Each point represents a single nucleotide polymorphism (SNP) and the r2 is the measurement of linkage disequilibrium. The original p-value was transformed using -log10.

**Supplementary Table 1: Associations of circulation proteins with idiopathic pulmonary fibrosis.**

| **Proteins** | **Methods** | **NSNP** | **OR** | **Lower of 95%CI** | **Upper of 95%CI** | **P** | **P_het_** | **P_pleio_** | **Stage** |
| --- | --- | --- | --- | --- | --- | --- | --- | --- | --- |
| ACAP2 | Wald ratio | 1 | 0.9967 | 0.9934 | 0.9999 | 4.55E-02 | NA | NA | Discovery |
| AGER | Weighted median | 3 | 0.9995 | 0.9990 | 1.0000 | 4.54E-02 | NA | NA | Discovery |
| AK1 | Wald ratio | 1 | 0.9934 | 0.9901 | 0.9966 | 6.33E-05 | NA | NA | Discovery |
| ALDH3B1 | Inverse variance weighted | 2 | 0.9993 | 0.9992 | 0.9994 | 8.02E-80 | NA | NA | Discovery |
| ALPPL2 | Inverse variance weighted | 4 | 1.0011 | 1.0003 | 1.0019 | 5.36E-03 | 0.3628 | 0.8442 | Discovery |
| ALPPL2 | Weighted median | 4 | 1.0012 | 1.0004 | 1.0021 | 4.18E-03 | 0.3628 | 0.8442 | Discovery |
| ANGPTL3 | Wald ratio | 1 | 0.9991 | 0.9982 | 1.0000 | 4.55E-02 | NA | NA | Discovery |
| ANKRD27 | Wald ratio | 1 | 1.0060 | 1.0021 | 1.0100 | 2.70E-03 | NA | NA | Discovery |
| ANTXR2 | Inverse variance weighted | 4 | 0.9994 | 0.9990 | 0.9997 | 2.04E-04 | 0.7732 | 0.4576 | Discovery |
| ANXA2 | Inverse variance weighted | 6 | 1.0003 | 1.0000 | 1.0006 | 2.21E-02 | 0.7586 | 0.4906 | Discovery |
| AOC2 | Inverse variance weighted | 2 | 0.9989 | 0.9979 | 0.9999 | 3.21E-02 | NA | NA | Discovery |
| AP1G2 | Inverse variance weighted | 2 | 0.9995 | 0.9994 | 0.9996 | 1.06E-13 | NA | NA | Discovery |
| APOM | Inverse variance weighted | 3 | 1.0015 | 1.0005 | 1.0025 | 2.70E-03 | NA | NA | Discovery |
| APOM | Weighted median | 3 | 1.0015 | 1.0003 | 1.0027 | 1.31E-02 | NA | NA | Discovery |
| ARL4D | Wald ratio | 1 | 0.1419 | 0.0251 | 0.8005 | 2.70E-02 | NA | NA | Discovery |
| ARSB | Inverse variance weighted | 2 | 0.9992 | 0.9991 | 0.9993 | 6.11E-138 | NA | NA | Discovery |
| ARSK | Wald ratio | 1 | 0.9970 | 0.9941 | 0.9999 | 4.55E-02 | NA | NA | Discovery |
| ATRN | Wald ratio | 1 | 1.0009 | 1.0000 | 1.0018 | 4.55E-02 | NA | NA | Discovery |
| BDH2 | Wald ratio | 1 | 1.0012 | 1.0000 | 1.0023 | 4.55E-02 | NA | NA | Discovery |
| BGN | Inverse variance weighted | 2 | 0.9977 | 0.9973 | 0.9980 | 4.51E-40 | NA | NA | Discovery |
| BOC | Inverse variance weighted | 3 | 1.0008 | 1.0002 | 1.0013 | 6.01E-03 | NA | NA | Discovery |
| BPI | Inverse variance weighted | 3 | 1.0001 | 1.0000 | 1.0002 | 6.84E-03 | NA | NA | Discovery |
| BPNT1 | Wald ratio | 1 | 1.0057 | 1.0001 | 1.0114 | 4.55E-02 | NA | NA | Discovery |
| BST1 | Wald ratio | 1 | 0.4858 | 0.2396 | 0.9849 | 4.53E-02 | NA | NA | Discovery |
| BTNL8 | Wald ratio | 1 | 0.9982 | 0.9964 | 1.0000 | 4.55E-02 | NA | NA | Discovery |
| C10orf54 | Inverse variance weighted | 3 | 1.0002 | 1.0000 | 1.0004 | 3.70E-02 | NA | NA | Discovery |
| C1GALT1C1 | Inverse variance weighted | 2 | 0.9990 | 0.9982 | 0.9998 | 1.02E-02 | NA | NA | Discovery |
| C1QC | Inverse variance weighted | 7 | 1.0003 | 1.0000 | 1.0006 | 3.79E-02 | 0.8132 | 0.8197 | Discovery |
| C1QL1 | Wald ratio | 1 | 1.0014 | 1.0005 | 1.0024 | 2.70E-03 | NA | NA | Discovery |
| CA10 | Inverse variance weighted | 4 | 0.9990 | 0.9982 | 0.9999 | 3.54E-02 | 0.3035 | 0.2223 | Discovery |
| CADM1 | Wald ratio | 1 | 1.0013 | 1.0000 | 1.0026 | 4.55E-02 | NA | NA | Discovery |
| CCDC126 | Inverse variance weighted | 3 | 0.9994 | 0.9990 | 0.9999 | 1.31E-02 | NA | NA | Discovery |
| CCDC126 | Weighted median | 3 | 0.9994 | 0.9990 | 0.9999 | 2.55E-02 | NA | NA | Discovery |
| CCL14 | Inverse variance weighted | 2 | 1.0005 | 1.0003 | 1.0006 | 1.06E-09 | NA | NA | Discovery |
| CCL15 | Inverse variance weighted | 2 | 0.9995 | 0.9994 | 0.9996 | 6.19E-15 | NA | NA | Discovery |
| CCL18 | Inverse variance weighted | 4 | 0.9995 | 0.9992 | 0.9998 | 3.56E-03 | 0.8993 | 0.7488 | Discovery |
| CCL25 | Inverse variance weighted | 3 | 0.9996 | 0.9996 | 0.9997 | 5.74E-65 | NA | NA | Discovery |
| CCL3 | Inverse variance weighted | 2 | 0.9997 | 0.9997 | 0.9998 | 6.19E-15 | NA | NA | Discovery |
| CCL5 | Wald ratio | 1 | 1.0015 | 1.0000 | 1.0031 | 4.55E-02 | NA | NA | Discovery |
| CCT5 | Wald ratio | 1 | 0.9947 | 0.9895 | 0.9999 | 4.55E-02 | NA | NA | Discovery |
| CD14 | Inverse variance weighted | 2 | 1.0003 | 1.0000 | 1.0007 | 4.55E-02 | NA | NA | Discovery |
| CD46 | Inverse variance weighted | 2 | 1.0008 | 1.0006 | 1.0009 | 1.22E-38 | NA | NA | Discovery |
| CD58 | Inverse variance weighted | 2 | 1.0006 | 1.0004 | 1.0009 | 9.77E-07 | NA | NA | Discovery |
| CD5L | Inverse variance weighted | 3 | 0.9997 | 0.9995 | 0.9999 | 2.71E-03 | NA | NA | Discovery |
| CD72 | Inverse variance weighted | 2 | 1.0011 | 1.0007 | 1.0014 | 1.92E-08 | NA | NA | Discovery |
| CDH5 | Inverse variance weighted | 2 | 0.9985 | 0.9982 | 0.9989 | 2.26E-19 | NA | NA | Discovery |
| CFH | Inverse variance weighted | 2 | 0.9988 | 0.9986 | 0.9991 | 1.98E-24 | NA | NA | Discovery |
| CFHR1 | Inverse variance weighted | 2 | 0.9986 | 0.9984 | 0.9988 | 3.55E-33 | NA | NA | Discovery |
| CFHR4 | Wald ratio | 1 | 1.0011 | 1.0002 | 1.0021 | 1.96E-02 | NA | NA | Discovery |
| CFHR5 | Inverse variance weighted | 2 | 0.9995 | 0.9994 | 0.9996 | 1.70E-13 | NA | NA | Discovery |
| CFI | Wald ratio | 1 | 1.0011 | 1.0000 | 1.0021 | 4.55E-02 | NA | NA | Discovery |
| CFP | Wald ratio | 1 | 1.0025 | 1.0001 | 1.0050 | 4.55E-02 | NA | NA | Discovery |
| CHL1 | Weighted median | 5 | 0.9992 | 0.9985 | 0.9999 | 2.77E-02 | 0.3420 | 0.3838 | Discovery |
| CHST9 | Inverse variance weighted | 2 | 0.9996 | 0.9994 | 0.9997 | 3.80E-08 | NA | NA | Discovery |
| CLN5 | Inverse variance weighted | 2 | 1.0005 | 1.0002 | 1.0007 | 8.80E-05 | NA | NA | Discovery |
| CLSTN1 | Wald ratio | 1 | 0.9980 | 0.9964 | 0.9996 | 1.24E-02 | NA | NA | Discovery |
| CMBL | Wald ratio | 1 | 0.9983 | 0.9967 | 1.0000 | 4.55E-02 | NA | NA | Discovery |
| CMPK1 | Inverse variance weighted | 2 | 0.9982 | 0.9980 | 0.9984 | 1.23E-95 | NA | NA | Discovery |
| CNRIP1 | Wald ratio | 1 | 1.0009 | 1.0000 | 1.0018 | 4.55E-02 | NA | NA | Discovery |
| COCH | Inverse variance weighted | 3 | 0.9972 | 0.9956 | 0.9987 | 4.54E-04 | NA | NA | Discovery |
| COCH | Weighted median | 3 | 0.9972 | 0.9943 | 1.0000 | 4.95E-02 | NA | NA | Discovery |
| COL6A2 | Inverse variance weighted | 7 | 0.9992 | 0.9985 | 0.9999 | 2.06E-02 | 0.5365 | 0.2705 | Discovery |
| COL6A3 | Inverse variance weighted | 3 | 1.0012 | 1.0003 | 1.0021 | 8.63E-03 | NA | NA | Discovery |
| COLEC12 | Inverse variance weighted | 5 | 0.9990 | 0.9980 | 0.9999 | 3.12E-02 | 0.3694 | 0.4397 | Discovery |
| COLEC12 | Weighted median | 5 | 0.9987 | 0.9975 | 1.0000 | 4.15E-02 | 0.3694 | 0.4397 | Discovery |
| CPB2 | Inverse variance weighted | 2 | 1.0008 | 1.0005 | 1.0012 | 4.22E-06 | NA | NA | Discovery |
| CPOX | Inverse variance weighted | 3 | 1.0016 | 1.0005 | 1.0026 | 2.84E-03 | NA | NA | Discovery |
| CPOX | Weighted median | 3 | 1.0019 | 1.0004 | 1.0034 | 1.20E-02 | NA | NA | Discovery |
| CPXM1 | Inverse variance weighted | 4 | 0.9997 | 0.9995 | 0.9999 | 9.37E-03 | 0.8850 | 0.8873 | Discovery |
| CRAT | Inverse variance weighted | 2 | 1.0017 | 1.0005 | 1.0029 | 6.20E-03 | NA | NA | Discovery |
| CREG1 | Inverse variance weighted | 3 | 0.9984 | 0.9979 | 0.9988 | 3.53E-12 | NA | NA | Discovery |
| CREG1 | Weighted median | 3 | 0.9984 | 0.9973 | 0.9994 | 1.65E-03 | NA | NA | Discovery |
| CRHBP | Wald ratio | 1 | 0.9982 | 0.9965 | 1.0000 | 4.55E-02 | NA | NA | Discovery |
| CRISPLD2 | Weighted median | 5 | 1.0009 | 1.0001 | 1.0017 | 2.35E-02 | 0.0173 | 0.3263 | Discovery |
| CRYGD | Inverse variance weighted | 2 | 1.0030 | 1.0018 | 1.0041 | 7.91E-07 | NA | NA | Discovery |
| CSF1R | Inverse variance weighted | 3 | 0.9992 | 0.9990 | 0.9993 | 5.99E-40 | NA | NA | Discovery |
| CST3 | Wald ratio | 1 | 0.9992 | 0.9984 | 1.0000 | 4.55E-02 | NA | NA | Discovery |
| CST4 | Inverse variance weighted | 3 | 1.0003 | 1.0000 | 1.0006 | 3.50E-02 | NA | NA | Discovery |
| CST5 | Inverse variance weighted | 2 | 0.9993 | 0.9990 | 0.9997 | 1.72E-04 | NA | NA | Discovery |
| CST7 | Inverse variance weighted | 2 | 0.9995 | 0.9993 | 0.9996 | 3.37E-14 | NA | NA | Discovery |
| CTSD | Inverse variance weighted | 3 | 0.9990 | 0.9983 | 0.9997 | 7.87E-03 | NA | NA | Discovery |
| CTSO | Inverse variance weighted | 4 | 1.0005 | 1.0003 | 1.0008 | 6.65E-05 | 0.9567 | 0.7139 | Discovery |
| CTSV | Inverse variance weighted | 3 | 0.9990 | 0.9982 | 0.9998 | 1.48E-02 | NA | NA | Discovery |
| CYB5R2 | Inverse variance weighted | 6 | 1.0009 | 1.0004 | 1.0014 | 1.11E-04 | 0.7372 | 0.5734 | Discovery |
| CYB5R2 | Weighted median | 6 | 1.0008 | 1.0001 | 1.0016 | 1.82E-02 | 0.7372 | 0.5734 | Discovery |
| DARS2 | Inverse variance weighted | 2 | 0.9983 | 0.9983 | 0.9984 | 0.00E+00 | NA | NA | Discovery |
| DBNL | Wald ratio | 1 | 1.0029 | 1.0001 | 1.0057 | 4.55E-02 | NA | NA | Discovery |
| DCLK1 | Inverse variance weighted | 2 | 0.9995 | 0.9993 | 0.9997 | 1.25E-07 | NA | NA | Discovery |
| DCTPP1 | Wald ratio | 1 | 1.0033 | 1.0001 | 1.0066 | 4.55E-02 | NA | NA | Discovery |
| DLK1 | Inverse variance weighted | 4 | 0.9997 | 0.9995 | 0.9999 | 5.52E-03 | 0.9054 | 0.5500 | Discovery |
| DLK2 | Wald ratio | 1 | 0.9989 | 0.9978 | 1.0000 | 4.55E-02 | NA | NA | Discovery |
| EDAR | Inverse variance weighted | 3 | 1.0003 | 1.0003 | 1.0004 | 3.28E-59 | NA | NA | Discovery |
| EFNB2 | Inverse variance weighted | 2 | 0.9985 | 0.9980 | 0.9991 | 3.80E-08 | NA | NA | Discovery |
| EGFR | Inverse variance weighted | 2 | 0.9975 | 0.9961 | 0.9990 | 7.69E-04 | NA | NA | Discovery |
| ENPEP | Inverse variance weighted | 5 | 0.9995 | 0.9992 | 0.9999 | 4.12E-03 | 0.6826 | 0.7945 | Discovery |
| ENPP2 | Wald ratio | 1 | 0.9987 | 0.9974 | 1.0000 | 4.55E-02 | NA | NA | Discovery |
| ENPP6 | Inverse variance weighted | 2 | 0.9976 | 0.9967 | 0.9985 | 2.52E-07 | NA | NA | Discovery |
| ENTPD5 | Wald ratio | 1 | 1.0015 | 1.0000 | 1.0031 | 4.55E-02 | NA | NA | Discovery |
| EPHB1 | Inverse variance weighted | 4 | 1.0007 | 1.0003 | 1.0012 | 1.89E-03 | 0.3793 | 0.4969 | Discovery |
| EPHB1 | Weighted median | 4 | 1.0008 | 1.0003 | 1.0013 | 3.57E-03 | 0.3793 | 0.4969 | Discovery |
| EPHB4 | Wald ratio | 1 | 0.9982 | 0.9964 | 1.0000 | 4.55E-02 | NA | NA | Discovery |
| ESD | Inverse variance weighted | 2 | 1.0004 | 1.0001 | 1.0007 | 5.96E-03 | NA | NA | Discovery |
| EVL | Inverse variance weighted | 2 | 0.9997 | 0.9995 | 0.9999 | 2.89E-04 | NA | NA | Discovery |
| FAM151A | Inverse variance weighted | 2 | 1.0004 | 1.0001 | 1.0006 | 2.70E-03 | NA | NA | Discovery |
| FAM177A1 | Inverse variance weighted | 2 | 1.0010 | 1.0003 | 1.0016 | 4.43E-03 | NA | NA | Discovery |
| FAM49B | Wald ratio | 1 | 0.9960 | 0.9921 | 0.9999 | 4.55E-02 | NA | NA | Discovery |
| FAS | Inverse variance weighted | 3 | 1.0008 | 1.0007 | 1.0009 | 4.19E-121 | NA | NA | Discovery |
| FAS | Weighted median | 3 | 1.0008 | 1.0001 | 1.0015 | 1.78E-02 | NA | NA | Discovery |
| FBLN1 | Inverse variance weighted | 2 | 0.9995 | 0.9991 | 0.9999 | 1.01E-02 | NA | NA | Discovery |
| FBLN5 | Inverse variance weighted | 2 | 0.9990 | 0.9980 | 1.0000 | 4.55E-02 | NA | NA | Discovery |
| FCER2 | Inverse variance weighted | 7 | 0.9997 | 0.9993 | 1.0000 | 4.72E-02 | 0.5257 | 0.4206 | Discovery |
| FCN1 | Weighted median | 5 | 1.0005 | 1.0000 | 1.0010 | 4.64E-02 | 0.1362 | 0.1946 | Discovery |
| FCRL6 | Wald ratio | 1 | 1.0013 | 1.0000 | 1.0026 | 4.55E-02 | NA | NA | Discovery |
| FGFR1 | Wald ratio | 1 | 0.9971 | 0.9944 | 0.9999 | 4.55E-02 | NA | NA | Discovery |
| FGR | Inverse variance weighted | 3 | 0.9989 | 0.9982 | 0.9996 | 1.57E-03 | NA | NA | Discovery |
| FIGF | Inverse variance weighted | 6 | 0.9993 | 0.9990 | 0.9997 | 5.76E-04 | 0.9330 | 0.8593 | Discovery |
| FJX1 | Inverse variance weighted | 5 | 1.0009 | 1.0001 | 1.0016 | 1.98E-02 | 0.6179 | 0.4731 | Discovery |
| FKBP1B | Inverse variance weighted | 2 | 0.9995 | 0.9991 | 1.0000 | 3.95E-02 | NA | NA | Discovery |
| FLRT3 | Inverse variance weighted | 4 | 1.0011 | 1.0006 | 1.0017 | 9.70E-05 | 0.7645 | 0.7003 | Discovery |
| FLRT3 | Weighted median | 4 | 1.0010 | 1.0000 | 1.0020 | 4.75E-02 | 0.7645 | 0.7003 | Discovery |
| FN1 | Wald ratio | 1 | 1.0019 | 1.0007 | 1.0031 | 2.70E-03 | NA | NA | Discovery |
| FSTL1 | Inverse variance weighted | 2 | 0.9992 | 0.9989 | 0.9995 | 5.73E-07 | NA | NA | Discovery |
| GAA | Weighted median | 3 | 0.9994 | 0.9989 | 1.0000 | 3.26E-02 | NA | NA | Discovery |
| GAS6 | Inverse variance weighted | 3 | 0.9997 | 0.9995 | 0.9998 | 2.36E-04 | NA | NA | Discovery |
| GFRA2 | Weighted median | 6 | 1.0005 | 1.0000 | 1.0010 | 4.19E-02 | 0.2307 | 0.7424 | Discovery |
| GIF | Inverse variance weighted | 2 | 0.9995 | 0.9994 | 0.9996 | 2.56E-12 | NA | NA | Discovery |
| GLB1 | Wald ratio | 1 | 1.0040 | 1.0001 | 1.0080 | 4.55E-02 | NA | NA | Discovery |
| GLCE | Inverse variance weighted | 2 | 1.0008 | 1.0006 | 1.0009 | 4.15E-26 | NA | NA | Discovery |
| GLIPR2 | Inverse variance weighted | 4 | 0.9992 | 0.9988 | 0.9997 | 4.39E-04 | 0.4644 | 0.4724 | Discovery |
| GLIPR2 | Weighted median | 4 | 0.9992 | 0.9986 | 0.9997 | 1.49E-03 | 0.4644 | 0.4724 | Discovery |
| GLRX2 | Inverse variance weighted | 2 | 1.0004 | 1.0003 | 1.0005 | 6.29E-32 | NA | NA | Discovery |
| GSTP1 | Inverse variance weighted | 2 | 0.9981 | 0.9965 | 0.9998 | 2.44E-02 | NA | NA | Discovery |
| H6PD | Inverse variance weighted | 2 | 1.0003 | 1.0002 | 1.0004 | 2.70E-06 | NA | NA | Discovery |
| HAVCR1 | Wald ratio | 1 | 1.0012 | 1.0004 | 1.0019 | 2.70E-03 | NA | NA | Discovery |
| HBZ | Inverse variance weighted | 3 | 1.0003 | 1.0001 | 1.0005 | 1.58E-02 | NA | NA | Discovery |
| HEXB | Inverse variance weighted | 2 | 0.9995 | 0.9992 | 0.9999 | 5.82E-03 | NA | NA | Discovery |
| HK2 | Inverse variance weighted | 3 | 0.9950 | 0.9946 | 0.9954 | 4.11E-139 | NA | NA | Discovery |
| HK2 | Weighted median | 3 | 0.9950 | 0.9920 | 0.9980 | 1.22E-03 | NA | NA | Discovery |
| HLA-DQA2 | Inverse variance weighted | 3 | 1.0008 | 1.0001 | 1.0014 | 1.89E-02 | NA | NA | Discovery |
| HLA-DQA2 | Weighted median | 3 | 1.0008 | 1.0000 | 1.0016 | 4.15E-02 | NA | NA | Discovery |
| HS6ST1 | Inverse variance weighted | 3 | 1.0008 | 1.0003 | 1.0012 | 1.50E-03 | NA | NA | Discovery |
| HSD17B14 | Inverse variance weighted | 3 | 0.9977 | 0.9966 | 0.9989 | 8.56E-05 | NA | NA | Discovery |
| HSPA1B | Wald ratio | 1 | 0.9950 | 0.9918 | 0.9983 | 2.70E-03 | NA | NA | Discovery |
| HTRA1 | Inverse variance weighted | 2 | 0.9989 | 0.9978 | 0.9999 | 3.42E-02 | NA | NA | Discovery |
| ICAM4 | Wald ratio | 1 | 0.9982 | 0.9964 | 1.0000 | 4.55E-02 | NA | NA | Discovery |
| IFI16 | Inverse variance weighted | 2 | 1.0023 | 1.0019 | 1.0027 | 5.93E-27 | NA | NA | Discovery |
| IGDCC4 | Weighted median | 5 | 0.9989 | 0.9981 | 0.9997 | 8.31E-03 | 0.0804 | 0.9447 | Discovery |
| IGF1R | Inverse variance weighted | 2 | 0.9977 | 0.9975 | 0.9979 | 2.88E-107 | NA | NA | Discovery |
| IGFBP2 | Wald ratio | 1 | 0.2133 | 0.0561 | 0.8114 | 2.34E-02 | NA | NA | Discovery |
| IGLL1 | Inverse variance weighted | 3 | 1.0004 | 1.0002 | 1.0006 | 9.42E-04 | NA | NA | Discovery |
| IL13RA1 | Wald ratio | 1 | 1.0007 | 1.0000 | 1.0014 | 4.55E-02 | NA | NA | Discovery |
| IL16 | Wald ratio | 1 | 0.9980 | 0.9960 | 1.0000 | 4.55E-02 | NA | NA | Discovery |
| IL17B | Wald ratio | 1 | 1.0029 | 1.0001 | 1.0057 | 4.55E-02 | NA | NA | Discovery |
| IL18RAP | Wald ratio | 1 | 1.0025 | 1.0001 | 1.0050 | 4.55E-02 | NA | NA | Discovery |
| IL1RL2 | Inverse variance weighted | 3 | 0.9996 | 0.9992 | 0.9999 | 9.37E-03 | NA | NA | Discovery |
| IL20RA | Wald ratio | 1 | 1.0033 | 1.0001 | 1.0066 | 4.55E-02 | NA | NA | Discovery |
| IL27RA | Wald ratio | 1 | 0.9986 | 0.9972 | 1.0000 | 4.55E-02 | NA | NA | Discovery |
| IRF3 | Wald ratio | 1 | 1.0029 | 1.0001 | 1.0057 | 4.55E-02 | NA | NA | Discovery |
| ISOC1 | Inverse variance weighted | 2 | 0.9982 | 0.9979 | 0.9985 | 3.82E-28 | NA | NA | Discovery |
| ITPKA | Wald ratio | 1 | 0.9971 | 0.9944 | 0.9999 | 4.55E-02 | NA | NA | Discovery |
| KIAA1161 | Wald ratio | 1 | 0.9983 | 0.9967 | 1.0000 | 4.55E-02 | NA | NA | Discovery |
| KIAA1549L | Wald ratio | 1 | 1.0010 | 1.0000 | 1.0020 | 4.55E-02 | NA | NA | Discovery |
| KIR2DL4 | Inverse variance weighted | 3 | 0.8468 | 0.7601 | 0.9434 | 2.55E-03 | NA | NA | Discovery |
| KLK13 | Inverse variance weighted | 4 | 0.9989 | 0.9979 | 0.9999 | 2.73E-02 | 0.3244 | 0.3190 | Discovery |
| KLK13 | Weighted median | 4 | 0.9987 | 0.9976 | 0.9998 | 2.03E-02 | 0.3244 | 0.3190 | Discovery |
| KLK7 | Inverse variance weighted | 6 | 1.0005 | 1.0003 | 1.0007 | 6.84E-06 | 0.9728 | 0.9578 | Discovery |
| KLKB1 | Inverse variance weighted | 2 | 1.0004 | 1.0000 | 1.0008 | 4.55E-02 | NA | NA | Discovery |
| KLRB1 | Wald ratio | 1 | 0.9987 | 0.9978 | 0.9995 | 2.70E-03 | NA | NA | Discovery |
| LEFTY2 | Weighted median | 5 | 1.0011 | 1.0002 | 1.0020 | 1.66E-02 | 0.3404 | 0.5083 | Discovery |
| LEPR | Wald ratio | 1 | 1.0008 | 1.0000 | 1.0016 | 4.55E-02 | NA | NA | Discovery |
| LGALS2 | Wald ratio | 1 | 1.0010 | 1.0000 | 1.0019 | 4.55E-02 | NA | NA | Discovery |
| LGMN | Weighted median | 3 | 1.0015 | 1.0001 | 1.0030 | 4.09E-02 | NA | NA | Discovery |
| LILRA3 | Inverse variance weighted | 6 | 0.9996 | 0.9992 | 1.0000 | 4.21E-02 | 0.3174 | 0.1345 | Discovery |
| LILRA3 | Weighted median | 6 | 0.9996 | 0.9991 | 1.0000 | 4.03E-02 | 0.3174 | 0.1345 | Discovery |
| LILRA4 | Inverse variance weighted | 5 | 0.9994 | 0.9989 | 1.0000 | 4.72E-02 | 0.3392 | 0.2075 | Discovery |
| LILRA4 | Weighted median | 5 | 0.9993 | 0.9988 | 0.9999 | 2.04E-02 | 0.3392 | 0.2075 | Discovery |
| LILRB3 | Inverse variance weighted | 6 | 0.9994 | 0.9988 | 0.9999 | 3.23E-02 | 0.2955 | 0.3900 | Discovery |
| LOXL3 | Inverse variance weighted | 2 | 1.0014 | 1.0002 | 1.0026 | 2.25E-02 | NA | NA | Discovery |
| LPO | Inverse variance weighted | 2 | 1.0003 | 1.0003 | 1.0004 | 2.01E-77 | NA | NA | Discovery |
| LRIG1 | Inverse variance weighted | 2 | 1.0012 | 1.0009 | 1.0015 | 1.24E-15 | NA | NA | Discovery |
| LRRC32 | Wald ratio | 1 | 1.0029 | 1.0001 | 1.0057 | 4.55E-02 | NA | NA | Discovery |
| LRRC4B | Inverse variance weighted | 2 | 0.3722 | 0.1865 | 0.7430 | 5.07E-03 | NA | NA | Discovery |
| LY9 | Wald ratio | 1 | 1.0025 | 1.0001 | 1.0050 | 4.55E-02 | NA | NA | Discovery |
| MAPK8 | Wald ratio | 1 | 0.9967 | 0.9934 | 0.9999 | 4.55E-02 | NA | NA | Discovery |
| MAPKAPK2 | Inverse variance weighted | 2 | 1.0006 | 1.0006 | 1.0006 | 0.00E+00 | NA | NA | Discovery |
| MARK3 | Wald ratio | 1 | 0.9967 | 0.9934 | 0.9999 | 4.55E-02 | NA | NA | Discovery |
| MATN2 | Inverse variance weighted | 4 | 0.9984 | 0.9974 | 0.9993 | 9.55E-04 | 0.4845 | 0.8448 | Discovery |
| MATN2 | Weighted median | 4 | 0.9982 | 0.9970 | 0.9995 | 5.68E-03 | 0.4845 | 0.8448 | Discovery |
| MDK | Inverse variance weighted | 2 | 0.9987 | 0.9981 | 0.9993 | 1.60E-05 | NA | NA | Discovery |
| METTL24 | Inverse variance weighted | 2 | 0.9986 | 0.9984 | 0.9989 | 4.18E-22 | NA | NA | Discovery |
| MGAT2 | Wald ratio | 1 | 1.0015 | 1.0000 | 1.0031 | 4.55E-02 | NA | NA | Discovery |
| MGP | Wald ratio | 1 | 0.9992 | 0.9984 | 1.0000 | 4.55E-02 | NA | NA | Discovery |
| MIA | Wald ratio | 1 | 1.0008 | 1.0000 | 1.0015 | 4.55E-02 | NA | NA | Discovery |
| MPG | Wald ratio | 1 | 0.4293 | 0.1974 | 0.9336 | 3.29E-02 | NA | NA | Discovery |
| MRC1 | Inverse variance weighted | 2 | 0.8296 | 0.7359 | 0.9352 | 2.25E-03 | NA | NA | Discovery |
| MSMB | Weighted median | 3 | 0.9991 | 0.9984 | 0.9997 | 6.66E-03 | NA | NA | Discovery |
| MTHFS | Inverse variance weighted | 3 | 0.9997 | 0.9995 | 0.9999 | 3.30E-03 | NA | NA | Discovery |
| MTHFSD | Inverse variance weighted | 2 | 0.9989 | 0.9984 | 0.9993 | 1.34E-07 | NA | NA | Discovery |
| NADK | Inverse variance weighted | 2 | 1.0010 | 1.0005 | 1.0015 | 1.45E-04 | NA | NA | Discovery |
| NAGPA | Inverse variance weighted | 7 | 1.0005 | 1.0002 | 1.0008 | 2.93E-03 | 0.8019 | 0.8767 | Discovery |
| NAP1L4 | Wald ratio | 1 | 0.9989 | 0.9978 | 1.0000 | 4.55E-02 | NA | NA | Discovery |
| NBL1 | Wald ratio | 1 | 0.9960 | 0.9921 | 0.9999 | 4.55E-02 | NA | NA | Discovery |
| NCAM1 | Inverse variance weighted | 4 | 0.9994 | 0.9991 | 0.9998 | 1.82E-03 | 0.7581 | 0.4517 | Discovery |
| NCAM1 | Weighted median | 4 | 0.9993 | 0.9986 | 1.0000 | 4.42E-02 | 0.7581 | 0.4517 | Discovery |
| NCAM2 | Inverse variance weighted | 8 | 0.9994 | 0.9988 | 0.9999 | 3.23E-02 | 0.2829 | 0.1178 | Discovery |
| NCR3 | Inverse variance weighted | 3 | 0.9988 | 0.9978 | 0.9998 | 2.10E-02 | NA | NA | Discovery |
| NDST1 | Inverse variance weighted | 3 | 1.0006 | 1.0002 | 1.0010 | 7.08E-03 | NA | NA | Discovery |
| NID1 | Inverse variance weighted | 2 | 1.0019 | 1.0019 | 1.0019 | 0.00E+00 | NA | NA | Discovery |
| NID2 | Inverse variance weighted | 3 | 1.0004 | 1.0001 | 1.0007 | 5.31E-03 | NA | NA | Discovery |
| NLGN1 | Inverse variance weighted | 4 | 1.0014 | 1.0006 | 1.0021 | 2.15E-04 | 0.7132 | 0.6400 | Discovery |
| NLGN1 | Weighted median | 4 | 1.0015 | 1.0002 | 1.0027 | 2.72E-02 | 0.7132 | 0.6400 | Discovery |
| NLGN4X | Wald ratio | 1 | 0.9967 | 0.9934 | 0.9999 | 4.55E-02 | NA | NA | Discovery |
| NMB | Inverse variance weighted | 2 | 1.0007 | 1.0007 | 1.0007 | 0.00E+00 | NA | NA | Discovery |
| NOG | Inverse variance weighted | 3 | 0.9997 | 0.9994 | 0.9999 | 1.89E-02 | NA | NA | Discovery |
| NPPB | Inverse variance weighted | 2 | 1.0011 | 1.0010 | 1.0011 | 0.00E+00 | NA | NA | Discovery |
| NPTXR | Inverse variance weighted | 2 | 1.0008 | 1.0005 | 1.0012 | 4.22E-06 | NA | NA | Discovery |
| NRK1 | Inverse variance weighted | 2 | 0.9992 | 0.9986 | 0.9997 | 4.61E-03 | NA | NA | Discovery |
| NRP1 | Inverse variance weighted | 4 | 1.0009 | 1.0003 | 1.0014 | 1.88E-03 | 0.2862 | 0.7782 | Discovery |
| NRP1 | Weighted median | 4 | 1.0009 | 1.0004 | 1.0014 | 5.95E-04 | 0.2862 | 0.7782 | Discovery |
| NT5E | Inverse variance weighted | 3 | 0.9991 | 0.9987 | 0.9994 | 1.08E-08 | NA | NA | Discovery |
| NT5E | Weighted median | 3 | 0.9991 | 0.9985 | 0.9997 | 2.96E-03 | NA | NA | Discovery |
| NTRK3 | Inverse variance weighted | 4 | 1.0012 | 1.0002 | 1.0022 | 1.92E-02 | 0.2364 | 0.1916 | Discovery |
| NTRK3 | Weighted median | 4 | 1.0012 | 1.0001 | 1.0022 | 2.96E-02 | 0.2364 | 0.1916 | Discovery |
| NUDT2 | Inverse variance weighted | 2 | 0.9992 | 0.9984 | 1.0000 | 4.55E-02 | NA | NA | Discovery |
| OLFM1 | Inverse variance weighted | 3 | 1.0007 | 1.0001 | 1.0012 | 2.47E-02 | NA | NA | Discovery |
| OLFML3 | Wald ratio | 1 | 1.0033 | 1.0012 | 1.0055 | 2.70E-03 | NA | NA | Discovery |
| PCDH10 | Inverse variance weighted | 2 | 0.9993 | 0.9988 | 0.9997 | 2.70E-03 | NA | NA | Discovery |
| PCOLCE2 | Inverse variance weighted | 7 | 1.0003 | 1.0000 | 1.0006 | 3.71E-02 | 0.7700 | 0.9480 | Discovery |
| PCSK9 | Inverse variance weighted | 3 | 1.0010 | 1.0002 | 1.0018 | 9.40E-03 | NA | NA | Discovery |
| PDCD5 | Wald ratio | 1 | 0.9994 | 0.9987 | 1.0000 | 4.55E-02 | NA | NA | Discovery |
| PFKM | Inverse variance weighted | 2 | 1.0007 | 1.0004 | 1.0010 | 2.14E-05 | NA | NA | Discovery |
| PIGR | Inverse variance weighted | 2 | 0.9994 | 0.9989 | 0.9998 | 5.11E-03 | NA | NA | Discovery |
| PIR | Inverse variance weighted | 3 | 0.9994 | 0.9989 | 0.9998 | 4.43E-03 | NA | NA | Discovery |
| PLG | Inverse variance weighted | 5 | 1.0013 | 1.0001 | 1.0026 | 3.46E-02 | 0.4712 | 0.5329 | Discovery |
| PLXNA1 | Wald ratio | 1 | 0.9992 | 0.9984 | 1.0000 | 4.55E-02 | NA | NA | Discovery |
| PNKP | Wald ratio | 1 | 1.0020 | 1.0000 | 1.0040 | 4.55E-02 | NA | NA | Discovery |
| PNP | Inverse variance weighted | 3 | 0.9991 | 0.9986 | 0.9996 | 1.27E-04 | NA | NA | Discovery |
| PRCP | Inverse variance weighted | 2 | 1.0017 | 1.0003 | 1.0032 | 1.97E-02 | NA | NA | Discovery |
| PRSS1 | Wald ratio | 1 | 6.2599 | 1.0657 | 36.7707 | 4.23E-02 | NA | NA | Discovery |
| PRSS27 | Inverse variance weighted | 2 | 0.7442 | 0.5810 | 0.9532 | 1.93E-02 | NA | NA | Discovery |
| PRSS57 | Wald ratio | 1 | 0.9967 | 0.9934 | 0.9999 | 4.55E-02 | NA | NA | Discovery |
| PSAT1 | Wald ratio | 1 | 1.0013 | 1.0000 | 1.0026 | 4.55E-02 | NA | NA | Discovery |
| PTGR1 | Inverse variance weighted | 5 | 0.9994 | 0.9990 | 0.9998 | 3.29E-03 | 0.8537 | 0.4236 | Discovery |
| PXDN | Inverse variance weighted | 2 | 0.9986 | 0.9984 | 0.9988 | 3.55E-33 | NA | NA | Discovery |
| QPCTL | Wald ratio | 1 | 1.0029 | 1.0015 | 1.0043 | 6.33E-05 | NA | NA | Discovery |
| RAB31 | Wald ratio | 1 | 0.9985 | 0.9970 | 1.0000 | 4.55E-02 | NA | NA | Discovery |
| REG3G | Inverse variance weighted | 2 | 0.9987 | 0.9976 | 0.9998 | 1.81E-02 | NA | NA | Discovery |
| RELT | Wald ratio | 1 | 1.0033 | 1.0001 | 1.0066 | 4.55E-02 | NA | NA | Discovery |
| RNASE1 | Inverse variance weighted | 3 | 0.9995 | 0.9994 | 0.9996 | 3.50E-17 | NA | NA | Discovery |
| RNASE2 | Wald ratio | 1 | 0.9986 | 0.9972 | 1.0000 | 4.55E-02 | NA | NA | Discovery |
| ROR1 | Inverse variance weighted | 6 | 0.9991 | 0.9987 | 0.9996 | 1.50E-04 | 0.5219 | 0.5780 | Discovery |
| ROR1 | Weighted median | 6 | 0.9990 | 0.9983 | 0.9996 | 1.52E-03 | 0.5219 | 0.5780 | Discovery |
| ROR2 | Weighted median | 3 | 0.9989 | 0.9979 | 1.0000 | 4.66E-02 | NA | NA | Discovery |
| RRM1 | Wald ratio | 1 | 1.0033 | 1.0001 | 1.0066 | 4.55E-02 | NA | NA | Discovery |
| S100A12 | Inverse variance weighted | 2 | 1.0004 | 1.0001 | 1.0007 | 9.65E-03 | NA | NA | Discovery |
| S100A14 | Wald ratio | 1 | 1.0022 | 1.0000 | 1.0044 | 4.55E-02 | NA | NA | Discovery |
| S100A16 | Inverse variance weighted | 2 | 0.9989 | 0.9987 | 0.9991 | 2.26E-19 | NA | NA | Discovery |
| S100A6 | Weighted median | 3 | 0.9978 | 0.9958 | 0.9998 | 3.20E-02 | NA | NA | Discovery |
| SAR1A | Wald ratio | 1 | 1.0013 | 1.0000 | 1.0026 | 4.55E-02 | NA | NA | Discovery |
| SCARA5 | Inverse variance weighted | 3 | 1.0004 | 1.0003 | 1.0006 | 7.96E-09 | NA | NA | Discovery |
| SCARF1 | Inverse variance weighted | 3 | 0.9990 | 0.9984 | 0.9996 | 6.82E-04 | NA | NA | Discovery |
| SCARF2 | Inverse variance weighted | 2 | 0.9974 | 0.9970 | 0.9978 | 1.07E-37 | NA | NA | Discovery |
| SCUBE1 | Inverse variance weighted | 3 | 0.9968 | 0.9955 | 0.9981 | 1.36E-06 | NA | NA | Discovery |
| SCUBE1 | Weighted median | 3 | 0.9968 | 0.9955 | 0.9982 | 9.31E-06 | NA | NA | Discovery |
| SCUBE3 | Inverse variance weighted | 2 | 0.9997 | 0.9994 | 0.9999 | 6.93E-03 | NA | NA | Discovery |
| SELL | Inverse variance weighted | 2 | 1.0011 | 1.0009 | 1.0012 | 7.06E-32 | NA | NA | Discovery |
| SELPLG | Inverse variance weighted | 3 | 1.0008 | 1.0003 | 1.0013 | 1.82E-03 | NA | NA | Discovery |
| SELPLG | Weighted median | 3 | 1.0008 | 1.0001 | 1.0015 | 1.86E-02 | NA | NA | Discovery |
| SEMA4A | Inverse variance weighted | 2 | 1.0012 | 1.0007 | 1.0018 | 2.42E-05 | NA | NA | Discovery |
| SEMA5A | Inverse variance weighted | 2 | 0.9996 | 0.9993 | 0.9998 | 9.59E-04 | NA | NA | Discovery |
| SEMA6A | Inverse variance weighted | 2 | 0.9988 | 0.9978 | 0.9998 | 1.96E-02 | NA | NA | Discovery |
| SEMA6B | Wald ratio | 1 | 1.0022 | 1.0000 | 1.0044 | 4.55E-02 | NA | NA | Discovery |
| SERPINA1 | Inverse variance weighted | 2 | 0.9994 | 0.9993 | 0.9996 | 2.56E-12 | NA | NA | Discovery |
| SERPINA7 | Wald ratio | 1 | 1.0007 | 1.0000 | 1.0013 | 4.55E-02 | NA | NA | Discovery |
| SERPINA9 | Inverse variance weighted | 3 | 0.9995 | 0.9991 | 0.9999 | 8.07E-03 | NA | NA | Discovery |
| SERPINB13 | Wald ratio | 1 | 1.0014 | 1.0000 | 1.0027 | 4.55E-02 | NA | NA | Discovery |
| SERPINF1 | Inverse variance weighted | 4 | 0.9995 | 0.9992 | 0.9998 | 2.99E-03 | 0.7267 | 0.7633 | Discovery |
| SF3B4 | Wald ratio | 1 | 1.0045 | 1.0001 | 1.0088 | 4.55E-02 | NA | NA | Discovery |
| SFTPB | Inverse variance weighted | 2 | 1.0008 | 1.0005 | 1.0011 | 8.80E-07 | NA | NA | Discovery |
| SGK3 | Wald ratio | 1 | 1.0048 | 1.0005 | 1.0091 | 2.78E-02 | NA | NA | Discovery |
| SHBG | Inverse variance weighted | 3 | 1.0012 | 1.0004 | 1.0021 | 3.14E-03 | NA | NA | Discovery |
| SHBG | Weighted median | 3 | 1.0014 | 1.0006 | 1.0022 | 7.59E-04 | NA | NA | Discovery |
| SHH | Inverse variance weighted | 2 | 1.0011 | 1.0009 | 1.0012 | 7.06E-32 | NA | NA | Discovery |
| SHMT1 | Inverse variance weighted | 2 | 1.0317 | 1.0190 | 1.0445 | 7.58E-07 | NA | NA | Discovery |
| SIGLEC14 | Inverse variance weighted | 4 | 0.9995 | 0.9993 | 0.9997 | 1.27E-07 | 0.9364 | 0.7770 | Discovery |
| SIGLEC14 | Weighted median | 4 | 0.9994 | 0.9989 | 1.0000 | 3.37E-02 | 0.9364 | 0.7770 | Discovery |
| SLAMF6 | Wald ratio | 1 | 0.9981 | 0.9972 | 0.9990 | 6.33E-05 | NA | NA | Discovery |
| SMAP1 | Wald ratio | 1 | 1.0009 | 1.0003 | 1.0016 | 2.70E-03 | NA | NA | Discovery |
| SMOC2 | Inverse variance weighted | 5 | 1.0002 | 1.0000 | 1.0003 | 3.30E-02 | 0.9647 | 0.5453 | Discovery |
| SNUPN | Inverse variance weighted | 2 | 1.0015 | 1.0003 | 1.0027 | 1.57E-02 | NA | NA | Discovery |
| SPINK2 | Inverse variance weighted | 4 | 1.0007 | 1.0001 | 1.0014 | 3.52E-02 | 0.2771 | 0.6249 | Discovery |
| SPINK2 | Weighted median | 4 | 1.0010 | 1.0002 | 1.0018 | 1.90E-02 | 0.2771 | 0.6249 | Discovery |
| SRA1 | Inverse variance weighted | 2 | 0.9987 | 0.9978 | 0.9997 | 8.91E-03 | NA | NA | Discovery |
| SRI | Wald ratio | 1 | 0.9986 | 0.9972 | 1.0000 | 4.55E-02 | NA | NA | Discovery |
| ST3GAL1 | Inverse variance weighted | 3 | 1.0007 | 1.0003 | 1.0012 | 1.29E-03 | NA | NA | Discovery |
| ST3GAL1 | Weighted median | 3 | 1.0007 | 1.0000 | 1.0015 | 4.73E-02 | NA | NA | Discovery |
| TAC1 | Inverse variance weighted | 4 | 0.9989 | 0.9980 | 0.9998 | 2.02E-02 | 0.6129 | 0.8497 | Discovery |
| TBCE | Inverse variance weighted | 2 | 0.9992 | 0.9984 | 0.9999 | 2.63E-02 | NA | NA | Discovery |
| TEK | Inverse variance weighted | 2 | 1.0002 | 1.0001 | 1.0003 | 1.31E-09 | NA | NA | Discovery |
| TFPI2 | Wald ratio | 1 | 0.9964 | 0.9928 | 0.9999 | 4.55E-02 | NA | NA | Discovery |
| TGFBI | Inverse variance weighted | 3 | 0.9965 | 0.9931 | 0.9998 | 3.86E-02 | NA | NA | Discovery |
| TIGIT | Wald ratio | 1 | 1.0025 | 1.0001 | 1.0050 | 4.55E-02 | NA | NA | Discovery |
| TIMP2 | Inverse variance weighted | 2 | 0.9986 | 0.9982 | 0.9990 | 2.56E-12 | NA | NA | Discovery |
| TMEM132B | Inverse variance weighted | 2 | 0.9995 | 0.9991 | 1.0000 | 3.36E-02 | NA | NA | Discovery |
| TMEM132C | Inverse variance weighted | 3 | 0.9992 | 0.9985 | 0.9998 | 6.54E-03 | NA | NA | Discovery |
| TMEM132C | Weighted median | 3 | 0.9993 | 0.9986 | 0.9999 | 2.58E-02 | NA | NA | Discovery |
| TMEM132D | Inverse variance weighted | 3 | 0.9987 | 0.9980 | 0.9994 | 2.55E-04 | NA | NA | Discovery |
| TMEM132D | Weighted median | 3 | 0.9987 | 0.9974 | 0.9999 | 3.79E-02 | NA | NA | Discovery |
| TNC | Inverse variance weighted | 3 | 1.0011 | 1.0008 | 1.0013 | 3.74E-19 | NA | NA | Discovery |
| TNC | Weighted median | 3 | 1.0011 | 1.0004 | 1.0018 | 1.63E-03 | NA | NA | Discovery |
| TNFAIP8 | Inverse variance weighted | 2 | 0.9981 | 0.9972 | 0.9990 | 3.94E-05 | NA | NA | Discovery |
| TNFRSF1A | Inverse variance weighted | 2 | 1.0013 | 1.0012 | 1.0015 | 5.37E-83 | NA | NA | Discovery |
| TNFSF8 | Wald ratio | 1 | 1.0021 | 1.0007 | 1.0035 | 2.70E-03 | NA | NA | Discovery |
| TNR | Wald ratio | 1 | 1.0015 | 1.0000 | 1.0031 | 4.55E-02 | NA | NA | Discovery |
| TNXB | Inverse variance weighted | 3 | 0.9989 | 0.9988 | 0.9990 | 4.53E-58 | NA | NA | Discovery |
| TNXB | Weighted median | 3 | 0.9989 | 0.9983 | 0.9995 | 3.88E-04 | NA | NA | Discovery |
| TPI1 | Wald ratio | 1 | 0.9934 | 0.9901 | 0.9966 | 6.33E-05 | NA | NA | Discovery |
| TWSG1 | Inverse variance weighted | 2 | 1.0023 | 1.0020 | 1.0026 | 1.05E-48 | NA | NA | Discovery |
| TYRO3 | Wald ratio | 1 | 0.9982 | 0.9964 | 1.0000 | 4.55E-02 | NA | NA | Discovery |
| UBXN2B | Wald ratio | 1 | 1.0033 | 1.0001 | 1.0066 | 4.55E-02 | NA | NA | Discovery |
| UGT1A6 | Inverse variance weighted | 2 | 1.0008 | 1.0003 | 1.0013 | 1.02E-03 | NA | NA | Discovery |
| UNC5D | Wald ratio | 1 | 1.0020 | 1.0000 | 1.0040 | 4.55E-02 | NA | NA | Discovery |
| UROS | Inverse variance weighted | 2 | 0.9991 | 0.9990 | 0.9992 | 6.41E-43 | NA | NA | Discovery |
| UST | Inverse variance weighted | 4 | 0.9986 | 0.9981 | 0.9991 | 2.82E-07 | 0.9025 | 0.8077 | Discovery |
| UST | Weighted median | 4 | 0.9986 | 0.9973 | 0.9999 | 3.64E-02 | 0.9025 | 0.8077 | Discovery |
| VEGFA | Inverse variance weighted | 5 | 1.0008 | 1.0002 | 1.0014 | 1.01E-02 | 0.8385 | 0.9267 | Discovery |
| VNN2 | Inverse variance weighted | 2 | 1.0012 | 1.0011 | 1.0013 | 8.88E-139 | NA | NA | Discovery |
| VPS26A | Wald ratio | 1 | 0.9989 | 0.9978 | 1.0000 | 4.55E-02 | NA | NA | Discovery |
| VWF | Inverse variance weighted | 2 | 0.9983 | 0.9981 | 0.9985 | 1.95E-72 | NA | NA | Discovery |
| ZG16B | Wald ratio | 1 | 1.0011 | 1.0000 | 1.0022 | 4.55E-02 | NA | NA | Discovery |
| ADAM23 | Wald ratio | 1 | 1.0005 | 1.0000 | 1.0009 | 4.55E-02 | NA | NA | Validation |
| AFP | Wald ratio | 1 | 1.0016 | 1.0006 | 1.0027 | 2.70E-03 | NA | NA | Validation |
| ALPP | Wald ratio | 1 | 1.0008 | 1.0003 | 1.0014 | 2.70E-03 | NA | NA | Validation |
| BTNL8 | Wald ratio | 1 | 0.9993 | 0.9986 | 1.0000 | 4.55E-02 | NA | NA | Validation |
| C1RL | Wald ratio | 1 | 0.9993 | 0.9985 | 1.0000 | 4.55E-02 | NA | NA | Validation |
| CA10 | Wald ratio | 1 | 0.9985 | 0.9975 | 0.9995 | 2.70E-03 | NA | NA | Validation |
| CCL15 | Wald ratio | 1 | 0.9997 | 0.9995 | 0.9999 | 1.24E-02 | NA | NA | Validation |
| CCL5 | Wald ratio | 1 | 1.0012 | 1.0000 | 1.0023 | 4.55E-02 | NA | NA | Validation |
| CD274 | Wald ratio | 1 | 0.9990 | 0.9984 | 0.9997 | 2.70E-03 | NA | NA | Validation |
| CD300E | Wald ratio | 1 | 1.0007 | 1.0000 | 1.0015 | 4.55E-02 | NA | NA | Validation |
| CD48 | Wald ratio | 1 | 0.9992 | 0.9985 | 1.0000 | 4.55E-02 | NA | NA | Validation |
| CDON | Wald ratio | 1 | 0.9995 | 0.9990 | 1.0000 | 4.55E-02 | NA | NA | Validation |
| CFH | Wald ratio | 1 | 0.9995 | 0.9989 | 1.0000 | 4.55E-02 | NA | NA | Validation |
| CFHR5 | Wald ratio | 1 | 0.9993 | 0.9988 | 0.9997 | 2.70E-03 | NA | NA | Validation |
| CLPS | Wald ratio | 1 | 0.9996 | 0.9991 | 1.0000 | 4.55E-02 | NA | NA | Validation |
| CPA4 | Wald ratio | 1 | 1.0003 | 1.0001 | 1.0004 | 2.70E-03 | NA | NA | Validation |
| CPB2 | Wald ratio | 1 | 0.9998 | 0.9995 | 1.0000 | 4.55E-02 | NA | NA | Validation |
| CPM | Wald ratio | 1 | 0.9989 | 0.9979 | 1.0000 | 4.55E-02 | NA | NA | Validation |
| CPZ | Wald ratio | 1 | 0.9985 | 0.9975 | 0.9995 | 2.70E-03 | NA | NA | Validation |
| CRLF1 | Wald ratio | 1 | 1.0007 | 1.0000 | 1.0014 | 4.55E-02 | NA | NA | Validation |
| CST3 | Wald ratio | 1 | 0.9995 | 0.9990 | 1.0000 | 4.55E-02 | NA | NA | Validation |
| CTSD | Wald ratio | 1 | 0.9991 | 0.9986 | 0.9997 | 2.70E-03 | NA | NA | Validation |
| CXCL16 | Wald ratio | 1 | 0.9987 | 0.9978 | 0.9995 | 2.70E-03 | NA | NA | Validation |
| ECM1 | Wald ratio | 1 | 1.0005 | 1.0002 | 1.0007 | 6.33E-05 | NA | NA | Validation |
| ESAM | Wald ratio | 1 | 0.9987 | 0.9979 | 0.9996 | 2.70E-03 | NA | NA | Validation |
| FCN1 | Wald ratio | 1 | 1.0003 | 1.0000 | 1.0007 | 4.55E-02 | NA | NA | Validation |
| FCRL6 | Wald ratio | 1 | 1.0003 | 1.0000 | 1.0007 | 4.55E-02 | NA | NA | Validation |
| FLRT3 | Wald ratio | 1 | 1.0005 | 1.0000 | 1.0009 | 4.55E-02 | NA | NA | Validation |
| FN1 | Wald ratio | 1 | 1.0004 | 1.0001 | 1.0007 | 2.70E-03 | NA | NA | Validation |
| FRZB | Wald ratio | 1 | 1.0007 | 1.0000 | 1.0014 | 4.55E-02 | NA | NA | Validation |
| FUT5 | Wald ratio | 1 | 0.9997 | 0.9993 | 1.0000 | 4.55E-02 | NA | NA | Validation |
| GFRA1 | Wald ratio | 1 | 0.9992 | 0.9985 | 1.0000 | 4.55E-02 | NA | NA | Validation |
| GFRA2 | Wald ratio | 1 | 1.0007 | 1.0000 | 1.0013 | 4.55E-02 | NA | NA | Validation |
| GPC5 | Inverse variance weighted | 2 | 1.0001 | 1.0000 | 1.0002 | 1.61E-02 | NA | NA | Validation |
| GSTA1 | Wald ratio | 1 | 1.0005 | 1.0000 | 1.0010 | 4.55E-02 | NA | NA | Validation |
| GSTP1 | Wald ratio | 1 | 1.0011 | 1.0000 | 1.0022 | 4.55E-02 | NA | NA | Validation |
| HGFAC | Wald ratio | 1 | 1.0006 | 1.0002 | 1.0010 | 2.70E-03 | NA | NA | Validation |
| ICAM1 | Wald ratio | 1 | 0.9998 | 0.9997 | 1.0000 | 4.55E-02 | NA | NA | Validation |
| IDUA | Wald ratio | 1 | 1.0003 | 1.0000 | 1.0007 | 4.55E-02 | NA | NA | Validation |
| IGDCC4 | Wald ratio | 1 | 0.9985 | 0.9974 | 0.9997 | 1.24E-02 | NA | NA | Validation |
| IL12B | Wald ratio | 1 | 0.9994 | 0.9987 | 1.0000 | 4.55E-02 | NA | NA | Validation |
| IL18 | Wald ratio | 1 | 1.0007 | 1.0000 | 1.0014 | 4.55E-02 | NA | NA | Validation |
| IL18RAP | Wald ratio | 1 | 1.0004 | 1.0000 | 1.0008 | 4.55E-02 | NA | NA | Validation |
| ISG15 | Wald ratio | 1 | 0.9992 | 0.9985 | 1.0000 | 4.55E-02 | NA | NA | Validation |
| KIAA1161 | Wald ratio | 1 | 0.9994 | 0.9987 | 1.0000 | 4.55E-02 | NA | NA | Validation |
| KLK10 | Wald ratio | 1 | 1.0005 | 1.0000 | 1.0010 | 4.55E-02 | NA | NA | Validation |
| KLK13 | Wald ratio | 1 | 1.0005 | 1.0000 | 1.0010 | 4.55E-02 | NA | NA | Validation |
| KLRB1 | Wald ratio | 1 | 0.9986 | 0.9977 | 0.9995 | 2.70E-03 | NA | NA | Validation |
| LGALS2 | Wald ratio | 1 | 1.0007 | 1.0000 | 1.0014 | 4.55E-02 | NA | NA | Validation |
| LILRB2 | Wald ratio | 1 | 0.9998 | 0.9997 | 1.0000 | 4.55E-02 | NA | NA | Validation |
| LRP8 | Wald ratio | 1 | 0.9988 | 0.9976 | 1.0000 | 4.55E-02 | NA | NA | Validation |
| MAN1C1 | Wald ratio | 1 | 1.0012 | 1.0000 | 1.0023 | 4.55E-02 | NA | NA | Validation |
| MGAT2 | Wald ratio | 1 | 1.0010 | 1.0000 | 1.0020 | 4.55E-02 | NA | NA | Validation |
| MGP | Wald ratio | 1 | 0.9992 | 0.9985 | 1.0000 | 4.55E-02 | NA | NA | Validation |
| MTHFS | Wald ratio | 1 | 0.9991 | 0.9981 | 1.0000 | 4.55E-02 | NA | NA | Validation |
| NCAM2 | Wald ratio | 1 | 1.0005 | 1.0000 | 1.0011 | 4.55E-02 | NA | NA | Validation |
| NEGR1 | Wald ratio | 1 | 1.0020 | 1.0007 | 1.0034 | 2.70E-03 | NA | NA | Validation |
| NLGN2 | Wald ratio | 1 | 0.9980 | 0.9965 | 0.9996 | 1.24E-02 | NA | NA | Validation |
| NRP1 | Wald ratio | 1 | 1.0011 | 1.0004 | 1.0018 | 2.70E-03 | NA | NA | Validation |
| OAS1 | Wald ratio | 1 | 1.0011 | 1.0004 | 1.0018 | 2.70E-03 | NA | NA | Validation |
| OSMR | Wald ratio | 1 | 0.9990 | 0.9981 | 1.0000 | 4.55E-02 | NA | NA | Validation |
| OXT | Wald ratio | 1 | 1.1476 | 1.0165 | 1.2956 | 2.61E-02 | NA | NA | Validation |
| PDCD5 | Wald ratio | 1 | 0.9994 | 0.9989 | 1.0000 | 4.55E-02 | NA | NA | Validation |
| PIP | Wald ratio | 1 | 0.9989 | 0.9979 | 1.0000 | 4.55E-02 | NA | NA | Validation |
| PLA2G2A | Wald ratio | 1 | 0.9998 | 0.9996 | 1.0000 | 4.55E-02 | NA | NA | Validation |
| PLEKHA7 | Wald ratio | 1 | 1.0014 | 1.0000 | 1.0029 | 4.55E-02 | NA | NA | Validation |
| PLXNA1 | Wald ratio | 1 | 0.9989 | 0.9978 | 1.0000 | 4.55E-02 | NA | NA | Validation |
| PNLIPRP2 | Wald ratio | 1 | 1.0002 | 1.0000 | 1.0004 | 4.55E-02 | NA | NA | Validation |
| PPIE | Wald ratio | 1 | 1.0008 | 1.0003 | 1.0013 | 2.70E-03 | NA | NA | Validation |
| PPP3CA;PPP3R1 | Wald ratio | 1 | 0.9995 | 0.9989 | 1.0000 | 4.55E-02 | NA | NA | Validation |
| PYY | Wald ratio | 1 | 0.9987 | 0.9975 | 1.0000 | 4.55E-02 | NA | NA | Validation |
| PZP | Wald ratio | 1 | 1.0004 | 1.0000 | 1.0008 | 4.55E-02 | NA | NA | Validation |
| QPCTL | Wald ratio | 1 | 0.9993 | 0.9986 | 1.0000 | 4.55E-02 | NA | NA | Validation |
| REG3G | Wald ratio | 1 | 0.9992 | 0.9988 | 0.9996 | 6.33E-05 | NA | NA | Validation |
| RELT | Wald ratio | 1 | 1.0005 | 1.0000 | 1.0010 | 4.55E-02 | NA | NA | Validation |
| SCARF2 | Wald ratio | 1 | 0.9987 | 0.9980 | 0.9993 | 6.33E-05 | NA | NA | Validation |
| SELPLG | Wald ratio | 1 | 1.0010 | 1.0000 | 1.0019 | 4.55E-02 | NA | NA | Validation |
| SERPINA1 | Wald ratio | 1 | 0.9995 | 0.9991 | 1.0000 | 4.55E-02 | NA | NA | Validation |
| SERPINA4 | Inverse variance weighted | 2 | 1.0002 | 1.0000 | 1.0003 | 8.55E-03 | NA | NA | Validation |
| SERPINF1 | Wald ratio | 1 | 0.9996 | 0.9991 | 1.0000 | 4.55E-02 | NA | NA | Validation |
| SFTPB | Wald ratio | 1 | 1.0003 | 1.0000 | 1.0006 | 4.55E-02 | NA | NA | Validation |
| SHBG | Wald ratio | 1 | 1.0009 | 1.0000 | 1.0019 | 4.55E-02 | NA | NA | Validation |
| SPARCL1 | Inverse variance weighted | 2 | 0.9996 | 0.9993 | 0.9998 | 5.28E-05 | NA | NA | Validation |
| SPINK1 | Wald ratio | 1 | 0.9991 | 0.9981 | 1.0000 | 4.55E-02 | NA | NA | Validation |
| SPINK2 | Wald ratio | 1 | 1.0011 | 1.0003 | 1.0019 | 5.96E-03 | NA | NA | Validation |
| SPINK5 | Wald ratio | 1 | 0.9987 | 0.9974 | 1.0000 | 4.55E-02 | NA | NA | Validation |
| ST3GAL1 | Wald ratio | 1 | 1.0008 | 1.0000 | 1.0015 | 4.55E-02 | NA | NA | Validation |
| TAPBPL | Wald ratio | 1 | 1.0002 | 1.0000 | 1.0003 | 4.55E-02 | NA | NA | Validation |
| TMEM132C | Wald ratio | 1 | 0.9987 | 0.9978 | 0.9995 | 2.70E-03 | NA | NA | Validation |
| TNFSF12;TNFSF12-TNFSF13 | Wald ratio | 1 | 1.0005 | 1.0000 | 1.0010 | 4.55E-02 | NA | NA | Validation |
| TREM2 | Wald ratio | 1 | 0.9978 | 0.9956 | 1.0000 | 4.55E-02 | NA | NA | Validation |
| TYRO3 | Wald ratio | 1 | 0.9988 | 0.9975 | 1.0000 | 4.55E-02 | NA | NA | Validation |
| UNC5C | Wald ratio | 1 | 0.9993 | 0.9985 | 1.0000 | 4.55E-02 | NA | NA | Validation |
| UNC5D | Wald ratio | 1 | 1.0014 | 1.0000 | 1.0028 | 4.55E-02 | NA | NA | Validation |
| UROS | Wald ratio | 1 | 0.9991 | 0.9983 | 1.0000 | 4.55E-02 | NA | NA | Validation |
| VIT | Wald ratio | 1 | 0.9994 | 0.9989 | 1.0000 | 4.55E-02 | NA | NA | Validation |
| VWA2 | Wald ratio | 1 | 1.0009 | 1.0000 | 1.0018 | 4.55E-02 | NA | NA | Validation |
| VWC2 | Wald ratio | 1 | 0.9988 | 0.9977 | 1.0000 | 4.55E-02 | NA | NA | Validation |
| WARS | Wald ratio | 1 | 1.0012 | 1.0004 | 1.0019 | 2.70E-03 | NA | NA | Validation |
| WFIKKN1 | Wald ratio | 1 | 0.9990 | 0.9981 | 1.0000 | 4.55E-02 | NA | NA | Validation |
| WFIKKN2 | Wald ratio | 1 | 0.9997 | 0.9994 | 1.0000 | 4.55E-02 | NA | NA | Validation |
| WISP2 | Wald ratio | 1 | 1.0009 | 1.0000 | 1.0019 | 4.55E-02 | NA | NA | Validation |
| ZG16B | Wald ratio | 1 | 1.0013 | 1.0000 | 1.0025 | 4.55E-02 | NA | NA | Validation |

**Notes:** “Proteins” column refers to the name of a protein; “Methods” column refers to the statistical method used to calculated the causal effect; “NSNP” column refers to the number of single nucleotide polymorphism used in causal estimates; “OR” column refers to the odds ratio; “Lower of 95%CI” column refers to the lower limit of 95% confidence interval; “Upper of 95%CI” refers to the upper limit of 95% confidence interval; “P” column refers to the p-value of odds ratio; “P_het_” column refers to the p-value of heterogeneity test; “P_pleio_” column refers to the p-value of horizontal pleiotropy test; “Stage” column refers to the “Discovery” or “Validation” stage.

**Supplementary Table 2: The results of enrichment analyses using circulating proteins closely associated with idiopathic pulmonary fibrosis.**

| **Gene Set** | **Description** | **Size** | **Expect** | **Ratio** | **P** | **Database** |
| --- | --- | --- | --- | --- | --- | --- |
| R-HSA-1566977 | Fibronectin matrix formation | 6 | 0.0022 | 462.7900 | 2.16E-03 | Reactome |
| R-HSA-202733 | Cell surface interactions at the vascular wall | 137 | 0.0493 | 40.5360 | 1.01E-03 | Reactome |
| R-HSA-9006934 | Signaling by Receptor Tyrosine Kinases | 455 | 0.1639 | 18.3080 | 4.16E-04 | Reactome |
| C0206062 | Lung Diseases, Interstitial | 5 | 0.0018 | 555.3400 | 1.80E-03 | DisGeNET |
| C0034050 | Pulmonary Alveolar Proteinosis | 6 | 0.0022 | 462.7900 | 2.16E-03 | DisGeNET |
| C0000786 | Spontaneous abortion | 114 | 0.0411 | 48.7140 | 7.02E-04 | DisGeNET |
| C0027626 | Neoplasm Invasiveness | 143 | 0.0515 | 38.8350 | 1.10E-03 | DisGeNET |
| DB04573 | Estriol | 5 | 0.0018 | 555.3400 | 1.80E-03 | DrugBank |
| DB04824 | Phenolphthalein | 5 | 0.0018 | 555.3400 | 1.80E-03 | DrugBank |
| DB00039 | Palifermin | 6 | 0.0022 | 462.7900 | 2.16E-03 | DrugBank |
| DB00539 | Toremifene | 6 | 0.0022 | 462.7900 | 2.16E-03 | DrugBank |
| DB01094 | Hesperetin | 6 | 0.0022 | 462.7900 | 2.16E-03 | DrugBank |
| DB01185 | Fluoxymesterone | 6 | 0.0022 | 462.7900 | 2.16E-03 | DrugBank |
| DB02342 | 2-Methoxyestradiol | 6 | 0.0022 | 462.7900 | 2.16E-03 | DrugBank |
| DB11619 | Gestrinone | 6 | 0.0022 | 462.7900 | 2.16E-03 | DrugBank |
| DB01593 | Zinc | 121 | 0.0436 | 45.8960 | 7.91E-04 | DrugBank |

**Supplementary Table 3: Associations of circulation proteins with chronic obstructive pulmonary disease.**

| **Proteins** | **Methods** | **NSNP** | **OR** | **Lower of 95%CI** | **Upper of 95%CI** | **P** | **P_het_** | **P_pleio_** | **Stage** |
| --- | --- | --- | --- | --- | --- | --- | --- | --- | --- |
| A2ML1 | Inverse variance weighted | 2 | 1.0848 | 1.0223 | 1.1512 | 7.21E-03 | NA | NA | Discovery |
| ACAA1 | Wald ratio | 1 | 1.5390 | 1.0242 | 2.3126 | 3.80E-02 | NA | NA | Discovery |
| ACAN | Inverse variance weighted | 2 | 1.1064 | 1.0671 | 1.1472 | 4.47E-08 | NA | NA | Discovery |
| ACE | Inverse variance weighted | 2 | 0.9561 | 0.9403 | 0.9721 | 1.14E-07 | NA | NA | Discovery |
| ACYP2 | Wald ratio | 1 | 1.6269 | 1.0298 | 2.5702 | 3.70E-02 | NA | NA | Discovery |
| ADAM23 | Inverse variance weighted | 3 | 0.8061 | 0.7235 | 0.8982 | 9.33E-05 | NA | NA | Discovery |
| ADAM23 | Weighted median | 3 | 0.8068 | 0.6970 | 0.9338 | 4.01E-03 | NA | NA | Discovery |
| ADAMTS13 | Inverse variance weighted | 3 | 0.9441 | 0.9180 | 0.9709 | 5.57E-05 | NA | NA | Discovery |
| AFM | Inverse variance weighted | 2 | 0.9359 | 0.8967 | 0.9768 | 2.42E-03 | NA | NA | Discovery |
| AGRN | Inverse variance weighted | 4 | 0.9152 | 0.8625 | 0.9712 | 3.45E-03 | 0.5162 | 0.7521 | Discovery |
| AGRN | Weighted median | 4 | 0.9112 | 0.8493 | 0.9777 | 9.68E-03 | 0.5162 | 0.7521 | Discovery |
| ALCAM | Inverse variance weighted | 3 | 1.0855 | 1.0082 | 1.1688 | 2.95E-02 | NA | NA | Discovery |
| ALDH1A1 | Wald ratio | 1 | 0.7827 | 0.6138 | 0.9980 | 4.82E-02 | NA | NA | Discovery |
| ALDH2 | Wald ratio | 1 | 0.6653 | 0.4698 | 0.9421 | 2.17E-02 | NA | NA | Discovery |
| ANXA1 | Inverse variance weighted | 2 | 0.9273 | 0.9094 | 0.9455 | 2.88E-14 | NA | NA | Discovery |
| ANXA11 | Inverse variance weighted | 3 | 0.8995 | 0.8204 | 0.9862 | 2.40E-02 | NA | NA | Discovery |
| ANXA7 | Wald ratio | 1 | 0.6035 | 0.3834 | 0.9500 | 2.92E-02 | NA | NA | Discovery |
| APCS | Inverse variance weighted | 3 | 0.8849 | 0.7857 | 0.9965 | 4.36E-02 | NA | NA | Discovery |
| APCS | Weighted median | 3 | 0.8744 | 0.7845 | 0.9747 | 1.54E-02 | NA | NA | Discovery |
| APLP2 | Inverse variance weighted | 2 | 1.0927 | 1.0211 | 1.1694 | 1.04E-02 | NA | NA | Discovery |
| APMAP | Inverse variance weighted | 2 | 0.8493 | 0.8396 | 0.8592 | 4.12E-169 | NA | NA | Discovery |
| APOA1BP | Inverse variance weighted | 2 | 1.0284 | 1.0102 | 1.0470 | 2.09E-03 | NA | NA | Discovery |
| APOA5 | Wald ratio | 1 | 1.1759 | 1.0771 | 1.2837 | 2.94E-04 | NA | NA | Discovery |
| APOBEC3G | Inverse variance weighted | 2 | 1.1393 | 1.0957 | 1.1846 | 5.77E-11 | NA | NA | Discovery |
| APOF | Inverse variance weighted | 2 | 1.1716 | 1.1203 | 1.2252 | 4.09E-12 | NA | NA | Discovery |
| ARHGEF25 | Inverse variance weighted | 2 | 0.7514 | 0.6177 | 0.9140 | 4.24E-03 | NA | NA | Discovery |
| ARSB | Inverse variance weighted | 2 | 1.1763 | 1.1027 | 1.2547 | 8.32E-07 | NA | NA | Discovery |
| AZGP1 | Inverse variance weighted | 2 | 0.9130 | 0.9025 | 0.9236 | 8.73E-54 | NA | NA | Discovery |
| B3GALTL | Inverse variance weighted | 3 | 1.0803 | 1.0470 | 1.1148 | 1.38E-06 | NA | NA | Discovery |
| BIN1 | Wald ratio | 1 | 0.8106 | 0.6805 | 0.9655 | 1.86E-02 | NA | NA | Discovery |
| BLVRA | Wald ratio | 1 | 0.6685 | 0.4774 | 0.9360 | 1.90E-02 | NA | NA | Discovery |
| BPIFA2 | Inverse variance weighted | 2 | 1.0538 | 1.0313 | 1.0769 | 2.04E-06 | NA | NA | Discovery |
| BRSK2 | Wald ratio | 1 | 1.2534 | 1.0890 | 1.4427 | 1.65E-03 | NA | NA | Discovery |
| BST1 | Inverse variance weighted | 2 | 0.8683 | 0.7839 | 0.9617 | 6.75E-03 | NA | NA | Discovery |
| BTN3A3 | Inverse variance weighted | 2 | 0.9369 | 0.8868 | 0.9899 | 2.03E-02 | NA | NA | Discovery |
| C1QC | Inverse variance weighted | 7 | 1.0781 | 1.0522 | 1.1047 | 1.43E-09 | 0.9766 | 0.9500 | Discovery |
| C1QC | Weighted median | 7 | 1.0784 | 1.0109 | 1.1505 | 2.21E-02 | 0.9766 | 0.9500 | Discovery |
| C2 | Wald ratio | 1 | 1.2296 | 1.0352 | 1.4604 | 1.86E-02 | NA | NA | Discovery |
| CA1 | Inverse variance weighted | 2 | 0.9184 | 0.8436 | 0.9999 | 4.96E-02 | NA | NA | Discovery |
| CA10 | Inverse variance weighted | 4 | 0.9116 | 0.8428 | 0.9861 | 2.09E-02 | 0.4409 | 0.3480 | Discovery |
| CASP3 | Inverse variance weighted | 2 | 0.8740 | 0.8114 | 0.9413 | 3.78E-04 | NA | NA | Discovery |
| CAT | Inverse variance weighted | 2 | 1.0686 | 1.0381 | 1.1000 | 7.13E-06 | NA | NA | Discovery |
| CCDC126 | Inverse variance weighted | 3 | 0.9699 | 0.9504 | 0.9898 | 3.21E-03 | NA | NA | Discovery |
| CCL14 | Inverse variance weighted | 2 | 1.0385 | 1.0364 | 1.0406 | 1.05E-291 | NA | NA | Discovery |
| CCL15 | Inverse variance weighted | 2 | 1.0938 | 1.0800 | 1.1078 | 1.36E-43 | NA | NA | Discovery |
| CCL22 | Inverse variance weighted | 2 | 1.1065 | 1.0564 | 1.1589 | 1.83E-05 | NA | NA | Discovery |
| CD274 | Inverse variance weighted | 2 | 0.9714 | 0.9469 | 0.9965 | 2.61E-02 | NA | NA | Discovery |
| CD33 | Inverse variance weighted | 4 | 0.9631 | 0.9486 | 0.9779 | 1.33E-06 | 0.9416 | 0.9310 | Discovery |
| CD46 | Inverse variance weighted | 2 | 1.0141 | 1.0030 | 1.0253 | 1.25E-02 | NA | NA | Discovery |
| CD59 | Inverse variance weighted | 2 | 1.0844 | 1.0203 | 1.1525 | 9.16E-03 | NA | NA | Discovery |
| CD72 | Inverse variance weighted | 2 | 1.1609 | 1.0514 | 1.2819 | 3.16E-03 | NA | NA | Discovery |
| CDON | Inverse variance weighted | 2 | 1.0756 | 1.0430 | 1.1091 | 3.37E-06 | NA | NA | Discovery |
| CFHR1 | Inverse variance weighted | 2 | 0.6237 | 0.4310 | 0.9025 | 1.23E-02 | NA | NA | Discovery |
| CHL1 | Inverse variance weighted | 6 | 1.0586 | 1.0100 | 1.1096 | 1.77E-02 | 0.6794 | 0.5549 | Discovery |
| CHRD | Wald ratio | 1 | 1.4910 | 1.0044 | 2.2135 | 4.75E-02 | NA | NA | Discovery |
| CHST12 | Inverse variance weighted | 2 | 0.8850 | 0.7858 | 0.9966 | 4.38E-02 | NA | NA | Discovery |
| CHST15 | Inverse variance weighted | 2 | 0.8838 | 0.8312 | 0.9398 | 8.10E-05 | NA | NA | Discovery |
| CHST9 | Inverse variance weighted | 3 | 0.9555 | 0.9349 | 0.9765 | 4.14E-05 | NA | NA | Discovery |
| CLEC1B | Inverse variance weighted | 2 | 0.9410 | 0.9269 | 0.9553 | 2.39E-15 | NA | NA | Discovery |
| CLMP | Wald ratio | 1 | 1.1254 | 1.0071 | 1.2575 | 3.72E-02 | NA | NA | Discovery |
| CLPS | Weighted median | 8 | 0.9176 | 0.8491 | 0.9915 | 2.96E-02 | 0.0041 | 0.3466 | Discovery |
| CMPK1 | Inverse variance weighted | 2 | 1.0189 | 1.0120 | 1.0260 | 8.82E-08 | NA | NA | Discovery |
| COL2A1 | Wald ratio | 1 | 0.3173 | 0.1639 | 0.6142 | 6.58E-04 | NA | NA | Discovery |
| COL3A1 | Inverse variance weighted | 2 | 0.9213 | 0.8886 | 0.9552 | 8.80E-06 | NA | NA | Discovery |
| COL9A1 | Wald ratio | 1 | 1.7457 | 1.1862 | 2.5691 | 4.71E-03 | NA | NA | Discovery |
| COLEC11 | Inverse variance weighted | 3 | 0.9144 | 0.8853 | 0.9445 | 6.20E-08 | NA | NA | Discovery |
| COLGALT1 | Inverse variance weighted | 2 | 1.0506 | 1.0033 | 1.1002 | 3.58E-02 | NA | NA | Discovery |
| CPM | Inverse variance weighted | 3 | 0.8962 | 0.8877 | 0.9049 | 1.83E-110 | NA | NA | Discovery |
| CPXM1 | Inverse variance weighted | 6 | 0.9402 | 0.9188 | 0.9622 | 1.66E-07 | 0.9557 | 0.7132 | Discovery |
| CPXM1 | Weighted median | 6 | 0.9420 | 0.8888 | 0.9982 | 4.35E-02 | 0.9557 | 0.7132 | Discovery |
| CR2 | Inverse variance weighted | 4 | 1.1441 | 1.0628 | 1.2317 | 3.46E-04 | 0.7273 | 0.7105 | Discovery |
| CR2 | Weighted median | 4 | 1.1584 | 1.0166 | 1.3199 | 2.73E-02 | 0.7273 | 0.7105 | Discovery |
| CRLF1 | Inverse variance weighted | 2 | 0.9197 | 0.8781 | 0.9633 | 3.96E-04 | NA | NA | Discovery |
| CRYBB1 | Inverse variance weighted | 2 | 1.2292 | 1.0089 | 1.4975 | 4.05E-02 | NA | NA | Discovery |
| CRYGD | Inverse variance weighted | 2 | 1.0455 | 1.0418 | 1.0492 | 1.92E-132 | NA | NA | Discovery |
| CRYZ | Inverse variance weighted | 2 | 1.0127 | 1.0004 | 1.0252 | 4.24E-02 | NA | NA | Discovery |
| CSF1R | Inverse variance weighted | 3 | 1.1194 | 1.0316 | 1.2148 | 6.84E-03 | NA | NA | Discovery |
| CXCL12 | Weighted median | 5 | 1.1728 | 1.0020 | 1.3726 | 4.71E-02 | 0.0007 | 0.8077 | Discovery |
| DCTD | Wald ratio | 1 | 0.7473 | 0.5638 | 0.9905 | 4.28E-02 | NA | NA | Discovery |
| DCXR | Inverse variance weighted | 2 | 0.9505 | 0.9444 | 0.9566 | 6.37E-54 | NA | NA | Discovery |
| DECR2 | Inverse variance weighted | 2 | 1.0608 | 1.0513 | 1.0703 | 4.43E-38 | NA | NA | Discovery |
| DLK1 | Weighted median | 4 | 0.9099 | 0.8481 | 0.9761 | 8.43E-03 | 0.2099 | 0.1773 | Discovery |
| DNAJB12 | Wald ratio | 1 | 0.6985 | 0.5143 | 0.9489 | 2.17E-02 | NA | NA | Discovery |
| DPEP1 | Inverse variance weighted | 4 | 0.8403 | 0.7617 | 0.9269 | 5.08E-04 | 0.6311 | 0.4384 | Discovery |
| DPEP1 | Weighted median | 4 | 0.8334 | 0.7151 | 0.9714 | 1.97E-02 | 0.6311 | 0.4384 | Discovery |
| DSG2 | Inverse variance weighted | 3 | 1.1184 | 1.0348 | 1.2088 | 4.75E-03 | NA | NA | Discovery |
| DSG2 | Weighted median | 3 | 1.1235 | 1.0017 | 1.2600 | 4.67E-02 | NA | NA | Discovery |
| DUT | Wald ratio | 1 | 1.4937 | 1.0118 | 2.2052 | 4.35E-02 | NA | NA | Discovery |
| EDAR | Inverse variance weighted | 3 | 0.9636 | 0.9545 | 0.9728 | 2.29E-14 | NA | NA | Discovery |
| EFNB2 | Wald ratio | 1 | 0.5955 | 0.3972 | 0.8929 | 1.21E-02 | NA | NA | Discovery |
| EGFLAM | Inverse variance weighted | 3 | 1.2054 | 1.0874 | 1.3362 | 3.80E-04 | NA | NA | Discovery |
| EGFLAM | Weighted median | 3 | 1.1905 | 1.0518 | 1.3474 | 5.80E-03 | NA | NA | Discovery |
| EGLN1 | Inverse variance weighted | 2 | 1.0427 | 1.0304 | 1.0551 | 4.61E-12 | NA | NA | Discovery |
| ELMO1 | Inverse variance weighted | 3 | 1.1971 | 1.1084 | 1.2929 | 4.61E-06 | NA | NA | Discovery |
| ENPP6 | Inverse variance weighted | 2 | 0.8557 | 0.7782 | 0.9410 | 1.31E-03 | NA | NA | Discovery |
| ENPP7 | Inverse variance weighted | 2 | 1.1320 | 1.1188 | 1.1453 | 1.03E-95 | NA | NA | Discovery |
| EPHA2 | Inverse variance weighted | 2 | 0.9037 | 0.8757 | 0.9325 | 2.74E-10 | NA | NA | Discovery |
| ERAP1 | Inverse variance weighted | 2 | 1.0817 | 1.0281 | 1.1382 | 2.48E-03 | NA | NA | Discovery |
| FAM172A | Wald ratio | 1 | 0.6737 | 0.4588 | 0.9892 | 4.39E-02 | NA | NA | Discovery |
| FAM177A1 | Inverse variance weighted | 2 | 0.9331 | 0.9235 | 0.9429 | 4.41E-39 | NA | NA | Discovery |
| FAP | Inverse variance weighted | 2 | 1.2012 | 1.1918 | 1.2106 | 0.00E+00 | NA | NA | Discovery |
| FAS | Inverse variance weighted | 3 | 0.9514 | 0.9078 | 0.9971 | 3.73E-02 | NA | NA | Discovery |
| FBLN5 | Inverse variance weighted | 2 | 1.2194 | 1.0926 | 1.3609 | 3.98E-04 | NA | NA | Discovery |
| FCGR3B | Inverse variance weighted | 2 | 1.1029 | 1.0057 | 1.2095 | 3.74E-02 | NA | NA | Discovery |
| FGFR3 | Inverse variance weighted | 3 | 0.9155 | 0.8931 | 0.9384 | 2.44E-12 | NA | NA | Discovery |
| FJX1 | Inverse variance weighted | 6 | 0.9257 | 0.8667 | 0.9888 | 2.18E-02 | 0.6470 | 0.7976 | Discovery |
| FKBP4 | Inverse variance weighted | 2 | 1.0950 | 1.0399 | 1.1530 | 5.65E-04 | NA | NA | Discovery |
| FLRT2 | Inverse variance weighted | 3 | 1.0238 | 1.0080 | 1.0398 | 3.09E-03 | NA | NA | Discovery |
| FN1 | Inverse variance weighted | 2 | 0.9107 | 0.8659 | 0.9577 | 2.71E-04 | NA | NA | Discovery |
| FSTL1 | Inverse variance weighted | 2 | 1.1001 | 1.0334 | 1.1711 | 2.79E-03 | NA | NA | Discovery |
| GLB1 | Wald ratio | 1 | 0.5610 | 0.3292 | 0.9561 | 3.36E-02 | NA | NA | Discovery |
| GLIPR2 | Inverse variance weighted | 4 | 0.9438 | 0.9287 | 0.9590 | 1.60E-12 | 0.9774 | 0.8129 | Discovery |
| GLRX2 | Inverse variance weighted | 2 | 0.9128 | 0.8801 | 0.9467 | 9.63E-07 | NA | NA | Discovery |
| GM2A | Inverse variance weighted | 3 | 1.0861 | 1.0418 | 1.1323 | 1.02E-04 | NA | NA | Discovery |
| GNMT | Inverse variance weighted | 2 | 0.9368 | 0.9342 | 0.9395 | 0.00E+00 | NA | NA | Discovery |
| GOLM1 | Weighted median | 3 | 1.0621 | 1.0201 | 1.1060 | 3.48E-03 | NA | NA | Discovery |
| GPCPD1 | Inverse variance weighted | 2 | 0.8362 | 0.7539 | 0.9274 | 7.07E-04 | NA | NA | Discovery |
| GSTM4 | Inverse variance weighted | 5 | 0.9358 | 0.9196 | 0.9522 | 8.65E-14 | 0.9771 | 0.7395 | Discovery |
| GSTM4 | Weighted median | 5 | 0.9319 | 0.8754 | 0.9921 | 2.73E-02 | 0.9771 | 0.7395 | Discovery |
| GSTP1 | Inverse variance weighted | 2 | 0.9194 | 0.8643 | 0.9781 | 7.74E-03 | NA | NA | Discovery |
| GZMK | Inverse variance weighted | 3 | 0.9050 | 0.8438 | 0.9706 | 5.19E-03 | NA | NA | Discovery |
| H6PD | Inverse variance weighted | 2 | 1.0623 | 1.0420 | 1.0830 | 8.94E-10 | NA | NA | Discovery |
| HAPLN4 | Wald ratio | 1 | 0.6606 | 0.4577 | 0.9536 | 2.69E-02 | NA | NA | Discovery |
| HBZ | Inverse variance weighted | 2 | 0.9522 | 0.9420 | 0.9625 | 4.85E-19 | NA | NA | Discovery |
| HEXB | Inverse variance weighted | 2 | 1.0445 | 1.0210 | 1.0685 | 1.79E-04 | NA | NA | Discovery |
| HHIP | Wald ratio | 1 | 1.2723 | 1.0622 | 1.5240 | 8.91E-03 | NA | NA | Discovery |
| HMHA1 | Inverse variance weighted | 2 | 1.1426 | 1.0144 | 1.2871 | 2.82E-02 | NA | NA | Discovery |
| HRG | Inverse variance weighted | 2 | 1.1842 | 1.1168 | 1.2556 | 1.51E-08 | NA | NA | Discovery |
| HS6ST1 | Inverse variance weighted | 2 | 0.9021 | 0.8870 | 0.9175 | 5.70E-33 | NA | NA | Discovery |
| HTN3 | Inverse variance weighted | 2 | 0.7562 | 0.7531 | 0.7593 | 0.00E+00 | NA | NA | Discovery |
| ICOSLG | Wald ratio | 1 | 1.4993 | 1.0231 | 2.1972 | 3.78E-02 | NA | NA | Discovery |
| IGFALS | Inverse variance weighted | 2 | 1.1962 | 1.1447 | 1.2500 | 1.49E-15 | NA | NA | Discovery |
| IGFBP7 | Inverse variance weighted | 3 | 0.9243 | 0.8790 | 0.9720 | 2.18E-03 | NA | NA | Discovery |
| IGFBP7 | Weighted median | 3 | 0.9271 | 0.8692 | 0.9889 | 2.15E-02 | NA | NA | Discovery |
| IGFLR1 | Wald ratio | 1 | 0.7681 | 0.6533 | 0.9030 | 1.40E-03 | NA | NA | Discovery |
| IL15RA | Inverse variance weighted | 4 | 1.0535 | 1.0201 | 1.0879 | 1.50E-03 | 0.9093 | 0.7426 | Discovery |
| IL1R1 | Inverse variance weighted | 2 | 1.0605 | 1.0440 | 1.0772 | 1.92E-13 | NA | NA | Discovery |
| IL1R2 | Inverse variance weighted | 2 | 1.1971 | 1.0968 | 1.3066 | 5.60E-05 | NA | NA | Discovery |
| IL1RN | Inverse variance weighted | 2 | 0.9592 | 0.9525 | 0.9660 | 7.38E-31 | NA | NA | Discovery |
| IL27RA | Inverse variance weighted | 2 | 1.0809 | 1.0136 | 1.1528 | 1.78E-02 | NA | NA | Discovery |
| INPP5B | Inverse variance weighted | 3 | 1.1017 | 1.0312 | 1.1770 | 4.08E-03 | NA | NA | Discovery |
| INSR | Wald ratio | 1 | 0.6802 | 0.4875 | 0.9492 | 2.34E-02 | NA | NA | Discovery |
| ITPA | Inverse variance weighted | 2 | 0.8980 | 0.8454 | 0.9540 | 4.87E-04 | NA | NA | Discovery |
| KLC1 | Wald ratio | 1 | 1.3725 | 1.0592 | 1.7786 | 1.66E-02 | NA | NA | Discovery |
| KLK10 | Inverse variance weighted | 5 | 0.9220 | 0.8631 | 0.9849 | 1.59E-02 | 0.5759 | 0.6954 | Discovery |
| KLK13 | Inverse variance weighted | 4 | 0.8679 | 0.7934 | 0.9493 | 1.96E-03 | 0.5691 | 0.6903 | Discovery |
| KLK14 | Weighted median | 9 | 1.1051 | 1.0075 | 1.2121 | 3.41E-02 | 0.0370 | 0.6564 | Discovery |
| KLK8 | Inverse variance weighted | 5 | 1.0826 | 1.0216 | 1.1473 | 7.33E-03 | 0.4484 | 0.4477 | Discovery |
| KLK8 | Weighted median | 5 | 1.0870 | 1.0209 | 1.1575 | 9.19E-03 | 0.4484 | 0.4477 | Discovery |
| KLKB1 | Inverse variance weighted | 2 | 0.9330 | 0.8836 | 0.9851 | 1.24E-02 | NA | NA | Discovery |
| KRT5 | Inverse variance weighted | 2 | 0.9494 | 0.9315 | 0.9676 | 9.00E-08 | NA | NA | Discovery |
| LAMC2 | Inverse variance weighted | 3 | 0.9098 | 0.8787 | 0.9420 | 9.89E-08 | NA | NA | Discovery |
| LAMC2 | Weighted median | 3 | 0.9071 | 0.8612 | 0.9553 | 2.26E-04 | NA | NA | Discovery |
| LAP3 | Wald ratio | 1 | 0.4360 | 0.2544 | 0.7475 | 2.54E-03 | NA | NA | Discovery |
| LARGE | Inverse variance weighted | 2 | 0.9142 | 0.9010 | 0.9275 | 7.80E-34 | NA | NA | Discovery |
| LEPR | Wald ratio | 1 | 1.1388 | 1.0325 | 1.2561 | 9.32E-03 | NA | NA | Discovery |
| LHB | Wald ratio | 1 | 0.8737 | 0.7978 | 0.9568 | 3.59E-03 | NA | NA | Discovery |
| LILRA4 | Inverse variance weighted | 3 | 0.9552 | 0.9193 | 0.9925 | 1.90E-02 | NA | NA | Discovery |
| LOXL3 | Inverse variance weighted | 2 | 0.9134 | 0.8450 | 0.9874 | 2.27E-02 | NA | NA | Discovery |
| LRPAP1 | Inverse variance weighted | 3 | 0.9477 | 0.9234 | 0.9727 | 5.16E-05 | NA | NA | Discovery |
| LYPD3 | Inverse variance weighted | 2 | 1.0716 | 1.0653 | 1.0780 | 5.46E-116 | NA | NA | Discovery |
| LZIC | Wald ratio | 1 | 2.3068 | 1.4084 | 3.7786 | 9.00E-04 | NA | NA | Discovery |
| MANEA | Inverse variance weighted | 3 | 0.9754 | 0.9739 | 0.9768 | 5.69E-235 | NA | NA | Discovery |
| MAPK3 | Wald ratio | 1 | 0.8873 | 0.8009 | 0.9831 | 2.22E-02 | NA | NA | Discovery |
| MAPK9 | Inverse variance weighted | 2 | 1.1720 | 1.0647 | 1.2902 | 1.20E-03 | NA | NA | Discovery |
| MATN2 | Inverse variance weighted | 4 | 0.8692 | 0.7699 | 0.9814 | 2.36E-02 | 0.4997 | 0.4346 | Discovery |
| MAX | Wald ratio | 1 | 0.8631 | 0.7582 | 0.9825 | 2.60E-02 | NA | NA | Discovery |
| MIF | Wald ratio | 1 | 0.6809 | 0.4705 | 0.9854 | 4.16E-02 | NA | NA | Discovery |
| MMP1 | Inverse variance weighted | 7 | 1.0720 | 1.0382 | 1.1069 | 2.11E-05 | 0.9152 | 0.9712 | Discovery |
| MMP8 | Inverse variance weighted | 3 | 1.0814 | 1.0324 | 1.1329 | 9.53E-04 | NA | NA | Discovery |
| MPO | Weighted median | 3 | 0.8510 | 0.7487 | 0.9673 | 1.36E-02 | NA | NA | Discovery |
| MRE11A | Wald ratio | 1 | 2.0299 | 1.0810 | 3.8119 | 2.77E-02 | NA | NA | Discovery |
| MSMB | Inverse variance weighted | 3 | 0.9440 | 0.9156 | 0.9734 | 2.29E-04 | NA | NA | Discovery |
| MTHFSD | Inverse variance weighted | 2 | 0.9468 | 0.9419 | 0.9517 | 4.28E-95 | NA | NA | Discovery |
| MTRF1L | Inverse variance weighted | 2 | 0.9851 | 0.9812 | 0.9889 | 4.04E-14 | NA | NA | Discovery |
| MVD | Inverse variance weighted | 2 | 0.5961 | 0.4893 | 0.7263 | 2.83E-07 | NA | NA | Discovery |
| NCAM2 | Inverse variance weighted | 8 | 0.9510 | 0.9085 | 0.9954 | 3.10E-02 | 0.8562 | 0.9203 | Discovery |
| NEO1 | Inverse variance weighted | 2 | 1.0770 | 1.0504 | 1.1042 | 6.17E-09 | NA | NA | Discovery |
| NPNT | Wald ratio | 1 | 0.7539 | 0.6521 | 0.8716 | 1.35E-04 | NA | NA | Discovery |
| NPPB | Inverse variance weighted | 3 | 1.1128 | 1.0333 | 1.1984 | 4.71E-03 | NA | NA | Discovery |
| NPPB | Weighted median | 3 | 1.1199 | 1.0003 | 1.2538 | 4.93E-02 | NA | NA | Discovery |
| NRCAM | Wald ratio | 1 | 0.6197 | 0.4391 | 0.8744 | 6.46E-03 | NA | NA | Discovery |
| NTF3 | Inverse variance weighted | 3 | 1.1953 | 1.0433 | 1.3694 | 1.01E-02 | NA | NA | Discovery |
| NTN4 | Inverse variance weighted | 4 | 0.9441 | 0.9244 | 0.9642 | 8.51E-08 | 0.9873 | 0.8109 | Discovery |
| NTRK2 | Wald ratio | 1 | 1.6917 | 1.1624 | 2.4619 | 6.03E-03 | NA | NA | Discovery |
| NTRK3 | Inverse variance weighted | 3 | 1.0945 | 1.0157 | 1.1795 | 1.79E-02 | NA | NA | Discovery |
| PCDH10 | Inverse variance weighted | 2 | 0.8996 | 0.8823 | 0.9172 | 9.06E-27 | NA | NA | Discovery |
| PCSK7 | Inverse variance weighted | 2 | 0.9464 | 0.9400 | 0.9529 | 3.40E-56 | NA | NA | Discovery |
| PCSK9 | Inverse variance weighted | 3 | 0.8949 | 0.8286 | 0.9664 | 4.65E-03 | NA | NA | Discovery |
| PDGFRL | Inverse variance weighted | 4 | 0.9700 | 0.9534 | 0.9868 | 5.02E-04 | 0.9629 | 0.7768 | Discovery |
| PEAR1 | Inverse variance weighted | 5 | 0.9394 | 0.9238 | 0.9553 | 2.58E-13 | 0.9956 | 0.8184 | Discovery |
| PFKM | Wald ratio | 1 | 1.2548 | 1.0633 | 1.4809 | 7.22E-03 | NA | NA | Discovery |
| PHPT1 | Inverse variance weighted | 2 | 0.9038 | 0.8287 | 0.9857 | 2.23E-02 | NA | NA | Discovery |
| PIGR | Inverse variance weighted | 2 | 0.9682 | 0.9415 | 0.9956 | 2.34E-02 | NA | NA | Discovery |
| PLA2R1 | Inverse variance weighted | 2 | 1.1432 | 1.0933 | 1.1953 | 4.02E-09 | NA | NA | Discovery |
| PLAU | Inverse variance weighted | 2 | 0.9648 | 0.9592 | 0.9705 | 1.44E-33 | NA | NA | Discovery |
| PLEKHA1 | Inverse variance weighted | 4 | 0.8946 | 0.8246 | 0.9706 | 7.43E-03 | 0.3983 | 0.4134 | Discovery |
| PLEKHA1 | Weighted median | 4 | 0.9072 | 0.8358 | 0.9846 | 1.98E-02 | 0.3983 | 0.4134 | Discovery |
| PLXNB2 | Weighted median | 3 | 1.0724 | 1.0017 | 1.1480 | 4.45E-02 | NA | NA | Discovery |
| PPIC | Wald ratio | 1 | 0.5699 | 0.3827 | 0.8485 | 5.62E-03 | NA | NA | Discovery |
| PPP1R14A | Inverse variance weighted | 2 | 1.1162 | 1.0727 | 1.1614 | 5.86E-08 | NA | NA | Discovery |
| PRDX1 | Inverse variance weighted | 3 | 0.8938 | 0.8027 | 0.9954 | 4.09E-02 | NA | NA | Discovery |
| PROC | Wald ratio | 1 | 1.2144 | 1.0195 | 1.4467 | 2.96E-02 | NA | NA | Discovery |
| PRSS27 | Inverse variance weighted | 2 | 0.7814 | 0.7195 | 0.8485 | 4.47E-09 | NA | NA | Discovery |
| PRSS8 | Wald ratio | 1 | 1.8714 | 1.2040 | 2.9086 | 5.35E-03 | NA | NA | Discovery |
| PSAP | Inverse variance weighted | 3 | 0.9404 | 0.8894 | 0.9943 | 3.07E-02 | NA | NA | Discovery |
| PSAPL1 | Inverse variance weighted | 2 | 1.0683 | 1.0413 | 1.0961 | 4.48E-07 | NA | NA | Discovery |
| PSG3 | Inverse variance weighted | 2 | 0.8024 | 0.7928 | 0.8122 | 3.35E-278 | NA | NA | Discovery |
| PTGFRN | Inverse variance weighted | 3 | 0.9506 | 0.9260 | 0.9760 | 1.60E-04 | NA | NA | Discovery |
| PTHLH | Inverse variance weighted | 2 | 1.0358 | 1.0218 | 1.0500 | 3.94E-07 | NA | NA | Discovery |
| PYGL | Inverse variance weighted | 3 | 0.9306 | 0.8839 | 0.9798 | 6.18E-03 | NA | NA | Discovery |
| QPCT | Inverse variance weighted | 5 | 0.9208 | 0.8873 | 0.9555 | 1.24E-05 | 0.9222 | 0.5764 | Discovery |
| RAB22A | Inverse variance weighted | 2 | 0.8819 | 0.8235 | 0.9443 | 3.19E-04 | NA | NA | Discovery |
| RBP1 | Wald ratio | 1 | 1.5085 | 1.1024 | 2.0641 | 1.02E-02 | NA | NA | Discovery |
| RBP5 | Inverse variance weighted | 3 | 0.9295 | 0.8853 | 0.9759 | 3.26E-03 | NA | NA | Discovery |
| RBP7 | Inverse variance weighted | 2 | 1.2290 | 1.1348 | 1.3309 | 3.98E-07 | NA | NA | Discovery |
| RCN1 | Wald ratio | 1 | 0.8211 | 0.6931 | 0.9726 | 2.25E-02 | NA | NA | Discovery |
| REG3G | Inverse variance weighted | 2 | 0.9631 | 0.9596 | 0.9666 | 2.93E-91 | NA | NA | Discovery |
| RELT | Wald ratio | 1 | 0.5534 | 0.3424 | 0.8945 | 1.57E-02 | NA | NA | Discovery |
| RFESD | Wald ratio | 1 | 0.6542 | 0.4287 | 0.9983 | 4.91E-02 | NA | NA | Discovery |
| RGMA | Inverse variance weighted | 2 | 0.7507 | 0.6436 | 0.8756 | 2.60E-04 | NA | NA | Discovery |
| ROR1 | Inverse variance weighted | 6 | 0.9593 | 0.9335 | 0.9858 | 2.77E-03 | 0.9308 | 0.6541 | Discovery |
| RRM1 | Wald ratio | 1 | 0.6681 | 0.4604 | 0.9695 | 3.38E-02 | NA | NA | Discovery |
| RSPO1 | Inverse variance weighted | 3 | 0.9607 | 0.9325 | 0.9897 | 8.33E-03 | NA | NA | Discovery |
| S100A2 | Inverse variance weighted | 2 | 1.1696 | 1.1198 | 1.2217 | 1.79E-12 | NA | NA | Discovery |
| SBDS | Wald ratio | 1 | 1.3373 | 1.0583 | 1.6898 | 1.49E-02 | NA | NA | Discovery |
| SCARF2 | Inverse variance weighted | 2 | 0.7431 | 0.6253 | 0.8831 | 7.47E-04 | NA | NA | Discovery |
| SCG3 | Inverse variance weighted | 2 | 1.1111 | 1.1079 | 1.1143 | 0.00E+00 | NA | NA | Discovery |
| SCGB3A1 | Inverse variance weighted | 2 | 1.0648 | 1.0410 | 1.0892 | 5.40E-08 | NA | NA | Discovery |
| SEMA6A | Inverse variance weighted | 2 | 1.3209 | 1.1973 | 1.4572 | 2.81E-08 | NA | NA | Discovery |
| SEPW1 | Inverse variance weighted | 2 | 0.9437 | 0.8983 | 0.9915 | 2.14E-02 | NA | NA | Discovery |
| SERPINA12 | Inverse variance weighted | 2 | 0.9704 | 0.9419 | 0.9999 | 4.90E-02 | NA | NA | Discovery |
| SERPINA3 | Inverse variance weighted | 3 | 0.8798 | 0.8522 | 0.9083 | 3.51E-15 | NA | NA | Discovery |
| SERPINF1 | Weighted median | 4 | 1.0984 | 1.0281 | 1.1736 | 5.45E-03 | 0.1615 | 0.2998 | Discovery |
| SLITRK3 | Weighted median | 3 | 0.9312 | 0.8694 | 0.9974 | 4.19E-02 | NA | NA | Discovery |
| SMOC2 | Inverse variance weighted | 5 | 1.0180 | 1.0020 | 1.0343 | 2.71E-02 | 0.9852 | 0.9637 | Discovery |
| SMPDL3A | Inverse variance weighted | 2 | 0.8899 | 0.8851 | 0.8947 | 0.00E+00 | NA | NA | Discovery |
| SPINK2 | Inverse variance weighted | 4 | 1.0503 | 1.0342 | 1.0667 | 5.06E-10 | 0.9783 | 0.8208 | Discovery |
| SPINK7 | Wald ratio | 1 | 0.7903 | 0.6535 | 0.9556 | 1.52E-02 | NA | NA | Discovery |
| SPOCK3 | Inverse variance weighted | 2 | 1.1323 | 1.0639 | 1.2052 | 9.40E-05 | NA | NA | Discovery |
| SRA1 | Inverse variance weighted | 3 | 1.0834 | 1.0005 | 1.1732 | 4.84E-02 | NA | NA | Discovery |
| SRI | Wald ratio | 1 | 1.4181 | 1.1256 | 1.7865 | 3.04E-03 | NA | NA | Discovery |
| ST8SIA6 | Wald ratio | 1 | 0.5690 | 0.3388 | 0.9558 | 3.31E-02 | NA | NA | Discovery |
| SWAP70 | Inverse variance weighted | 2 | 1.0263 | 1.0091 | 1.0438 | 2.68E-03 | NA | NA | Discovery |
| TAGLN2 | Wald ratio | 1 | 0.7600 | 0.5878 | 0.9827 | 3.63E-02 | NA | NA | Discovery |
| TESC | Inverse variance weighted | 2 | 1.2925 | 1.1295 | 1.4791 | 1.92E-04 | NA | NA | Discovery |
| TFF1 | Wald ratio | 1 | 0.7487 | 0.5962 | 0.9401 | 1.27E-02 | NA | NA | Discovery |
| THBS3 | Wald ratio | 1 | 1.5188 | 1.0980 | 2.1009 | 1.16E-02 | NA | NA | Discovery |
| TIMP2 | Inverse variance weighted | 2 | 0.7727 | 0.7646 | 0.7810 | 0.00E+00 | NA | NA | Discovery |
| TIMP4 | Inverse variance weighted | 3 | 0.9456 | 0.9291 | 0.9625 | 5.67E-10 | NA | NA | Discovery |
| TMEM106A | Wald ratio | 1 | 0.6106 | 0.3941 | 0.9459 | 2.72E-02 | NA | NA | Discovery |
| TMEM132C | Inverse variance weighted | 3 | 1.0473 | 1.0142 | 1.0815 | 4.84E-03 | NA | NA | Discovery |
| TNFSF14 | Inverse variance weighted | 2 | 0.9460 | 0.9292 | 0.9631 | 1.22E-09 | NA | NA | Discovery |
| TP53I3 | Inverse variance weighted | 2 | 1.0954 | 1.0079 | 1.1905 | 3.19E-02 | NA | NA | Discovery |
| UAP1 | Wald ratio | 1 | 2.2167 | 1.3058 | 3.7629 | 3.20E-03 | NA | NA | Discovery |
| UGDH | Inverse variance weighted | 2 | 0.9276 | 0.8918 | 0.9649 | 1.85E-04 | NA | NA | Discovery |
| UROS | Weighted median | 3 | 1.1045 | 1.0114 | 1.2062 | 2.69E-02 | NA | NA | Discovery |
| VASN | Inverse variance weighted | 2 | 0.7466 | 0.6844 | 0.8144 | 4.43E-11 | NA | NA | Discovery |
| VEGFA | Inverse variance weighted | 5 | 1.0533 | 1.0070 | 1.1018 | 2.36E-02 | 0.8911 | 0.9791 | Discovery |
| VEGFC | Wald ratio | 1 | 1.2570 | 1.0152 | 1.5564 | 3.59E-02 | NA | NA | Discovery |
| VOPP1 | Inverse variance weighted | 2 | 1.1700 | 1.1381 | 1.2027 | 7.85E-29 | NA | NA | Discovery |
| VTA1 | Wald ratio | 1 | 0.4621 | 0.2887 | 0.7396 | 1.30E-03 | NA | NA | Discovery |
| VWC2 | Inverse variance weighted | 3 | 0.8345 | 0.7427 | 0.9378 | 2.36E-03 | NA | NA | Discovery |
| VWC2 | Weighted median | 3 | 0.8403 | 0.7227 | 0.9772 | 2.39E-02 | NA | NA | Discovery |
| WFDC1 | Inverse variance weighted | 3 | 1.0832 | 1.0375 | 1.1310 | 2.82E-04 | NA | NA | Discovery |
| ZYX | Inverse variance weighted | 2 | 0.7909 | 0.6937 | 0.9017 | 4.53E-04 | NA | NA | Discovery |
| ADAM19 | Wald ratio | 1 | 0.8291 | 0.7511 | 0.9152 | 2.01E-04 | NA | NA | Validation |
| ADAM22 | Wald ratio | 1 | 0.8702 | 0.7679 | 0.9862 | 2.95E-02 | NA | NA | Validation |
| AKR1B1 | Wald ratio | 1 | 1.1752 | 1.0256 | 1.3466 | 2.02E-02 | NA | NA | Validation |
| APOA5 | Wald ratio | 1 | 1.1311 | 1.0092 | 1.2677 | 3.42E-02 | NA | NA | Validation |
| ASIP | Wald ratio | 1 | 1.0658 | 1.0152 | 1.1190 | 1.02E-02 | NA | NA | Validation |
| B3GAT3 | Wald ratio | 1 | 1.0780 | 1.0011 | 1.1609 | 4.68E-02 | NA | NA | Validation |
| CA10 | Wald ratio | 1 | 0.8648 | 0.7779 | 0.9614 | 7.18E-03 | NA | NA | Validation |
| CCL27 | Wald ratio | 1 | 1.1448 | 1.0058 | 1.3030 | 4.06E-02 | NA | NA | Validation |
| CD177 | Wald ratio | 1 | 1.0457 | 1.0001 | 1.0933 | 4.96E-02 | NA | NA | Validation |
| CLMP | Wald ratio | 1 | 1.1326 | 1.0346 | 1.2399 | 7.03E-03 | NA | NA | Validation |
| COLEC12 | Wald ratio | 1 | 1.1886 | 1.0276 | 1.3748 | 2.00E-02 | NA | NA | Validation |
| CTGF | Wald ratio | 1 | 0.9105 | 0.8471 | 0.9786 | 1.09E-02 | NA | NA | Validation |
| CTSB | Wald ratio | 1 | 0.9130 | 0.8519 | 0.9784 | 9.88E-03 | NA | NA | Validation |
| CXCL16 | Wald ratio | 1 | 1.2122 | 1.0619 | 1.3838 | 4.39E-03 | NA | NA | Validation |
| DKK1 | Wald ratio | 1 | 0.8388 | 0.7315 | 0.9617 | 1.17E-02 | NA | NA | Validation |
| DLK1 | Wald ratio | 1 | 0.9415 | 0.8962 | 0.9892 | 1.67E-02 | NA | NA | Validation |
| DSG2 | Wald ratio | 1 | 1.1594 | 1.0147 | 1.3249 | 2.97E-02 | NA | NA | Validation |
| DUSP13 | Wald ratio | 1 | 0.9186 | 0.8468 | 0.9963 | 4.05E-02 | NA | NA | Validation |
| ENPP5 | Wald ratio | 1 | 1.0342 | 1.0043 | 1.0649 | 2.47E-02 | NA | NA | Validation |
| ERAP2 | Wald ratio | 1 | 0.9676 | 0.9475 | 0.9882 | 2.20E-03 | NA | NA | Validation |
| FAM151A | Wald ratio | 1 | 1.1027 | 1.0105 | 1.2032 | 2.82E-02 | NA | NA | Validation |
| FAM20A | Wald ratio | 1 | 0.9100 | 0.8285 | 0.9994 | 4.86E-02 | NA | NA | Validation |
| FCGR3B | Wald ratio | 1 | 1.1285 | 1.0662 | 1.1945 | 2.99E-05 | NA | NA | Validation |
| FCN2 | Wald ratio | 1 | 1.0482 | 1.0023 | 1.0962 | 3.92E-02 | NA | NA | Validation |
| GKN2 | Wald ratio | 1 | 0.9362 | 0.8823 | 0.9934 | 2.93E-02 | NA | NA | Validation |
| GSTO1 | Wald ratio | 1 | 1.0262 | 1.0001 | 1.0531 | 4.92E-02 | NA | NA | Validation |
| HPX | Wald ratio | 1 | 1.1331 | 1.0139 | 1.2665 | 2.76E-02 | NA | NA | Validation |
| IGFBP5 | Wald ratio | 1 | 0.6419 | 0.4949 | 0.8327 | 8.39E-04 | NA | NA | Validation |
| IL18 | Wald ratio | 1 | 1.1511 | 1.0471 | 1.2653 | 3.57E-03 | NA | NA | Validation |
| KDELC2 | Wald ratio | 1 | 1.1089 | 1.0366 | 1.1862 | 2.66E-03 | NA | NA | Validation |
| KLK8 | Wald ratio | 1 | 1.0845 | 1.0082 | 1.1667 | 2.93E-02 | NA | NA | Validation |
| LHB | Wald ratio | 1 | 0.8731 | 0.7962 | 0.9574 | 3.89E-03 | NA | NA | Validation |
| MAN1A2 | Wald ratio | 1 | 0.8656 | 0.7689 | 0.9746 | 1.70E-02 | NA | NA | Validation |
| MRC2 | Wald ratio | 1 | 1.2422 | 1.0746 | 1.4360 | 3.37E-03 | NA | NA | Validation |
| NAGPA | Wald ratio | 1 | 1.0726 | 1.0049 | 1.1448 | 3.50E-02 | NA | NA | Validation |
| PCSK9 | Wald ratio | 1 | 0.8824 | 0.7965 | 0.9774 | 1.65E-02 | NA | NA | Validation |
| PLEKHA7 | Wald ratio | 1 | 0.8839 | 0.7831 | 0.9975 | 4.55E-02 | NA | NA | Validation |
| PLG | Wald ratio | 1 | 1.0865 | 1.0125 | 1.1659 | 2.11E-02 | NA | NA | Validation |
| PTGFRN | Wald ratio | 1 | 0.9618 | 0.9345 | 0.9899 | 7.98E-03 | NA | NA | Validation |
| QDPR | Wald ratio | 1 | 1.0497 | 1.0008 | 1.1010 | 4.63E-02 | NA | NA | Validation |
| RELT | Wald ratio | 1 | 0.9315 | 0.8683 | 0.9993 | 4.77E-02 | NA | NA | Validation |
| RPN1 | Wald ratio | 1 | 0.9090 | 0.8487 | 0.9737 | 6.55E-03 | NA | NA | Validation |
| RSPO3 | Wald ratio | 1 | 0.9149 | 0.8429 | 0.9932 | 3.37E-02 | NA | NA | Validation |
| SCARF2 | Wald ratio | 1 | 0.8190 | 0.7440 | 0.9016 | 4.60E-05 | NA | NA | Validation |
| SERPINE2 | Wald ratio | 1 | 0.8748 | 0.8142 | 0.9399 | 2.58E-04 | NA | NA | Validation |
| SERPINF1 | Wald ratio | 1 | 1.0587 | 1.0051 | 1.1152 | 3.14E-02 | NA | NA | Validation |
| SIGLEC9 | Wald ratio | 1 | 0.9759 | 0.9583 | 0.9939 | 8.72E-03 | NA | NA | Validation |
| SIRPA | Wald ratio | 1 | 0.9713 | 0.9529 | 0.9900 | 2.78E-03 | NA | NA | Validation |
| SLITRK3 | Wald ratio | 1 | 0.8965 | 0.8107 | 0.9913 | 3.32E-02 | NA | NA | Validation |
| SOD3 | Wald ratio | 1 | 1.0680 | 1.0134 | 1.1255 | 1.40E-02 | NA | NA | Validation |
| SPARCL1 | Inverse variance weighted | 2 | 0.9683 | 0.9497 | 0.9871 | 1.06E-03 | NA | NA | Validation |
| SPINT2 | Wald ratio | 1 | 1.0253 | 1.0009 | 1.0504 | 4.21E-02 | NA | NA | Validation |
| THSD1 | Wald ratio | 1 | 0.8963 | 0.8176 | 0.9826 | 1.96E-02 | NA | NA | Validation |
| TNFRSF6B | Wald ratio | 1 | 0.7974 | 0.6653 | 0.9558 | 1.43E-02 | NA | NA | Validation |
| UXS1 | Wald ratio | 1 | 1.2320 | 1.0792 | 1.4065 | 2.02E-03 | NA | NA | Validation |
| VEGFC | Wald ratio | 1 | 1.0539 | 1.0035 | 1.1069 | 3.59E-02 | NA | NA | Validation |

**Notes:** “Proteins” column refers to the name of a protein; “Methods” column refers to the statistical method used to calculated the causal effect; “NSNP” column refers to the number of single nucleotide polymorphism used in causal estimates; “OR” column refers to the odds ratio; “Lower of 95%CI” column refers to the lower limit of 95% confidence interval; “Upper of 95%CI” refers to the upper limit of 95% confidence interval; “P” column refers to the p-value of odds ratio; “P_het_” column refers to the p-value of heterogeneity test; “P_pleio_” column refers to the p-value of horizontal pleiotropy test; “Stage” column refers to the “Discovery” or “Validation” stage.

**Supplementary Table 4: The results of enrichment analyses using circulating proteins closely associated with chronic obstructive pulmonary disease.**

| **Gene Set** | **Description** | **Size** | **Expect** | **Ratio** | **P** | **Database** |
| --- | --- | --- | --- | --- | --- | --- |
| R-HSA-597592 | Post-translational protein modification | 1425 | 0.38196 | 7.85420 | 3.23E-03 | Reactome |
| R-HSA-381426 | Regulation of Insulin-like Growth Factor (IGF) transport and uptake by Insulin-like Growth Factor Binding Proteins (IGFBPs) | 125 | 0.03351 | 59.69200 | 4.13E-04 | Reactome |
| R-HSA-8957275 | Post-translational protein phosphorylation | 108 | 0.02895 | 69.08800 | 3.08E-04 | Reactome |
| R-HSA-174824 | Plasma lipoprotein assembly, remodeling, and clearance | 69 | 0.01850 | 108.14000 | 1.26E-04 | Reactome |
| R-HSA-209952 | Peptide hormone biosynthesis | 14 | 0.00375 | 266.48000 | 3.75E-03 | Reactome |
| R-HSA-8866427 | VLDLR internalisation and degradation | 12 | 0.00322 | 310.90000 | 3.21E-03 | Reactome |
| R-HSA-209822 | Glycoprotein hormones | 12 | 0.00322 | 310.90000 | 3.21E-03 | Reactome |
| R-HSA-375281 | Hormone ligand-binding receptors | 12 | 0.00322 | 310.90000 | 3.21E-03 | Reactome |
| R-HSA-351906 | Apoptotic cleavage of cell adhesion proteins | 11 | 0.00295 | 339.16000 | 2.95E-03 | Reactome |
| R-HSA-193048 | Androgen biosynthesis | 11 | 0.00295 | 339.16000 | 2.95E-03 | Reactome |
| R-HSA-975578 | Reactions specific to the complex N-glycan synthesis pathway | 10 | 0.00268 | 373.07000 | 2.68E-03 | Reactome |
| R-HSA-8963901 | Chylomicron remodeling | 9 | 0.00241 | 414.53000 | 2.41E-03 | Reactome |
| R-HSA-193993 | Mineralocorticoid biosynthesis | 6 | 0.00161 | 621.79000 | 1.61E-03 | Reactome |
| C1956346 | Coronary Artery Disease | 78 | 0.02091 | 95.66000 | 1.61E-04 | DisGeNET |
| C0020672 | Hypothermia, natural | 17 | 0.00456 | 219.46000 | 4.55E-03 | DisGeNET |
| C0020557 | Hypertriglyceridemia | 12 | 0.00322 | 310.90000 | 3.21E-03 | DisGeNET |
| C0745103 | Hyperlipoproteinemia Type IIa | 11 | 0.00295 | 339.16000 | 2.95E-03 | DisGeNET |
| C0020514 | Hyperprolactinemia | 10 | 0.00268 | 373.07000 | 2.68E-03 | DisGeNET |
| C2063326 | Cardiomyopathy, right ventricular | 7 | 0.00188 | 532.96000 | 1.88E-03 | DisGeNET |
| C0151636 | Premature ventricular contractions | 6 | 0.00161 | 621.79000 | 1.61E-03 | DisGeNET |

**Supplementary Table 5: Associations of circulation proteins with leukocyte telomere length.**

| **Proteins** | **Methods** | **NSNP** | **OR** | **Lower of 95%CI** | **Upper of 95%CI** | **P** | **P_het_** | **P_pleio_** | **Stage** |
| --- | --- | --- | --- | --- | --- | --- | --- | --- | --- |
| ACADSB | Inverse variance weighted | 2 | 1.0244 | 1.0231 | 1.0258 | 1.85E-290 | NA | NA | Discovery |
| ACE | Inverse variance weighted | 2 | 1.0062 | 1.0043 | 1.0081 | 7.93E-11 | NA | NA | Discovery |
| ACYP2 | Wald ratio | 1 | 0.9119 | 0.8449 | 0.9843 | 1.80E-02 | NA | NA | Discovery |
| ADAMTS13 | Inverse variance weighted | 3 | 1.0102 | 1.0078 | 1.0126 | 9.43E-17 | NA | NA | Discovery |
| ADAMTSL2 | Inverse variance weighted | 3 | 0.9917 | 0.9850 | 0.9985 | 1.64E-02 | NA | NA | Discovery |
| ADH1A | Wald ratio | 1 | 1.0428 | 1.0160 | 1.0702 | 1.59E-03 | NA | NA | Discovery |
| AES | Wald ratio | 1 | 0.8833 | 0.8131 | 0.9595 | 3.30E-03 | NA | NA | Discovery |
| AGER | Weighted median | 3 | 0.9762 | 0.9655 | 0.9870 | 1.66E-05 | NA | NA | Discovery |
| AGRN | Inverse variance weighted | 4 | 1.0113 | 1.0005 | 1.0223 | 4.08E-02 | 0.4512 | 0.2977 | Discovery |
| AGRP | Inverse variance weighted | 2 | 1.0132 | 1.0058 | 1.0206 | 4.34E-04 | NA | NA | Discovery |
| AKR7A3 | Inverse variance weighted | 4 | 1.0147 | 1.0116 | 1.0177 | 1.28E-21 | 0.9705 | 0.8239 | Discovery |
| AKR7A3 | Weighted median | 4 | 1.0137 | 1.0017 | 1.0257 | 2.49E-02 | 0.9705 | 0.8239 | Discovery |
| ALDH2 | Wald ratio | 1 | 0.8477 | 0.7986 | 0.8999 | 5.93E-08 | NA | NA | Discovery |
| AMIGO2 | Inverse variance weighted | 3 | 0.9848 | 0.9747 | 0.9950 | 3.64E-03 | NA | NA | Discovery |
| AMY2A | Wald ratio | 1 | 1.1242 | 1.0119 | 1.2490 | 2.92E-02 | NA | NA | Discovery |
| ANTXR2 | Inverse variance weighted | 3 | 1.0023 | 1.0010 | 1.0037 | 5.47E-04 | NA | NA | Discovery |
| ANXA1 | Inverse variance weighted | 2 | 0.9826 | 0.9798 | 0.9854 | 7.12E-33 | NA | NA | Discovery |
| ANXA11 | Inverse variance weighted | 3 | 1.0095 | 1.0010 | 1.0182 | 2.92E-02 | NA | NA | Discovery |
| ANXA2 | Inverse variance weighted | 6 | 0.9944 | 0.9903 | 0.9985 | 6.93E-03 | 0.9617 | 0.8241 | Discovery |
| APOA5 | Wald ratio | 1 | 0.9606 | 0.9417 | 0.9799 | 7.50E-05 | NA | NA | Discovery |
| ARG1 | Inverse variance weighted | 2 | 0.9866 | 0.9841 | 0.9891 | 4.50E-25 | NA | NA | Discovery |
| ARHGEF25 | Inverse variance weighted | 2 | 1.0153 | 1.0099 | 1.0206 | 1.77E-08 | NA | NA | Discovery |
| ARRB1 | Wald ratio | 1 | 0.9307 | 0.8819 | 0.9821 | 8.84E-03 | NA | NA | Discovery |
| ARSB | Inverse variance weighted | 2 | 1.0159 | 1.0008 | 1.0312 | 3.84E-02 | NA | NA | Discovery |
| ASF1A | Inverse variance weighted | 2 | 0.9812 | 0.9803 | 0.9821 | 0.00E+00 | NA | NA | Discovery |
| ASPN | Inverse variance weighted | 4 | 0.9923 | 0.9886 | 0.9961 | 6.16E-05 | 0.9462 | 0.9168 | Discovery |
| ATF6B | Wald ratio | 1 | 1.0836 | 1.0605 | 1.1072 | 2.91E-13 | NA | NA | Discovery |
| AZGP1 | Inverse variance weighted | 2 | 1.0012 | 1.0001 | 1.0024 | 3.86E-02 | NA | NA | Discovery |
| B4GALT1 | Inverse variance weighted | 2 | 1.0171 | 1.0133 | 1.0209 | 5.78E-19 | NA | NA | Discovery |
| BGN | Inverse variance weighted | 2 | 1.0121 | 1.0064 | 1.0178 | 2.79E-05 | NA | NA | Discovery |
| BPIFA2 | Inverse variance weighted | 2 | 0.9879 | 0.9760 | 0.9999 | 4.75E-02 | NA | NA | Discovery |
| BPIFB1 | Inverse variance weighted | 4 | 0.9895 | 0.9802 | 0.9990 | 3.02E-02 | 0.6837 | 0.4466 | Discovery |
| BPNT1 | Wald ratio | 1 | 1.0789 | 1.0028 | 1.1607 | 4.18E-02 | NA | NA | Discovery |
| BTC | Inverse variance weighted | 2 | 1.0043 | 1.0007 | 1.0078 | 1.81E-02 | NA | NA | Discovery |
| C1GALT1C1 | Inverse variance weighted | 2 | 0.9881 | 0.9823 | 0.9939 | 5.72E-05 | NA | NA | Discovery |
| C1QTNF5 | Inverse variance weighted | 2 | 1.0023 | 1.0015 | 1.0030 | 7.25E-10 | NA | NA | Discovery |
| C1QTNF9 | Inverse variance weighted | 2 | 0.9897 | 0.9886 | 0.9909 | 8.13E-67 | NA | NA | Discovery |
| C2 | Inverse variance weighted | 3 | 0.9139 | 0.8468 | 0.9863 | 2.06E-02 | NA | NA | Discovery |
| C2 | Weighted median | 3 | 0.9115 | 0.8875 | 0.9361 | 1.03E-11 | NA | NA | Discovery |
| C5 | Inverse variance weighted | 4 | 1.0094 | 1.0062 | 1.0125 | 4.49E-09 | 0.9948 | 0.8869 | Discovery |
| CA1 | Inverse variance weighted | 2 | 0.9583 | 0.9383 | 0.9787 | 7.33E-05 | NA | NA | Discovery |
| CA3 | Inverse variance weighted | 3 | 1.0232 | 1.0062 | 1.0405 | 7.25E-03 | NA | NA | Discovery |
| CA3 | Weighted median | 3 | 1.0259 | 1.0039 | 1.0483 | 2.06E-02 | NA | NA | Discovery |
| CALCB | Inverse variance weighted | 2 | 0.9914 | 0.9850 | 0.9980 | 1.02E-02 | NA | NA | Discovery |
| CAMP | Wald ratio | 1 | 1.1272 | 1.0733 | 1.1838 | 1.70E-06 | NA | NA | Discovery |
| CAT | Inverse variance weighted | 2 | 0.9787 | 0.9682 | 0.9893 | 8.60E-05 | NA | NA | Discovery |
| CBL | Wald ratio | 1 | 0.9639 | 0.9373 | 0.9912 | 9.92E-03 | NA | NA | Discovery |
| CCDC126 | Weighted median | 3 | 0.9781 | 0.9676 | 0.9888 | 6.26E-05 | NA | NA | Discovery |
| CCL11 | Inverse variance weighted | 2 | 0.9747 | 0.9503 | 0.9997 | 4.72E-02 | NA | NA | Discovery |
| CCL15 | Inverse variance weighted | 2 | 0.9869 | 0.9794 | 0.9945 | 7.30E-04 | NA | NA | Discovery |
| CCL3 | Inverse variance weighted | 2 | 1.0078 | 1.0013 | 1.0145 | 1.95E-02 | NA | NA | Discovery |
| CCL4L1 | Inverse variance weighted | 2 | 1.0520 | 1.0241 | 1.0805 | 2.12E-04 | NA | NA | Discovery |
| CCS | Wald ratio | 1 | 0.9579 | 0.9241 | 0.9928 | 1.87E-02 | NA | NA | Discovery |
| CD109 | Inverse variance weighted | 3 | 0.9903 | 0.9827 | 0.9979 | 1.22E-02 | NA | NA | Discovery |
| CD33 | Inverse variance weighted | 4 | 0.9937 | 0.9887 | 0.9988 | 1.46E-02 | 0.6886 | 0.6274 | Discovery |
| CD46 | Inverse variance weighted | 2 | 1.0300 | 1.0179 | 1.0423 | 1.02E-06 | NA | NA | Discovery |
| CD58 | Inverse variance weighted | 2 | 0.9825 | 0.9686 | 0.9967 | 1.57E-02 | NA | NA | Discovery |
| CD68 | Wald ratio | 1 | 0.9010 | 0.8434 | 0.9626 | 1.99E-03 | NA | NA | Discovery |
| CD72 | Inverse variance weighted | 2 | 1.0445 | 1.0400 | 1.0490 | 5.84E-87 | NA | NA | Discovery |
| CDNF | Inverse variance weighted | 3 | 1.0145 | 1.0117 | 1.0174 | 1.35E-23 | NA | NA | Discovery |
| CDNF | Weighted median | 3 | 1.0144 | 1.0015 | 1.0275 | 2.87E-02 | NA | NA | Discovery |
| CDON | Inverse variance weighted | 2 | 0.9810 | 0.9670 | 0.9953 | 9.11E-03 | NA | NA | Discovery |
| CEL | Weighted median | 6 | 0.9875 | 0.9763 | 0.9988 | 3.02E-02 | 0.1028 | 0.7081 | Discovery |
| CFL2 | Wald ratio | 1 | 1.0435 | 1.0067 | 1.0817 | 2.02E-02 | NA | NA | Discovery |
| CHI3L1 | Inverse variance weighted | 2 | 0.9837 | 0.9756 | 0.9919 | 1.05E-04 | NA | NA | Discovery |
| CHIT1 | Inverse variance weighted | 3 | 1.0095 | 1.0022 | 1.0169 | 1.03E-02 | NA | NA | Discovery |
| CHKB | Inverse variance weighted | 3 | 0.8752 | 0.7959 | 0.9625 | 6.00E-03 | NA | NA | Discovery |
| CHKB | Weighted median | 3 | 0.9028 | 0.8542 | 0.9542 | 2.95E-04 | NA | NA | Discovery |
| CHL1 | Inverse variance weighted | 6 | 0.9890 | 0.9851 | 0.9929 | 3.63E-08 | 0.9727 | 0.6271 | Discovery |
| CLPS | Weighted median | 8 | 0.9839 | 0.9716 | 0.9964 | 1.16E-02 | 0.0001 | 0.0678 | Discovery |
| CMPK1 | Inverse variance weighted | 2 | 0.9753 | 0.9721 | 0.9786 | 1.63E-48 | NA | NA | Discovery |
| CNPY3 | Wald ratio | 1 | 1.0379 | 1.0051 | 1.0717 | 2.30E-02 | NA | NA | Discovery |
| CNRIP1 | Wald ratio | 1 | 1.0388 | 1.0166 | 1.0615 | 5.63E-04 | NA | NA | Discovery |
| COL1A1 | Inverse variance weighted | 2 | 0.9451 | 0.9106 | 0.9810 | 2.95E-03 | NA | NA | Discovery |
| COLEC12 | Inverse variance weighted | 5 | 0.9912 | 0.9846 | 0.9978 | 9.18E-03 | 0.9766 | 0.8130 | Discovery |
| COMP | Inverse variance weighted | 3 | 0.9877 | 0.9827 | 0.9926 | 1.14E-06 | NA | NA | Discovery |
| CPLX1 | Inverse variance weighted | 2 | 0.9854 | 0.9817 | 0.9891 | 2.09E-14 | NA | NA | Discovery |
| CPQ | Inverse variance weighted | 5 | 0.9828 | 0.9666 | 0.9993 | 4.15E-02 | 0.4508 | 0.6767 | Discovery |
| CRAT | Inverse variance weighted | 2 | 1.0215 | 1.0037 | 1.0396 | 1.74E-02 | NA | NA | Discovery |
| CREB3L4 | Wald ratio | 1 | 1.0257 | 1.0058 | 1.0461 | 1.14E-02 | NA | NA | Discovery |
| CREG1 | Inverse variance weighted | 3 | 1.0100 | 1.0005 | 1.0196 | 3.85E-02 | NA | NA | Discovery |
| CRLF1 | Inverse variance weighted | 2 | 1.0167 | 1.0110 | 1.0224 | 6.89E-09 | NA | NA | Discovery |
| CRYBB1 | Inverse variance weighted | 2 | 1.0204 | 1.0034 | 1.0376 | 1.85E-02 | NA | NA | Discovery |
| CSNK2B | Wald ratio | 1 | 0.8920 | 0.8035 | 0.9902 | 3.19E-02 | NA | NA | Discovery |
| CST3 | Inverse variance weighted | 2 | 1.0113 | 1.0033 | 1.0194 | 5.55E-03 | NA | NA | Discovery |
| CST6 | Wald ratio | 1 | 1.0738 | 1.0033 | 1.1492 | 3.98E-02 | NA | NA | Discovery |
| CTRB1 | Wald ratio | 1 | 0.8625 | 0.7785 | 0.9556 | 4.68E-03 | NA | NA | Discovery |
| CXCL16 | Inverse variance weighted | 2 | 0.9899 | 0.9895 | 0.9903 | 0.00E+00 | NA | NA | Discovery |
| DARS | Wald ratio | 1 | 1.1187 | 1.0600 | 1.1807 | 4.59E-05 | NA | NA | Discovery |
| DDT | Wald ratio | 1 | 0.9663 | 0.9366 | 0.9970 | 3.15E-02 | NA | NA | Discovery |
| DEFB1 | Inverse variance weighted | 3 | 0.9807 | 0.9695 | 0.9920 | 8.59E-04 | NA | NA | Discovery |
| DEFB4A | Inverse variance weighted | 4 | 0.9861 | 0.9730 | 0.9994 | 4.07E-02 | 0.8076 | 0.7407 | Discovery |
| DLK1 | Weighted median | 4 | 0.9853 | 0.9737 | 0.9970 | 1.37E-02 | 0.2093 | 0.2038 | Discovery |
| DNAJB14 | Wald ratio | 1 | 0.9074 | 0.8466 | 0.9726 | 6.02E-03 | NA | NA | Discovery |
| DNAJB4 | Inverse variance weighted | 3 | 0.9990 | 0.9983 | 0.9997 | 4.35E-03 | NA | NA | Discovery |
| DPEP2 | Wald ratio | 1 | 1.0590 | 1.0260 | 1.0930 | 3.82E-04 | NA | NA | Discovery |
| DPT | Inverse variance weighted | 5 | 1.0166 | 1.0001 | 1.0333 | 4.86E-02 | 0.2056 | 0.2835 | Discovery |
| DPT | Weighted median | 5 | 1.0177 | 1.0032 | 1.0325 | 1.66E-02 | 0.2056 | 0.2835 | Discovery |
| DTYMK | Inverse variance weighted | 2 | 1.0253 | 1.0145 | 1.0362 | 3.59E-06 | NA | NA | Discovery |
| EIF1AD | Wald ratio | 1 | 1.0227 | 1.0019 | 1.0438 | 3.22E-02 | NA | NA | Discovery |
| ENG | Inverse variance weighted | 2 | 0.9974 | 0.9948 | 0.9999 | 4.24E-02 | NA | NA | Discovery |
| ENPEP | Inverse variance weighted | 6 | 0.9911 | 0.9831 | 0.9991 | 2.98E-02 | 0.5642 | 0.5432 | Discovery |
| ENTHD2 | Wald ratio | 1 | 1.1273 | 1.0356 | 1.2272 | 5.64E-03 | NA | NA | Discovery |
| ESM1 | Inverse variance weighted | 3 | 1.0081 | 1.0034 | 1.0128 | 7.11E-04 | NA | NA | Discovery |
| F13B | Wald ratio | 1 | 1.0484 | 1.0116 | 1.0865 | 9.57E-03 | NA | NA | Discovery |
| F5 | Weighted median | 3 | 1.0160 | 1.0001 | 1.0322 | 4.83E-02 | NA | NA | Discovery |
| F8 | Wald ratio | 1 | 1.2503 | 1.1731 | 1.3325 | 6.40E-12 | NA | NA | Discovery |
| FAM177A1 | Inverse variance weighted | 2 | 1.0078 | 1.0074 | 1.0083 | 9.17E-257 | NA | NA | Discovery |
| FAM3D | Inverse variance weighted | 2 | 0.9940 | 0.9918 | 0.9962 | 1.05E-07 | NA | NA | Discovery |
| FAP | Inverse variance weighted | 2 | 0.9785 | 0.9702 | 0.9869 | 6.65E-07 | NA | NA | Discovery |
| FCER2 | Inverse variance weighted | 6 | 1.0101 | 1.0040 | 1.0162 | 1.19E-03 | 0.7398 | 0.9935 | Discovery |
| FCGR3B | Inverse variance weighted | 2 | 1.0918 | 1.0692 | 1.1148 | 1.58E-16 | NA | NA | Discovery |
| FCN1 | Weighted median | 6 | 0.9885 | 0.9773 | 0.9998 | 4.71E-02 | 0.0169 | 0.4875 | Discovery |
| FETUB | Inverse variance weighted | 4 | 0.9908 | 0.9849 | 0.9967 | 2.35E-03 | 0.9349 | 0.6619 | Discovery |
| FGFBP1 | Wald ratio | 1 | 0.9309 | 0.8755 | 0.9898 | 2.21E-02 | NA | NA | Discovery |
| FGR | Inverse variance weighted | 3 | 0.9783 | 0.9690 | 0.9877 | 7.20E-06 | NA | NA | Discovery |
| FGR | Weighted median | 3 | 0.9784 | 0.9573 | 0.9999 | 4.90E-02 | NA | NA | Discovery |
| FLRT3 | Inverse variance weighted | 5 | 0.9816 | 0.9712 | 0.9920 | 5.59E-04 | 0.7670 | 0.6669 | Discovery |
| FN1 | Inverse variance weighted | 2 | 0.9565 | 0.9433 | 0.9699 | 3.61E-10 | NA | NA | Discovery |
| G6PD | Wald ratio | 1 | 1.1534 | 1.0926 | 1.2176 | 2.39E-07 | NA | NA | Discovery |
| GAA | Inverse variance weighted | 3 | 0.9841 | 0.9744 | 0.9940 | 1.62E-03 | NA | NA | Discovery |
| GAA | Weighted median | 3 | 0.9876 | 0.9762 | 0.9991 | 3.52E-02 | NA | NA | Discovery |
| GCLM | Inverse variance weighted | 2 | 0.9812 | 0.9780 | 0.9843 | 8.42E-31 | NA | NA | Discovery |
| GGPS1 | Wald ratio | 1 | 1.0701 | 1.0225 | 1.1199 | 3.53E-03 | NA | NA | Discovery |
| GKN2 | Inverse variance weighted | 2 | 0.9857 | 0.9852 | 0.9862 | 0.00E+00 | NA | NA | Discovery |
| GLRX2 | Inverse variance weighted | 2 | 0.9882 | 0.9765 | 1.0000 | 4.97E-02 | NA | NA | Discovery |
| GMPR2 | Wald ratio | 1 | 0.9531 | 0.9355 | 0.9710 | 4.41E-07 | NA | NA | Discovery |
| GNMT | Inverse variance weighted | 2 | 1.0268 | 1.0070 | 1.0470 | 7.83E-03 | NA | NA | Discovery |
| GOLM1 | Inverse variance weighted | 3 | 1.0075 | 1.0061 | 1.0089 | 6.09E-26 | NA | NA | Discovery |
| GP1BA | Wald ratio | 1 | 0.9527 | 0.9253 | 0.9809 | 1.13E-03 | NA | NA | Discovery |
| GPCPD1 | Inverse variance weighted | 2 | 0.9591 | 0.9451 | 0.9734 | 2.80E-08 | NA | NA | Discovery |
| GPNMB | Weighted median | 4 | 1.0220 | 1.0076 | 1.0367 | 2.61E-03 | 0.0002 | 0.1439 | Discovery |
| GSS | Inverse variance weighted | 2 | 1.0345 | 1.0062 | 1.0637 | 1.66E-02 | NA | NA | Discovery |
| GSTO1 | Wald ratio | 1 | 1.0378 | 1.0116 | 1.0646 | 4.44E-03 | NA | NA | Discovery |
| GSTP1 | Inverse variance weighted | 2 | 1.0108 | 1.0077 | 1.0140 | 1.17E-11 | NA | NA | Discovery |
| GZMK | Inverse variance weighted | 3 | 0.9952 | 0.9931 | 0.9974 | 1.43E-05 | NA | NA | Discovery |
| HAGH | Inverse variance weighted | 3 | 1.0219 | 1.0045 | 1.0396 | 1.36E-02 | NA | NA | Discovery |
| HAPLN1 | Inverse variance weighted | 2 | 1.0189 | 1.0188 | 1.0191 | 0.00E+00 | NA | NA | Discovery |
| HAPLN4 | Wald ratio | 1 | 1.0569 | 1.0076 | 1.1087 | 2.33E-02 | NA | NA | Discovery |
| HEXB | Inverse variance weighted | 2 | 1.0083 | 1.0018 | 1.0148 | 1.24E-02 | NA | NA | Discovery |
| HLA-DQA2 | Inverse variance weighted | 3 | 1.0791 | 1.0238 | 1.1374 | 4.55E-03 | NA | NA | Discovery |
| HLA-DQA2 | Weighted median | 3 | 1.0764 | 1.0550 | 1.0982 | 6.51E-13 | NA | NA | Discovery |
| HPGDS | Wald ratio | 1 | 1.0314 | 1.0075 | 1.0559 | 9.71E-03 | NA | NA | Discovery |
| HS6ST1 | Weighted median | 3 | 1.0318 | 1.0057 | 1.0586 | 1.66E-02 | NA | NA | Discovery |
| HSPB1 | Wald ratio | 1 | 1.0214 | 1.0016 | 1.0417 | 3.42E-02 | NA | NA | Discovery |
| HTN3 | Inverse variance weighted | 2 | 1.0378 | 1.0351 | 1.0404 | 1.54E-173 | NA | NA | Discovery |
| HTRA1 | Inverse variance weighted | 2 | 0.9472 | 0.9435 | 0.9509 | 5.05E-163 | NA | NA | Discovery |
| HYAL1 | Wald ratio | 1 | 0.9549 | 0.9235 | 0.9875 | 6.97E-03 | NA | NA | Discovery |
| IDI2 | Inverse variance weighted | 2 | 0.9906 | 0.9894 | 0.9918 | 7.86E-51 | NA | NA | Discovery |
| IDS | Wald ratio | 1 | 0.9492 | 0.9017 | 0.9992 | 4.67E-02 | NA | NA | Discovery |
| IFNAR1 | Inverse variance weighted | 2 | 0.9477 | 0.9464 | 0.9490 | 0.00E+00 | NA | NA | Discovery |
| IGF1R | Inverse variance weighted | 2 | 0.9702 | 0.9686 | 0.9717 | 5.64E-295 | NA | NA | Discovery |
| IGLL1 | Inverse variance weighted | 4 | 0.9910 | 0.9822 | 0.9998 | 4.61E-02 | 0.5735 | 0.8679 | Discovery |
| IL1RL2 | Inverse variance weighted | 3 | 0.9919 | 0.9874 | 0.9965 | 5.18E-04 | NA | NA | Discovery |
| IL1RN | Inverse variance weighted | 2 | 1.0193 | 1.0133 | 1.0253 | 1.52E-10 | NA | NA | Discovery |
| IL2RA | Wald ratio | 1 | 0.9271 | 0.8670 | 0.9913 | 2.68E-02 | NA | NA | Discovery |
| IL2RB | Inverse variance weighted | 2 | 1.0357 | 1.0334 | 1.0380 | 3.50E-211 | NA | NA | Discovery |
| ISLR2 | Inverse variance weighted | 2 | 1.0237 | 1.0128 | 1.0348 | 1.83E-05 | NA | NA | Discovery |
| ITIH5 | Inverse variance weighted | 7 | 0.9930 | 0.9873 | 0.9988 | 1.72E-02 | 0.7132 | 0.3286 | Discovery |
| ITPKA | Wald ratio | 1 | 1.0980 | 1.0354 | 1.1644 | 1.79E-03 | NA | NA | Discovery |
| JUND | Wald ratio | 1 | 0.9246 | 0.8643 | 0.9892 | 2.29E-02 | NA | NA | Discovery |
| KDELC2 | Inverse variance weighted | 2 | 1.0931 | 1.0842 | 1.1020 | 8.37E-102 | NA | NA | Discovery |
| KIAA1161 | Wald ratio | 1 | 1.0484 | 1.0134 | 1.0847 | 6.43E-03 | NA | NA | Discovery |
| KIR2DL4 | Inverse variance weighted | 2 | 0.9879 | 0.9776 | 0.9983 | 2.31E-02 | NA | NA | Discovery |
| KYNU | Inverse variance weighted | 2 | 0.9818 | 0.9770 | 0.9867 | 5.03E-13 | NA | NA | Discovery |
| LCN1 | Wald ratio | 1 | 0.9369 | 0.8829 | 0.9942 | 3.14E-02 | NA | NA | Discovery |
| LEAP2 | Wald ratio | 1 | 0.9787 | 0.9658 | 0.9918 | 1.50E-03 | NA | NA | Discovery |
| LEFTY2 | Inverse variance weighted | 5 | 0.9850 | 0.9710 | 0.9992 | 3.86E-02 | 0.4878 | 0.5557 | Discovery |
| LGMN | Inverse variance weighted | 3 | 0.9880 | 0.9777 | 0.9984 | 2.34E-02 | NA | NA | Discovery |
| LILRA2 | Inverse variance weighted | 3 | 1.0070 | 1.0018 | 1.0122 | 8.28E-03 | NA | NA | Discovery |
| LILRA5 | Weighted median | 4 | 0.9875 | 0.9760 | 0.9992 | 3.62E-02 | 0.1471 | 0.8251 | Discovery |
| LMAN2L | Wald ratio | 1 | 1.0396 | 1.0002 | 1.0806 | 4.88E-02 | NA | NA | Discovery |
| LRP4 | Wald ratio | 1 | 1.0345 | 1.0178 | 1.0514 | 4.47E-05 | NA | NA | Discovery |
| LRPAP1 | Inverse variance weighted | 3 | 1.0120 | 1.0076 | 1.0165 | 1.02E-07 | NA | NA | Discovery |
| LRRC15 | Inverse variance weighted | 2 | 0.9891 | 0.9791 | 0.9991 | 3.34E-02 | NA | NA | Discovery |
| LY9 | Wald ratio | 1 | 1.0583 | 1.0054 | 1.1139 | 3.02E-02 | NA | NA | Discovery |
| LYVE1 | Inverse variance weighted | 5 | 1.0186 | 1.0014 | 1.0362 | 3.38E-02 | 0.4458 | 0.2927 | Discovery |
| MANEA | Weighted median | 3 | 1.0102 | 1.0003 | 1.0202 | 4.44E-02 | NA | NA | Discovery |
| MANF | Wald ratio | 1 | 1.1116 | 1.0442 | 1.1834 | 9.14E-04 | NA | NA | Discovery |
| MAPK12 | Inverse variance weighted | 2 | 0.9600 | 0.9360 | 0.9846 | 1.56E-03 | NA | NA | Discovery |
| MAPKAPK2 | Inverse variance weighted | 2 | 1.0184 | 1.0066 | 1.0304 | 2.25E-03 | NA | NA | Discovery |
| MATN2 | Inverse variance weighted | 4 | 0.9888 | 0.9814 | 0.9962 | 3.11E-03 | 0.9502 | 0.7084 | Discovery |
| MAX | Wald ratio | 1 | 1.1156 | 1.0904 | 1.1414 | 6.32E-21 | NA | NA | Discovery |
| MFAP2 | Weighted median | 3 | 1.0119 | 1.0003 | 1.0237 | 4.43E-02 | NA | NA | Discovery |
| MGAT2 | Wald ratio | 1 | 1.0310 | 1.0001 | 1.0629 | 4.96E-02 | NA | NA | Discovery |
| MINPP1 | Wald ratio | 1 | 1.0506 | 1.0097 | 1.0932 | 1.49E-02 | NA | NA | Discovery |
| MMP19 | Inverse variance weighted | 2 | 0.9572 | 0.9199 | 0.9959 | 3.06E-02 | NA | NA | Discovery |
| MSMB | Inverse variance weighted | 3 | 0.9939 | 0.9905 | 0.9973 | 4.23E-04 | NA | NA | Discovery |
| MSR1 | Inverse variance weighted | 3 | 0.9917 | 0.9902 | 0.9932 | 4.50E-28 | NA | NA | Discovery |
| MST1 | Inverse variance weighted | 2 | 1.0118 | 1.0095 | 1.0141 | 4.79E-24 | NA | NA | Discovery |
| MTHFD2 | Wald ratio | 1 | 1.1082 | 1.0364 | 1.1849 | 2.63E-03 | NA | NA | Discovery |
| MTHFSD | Inverse variance weighted | 2 | 0.9950 | 0.9923 | 0.9976 | 1.99E-04 | NA | NA | Discovery |
| MUC16 | Wald ratio | 1 | 0.9636 | 0.9402 | 0.9875 | 3.00E-03 | NA | NA | Discovery |
| NADK | Inverse variance weighted | 2 | 0.9826 | 0.9687 | 0.9966 | 1.52E-02 | NA | NA | Discovery |
| NAGK | Inverse variance weighted | 3 | 0.9842 | 0.9783 | 0.9901 | 2.06E-07 | NA | NA | Discovery |
| NCAM2 | Inverse variance weighted | 8 | 0.9833 | 0.9757 | 0.9910 | 2.21E-05 | 0.8180 | 0.9268 | Discovery |
| NCAM2 | Weighted median | 8 | 0.9858 | 0.9732 | 0.9986 | 2.97E-02 | 0.8180 | 0.9268 | Discovery |
| NCF1 | Inverse variance weighted | 2 | 1.0115 | 1.0086 | 1.0144 | 5.92E-15 | NA | NA | Discovery |
| NCR3 | Inverse variance weighted | 3 | 0.9333 | 0.8822 | 0.9875 | 1.65E-02 | NA | NA | Discovery |
| NCR3 | Weighted median | 3 | 0.9426 | 0.8991 | 0.9882 | 1.42E-02 | NA | NA | Discovery |
| NDE1 | Wald ratio | 1 | 0.8996 | 0.8484 | 0.9538 | 3.94E-04 | NA | NA | Discovery |
| NDRG3 | Wald ratio | 1 | 1.2257 | 1.1434 | 1.3140 | 9.76E-09 | NA | NA | Discovery |
| NID1 | Inverse variance weighted | 2 | 0.9870 | 0.9795 | 0.9946 | 7.84E-04 | NA | NA | Discovery |
| NMB | Inverse variance weighted | 2 | 1.0520 | 1.0435 | 1.0607 | 3.14E-34 | NA | NA | Discovery |
| NME2 | Inverse variance weighted | 2 | 0.9902 | 0.9866 | 0.9938 | 1.39E-07 | NA | NA | Discovery |
| NOTCH1 | Inverse variance weighted | 2 | 0.9602 | 0.9433 | 0.9773 | 6.83E-06 | NA | NA | Discovery |
| NPPA | Wald ratio | 1 | 0.8563 | 0.8007 | 0.9157 | 5.86E-06 | NA | NA | Discovery |
| NPPB | Inverse variance weighted | 3 | 0.9712 | 0.9515 | 0.9914 | 5.30E-03 | NA | NA | Discovery |
| NPPB | Weighted median | 3 | 0.9775 | 0.9571 | 0.9983 | 3.38E-02 | NA | NA | Discovery |
| NQO1 | Wald ratio | 1 | 1.0156 | 1.0011 | 1.0304 | 3.54E-02 | NA | NA | Discovery |
| NQO2 | Inverse variance weighted | 2 | 1.0065 | 1.0058 | 1.0071 | 5.62E-84 | NA | NA | Discovery |
| NRP1 | Inverse variance weighted | 4 | 1.0080 | 1.0009 | 1.0151 | 2.68E-02 | 0.6860 | 0.4970 | Discovery |
| NT5E | Inverse variance weighted | 3 | 1.0010 | 1.0002 | 1.0018 | 1.60E-02 | NA | NA | Discovery |
| NUDT12 | Wald ratio | 1 | 0.9725 | 0.9506 | 0.9949 | 1.65E-02 | NA | NA | Discovery |
| NUDT5 | Wald ratio | 1 | 1.1262 | 1.0649 | 1.1910 | 3.12E-05 | NA | NA | Discovery |
| OBP2B | Inverse variance weighted | 3 | 1.0181 | 1.0099 | 1.0263 | 1.33E-05 | NA | NA | Discovery |
| OBP2B | Weighted median | 3 | 1.0170 | 1.0048 | 1.0293 | 6.17E-03 | NA | NA | Discovery |
| PAM | Inverse variance weighted | 2 | 1.0059 | 1.0047 | 1.0071 | 2.16E-22 | NA | NA | Discovery |
| PCBD1 | Inverse variance weighted | 3 | 1.0148 | 1.0071 | 1.0226 | 1.65E-04 | NA | NA | Discovery |
| PCSK7 | Inverse variance weighted | 2 | 0.9888 | 0.9882 | 0.9893 | 0.00E+00 | NA | NA | Discovery |
| PENK | Inverse variance weighted | 3 | 1.0254 | 1.0078 | 1.0434 | 4.64E-03 | NA | NA | Discovery |
| PENK | Weighted median | 3 | 1.0262 | 1.0084 | 1.0443 | 3.74E-03 | NA | NA | Discovery |
| PLAUR | Inverse variance weighted | 2 | 1.0129 | 1.0129 | 1.0129 | 0.00E+00 | NA | NA | Discovery |
| PLEK | Inverse variance weighted | 3 | 1.0292 | 1.0137 | 1.0449 | 2.04E-04 | NA | NA | Discovery |
| PNP | Inverse variance weighted | 2 | 1.0288 | 1.0253 | 1.0323 | 6.47E-61 | NA | NA | Discovery |
| POLI | Wald ratio | 1 | 0.8512 | 0.8133 | 0.8908 | 4.13E-12 | NA | NA | Discovery |
| POR | Inverse variance weighted | 2 | 1.0324 | 1.0244 | 1.0405 | 1.21E-15 | NA | NA | Discovery |
| PPA1 | Inverse variance weighted | 2 | 1.0197 | 1.0046 | 1.0351 | 1.06E-02 | NA | NA | Discovery |
| PPID | Inverse variance weighted | 2 | 1.0150 | 1.0147 | 1.0152 | 0.00E+00 | NA | NA | Discovery |
| PPT1 | Inverse variance weighted | 2 | 0.9627 | 0.9494 | 0.9762 | 8.41E-08 | NA | NA | Discovery |
| PRCP | Inverse variance weighted | 2 | 1.0057 | 1.0026 | 1.0087 | 2.29E-04 | NA | NA | Discovery |
| PRDX1 | Weighted median | 3 | 1.0397 | 1.0044 | 1.0762 | 2.70E-02 | NA | NA | Discovery |
| PRSS27 | Inverse variance weighted | 2 | 0.9824 | 0.9669 | 0.9980 | 2.77E-02 | NA | NA | Discovery |
| PRSS3 | Wald ratio | 1 | 0.9588 | 0.9459 | 0.9720 | 1.58E-09 | NA | NA | Discovery |
| PRSS57 | Wald ratio | 1 | 0.9280 | 0.8663 | 0.9942 | 3.36E-02 | NA | NA | Discovery |
| PSG3 | Inverse variance weighted | 2 | 1.0245 | 1.0155 | 1.0337 | 9.37E-08 | NA | NA | Discovery |
| PTGFRN | Inverse variance weighted | 3 | 1.0011 | 1.0002 | 1.0019 | 1.55E-02 | NA | NA | Discovery |
| PTHLH | Inverse variance weighted | 2 | 1.0244 | 1.0069 | 1.0421 | 5.96E-03 | NA | NA | Discovery |
| PVRL4 | Wald ratio | 1 | 1.0874 | 1.0050 | 1.1766 | 3.72E-02 | NA | NA | Discovery |
| PYGL | Inverse variance weighted | 3 | 1.0279 | 1.0137 | 1.0422 | 1.01E-04 | NA | NA | Discovery |
| PYGL | Weighted median | 3 | 1.0314 | 1.0034 | 1.0602 | 2.78E-02 | NA | NA | Discovery |
| RAD23B | Wald ratio | 1 | 0.9123 | 0.8438 | 0.9864 | 2.12E-02 | NA | NA | Discovery |
| RARRES2 | Wald ratio | 1 | 1.0318 | 1.0135 | 1.0503 | 5.88E-04 | NA | NA | Discovery |
| RBP7 | Inverse variance weighted | 2 | 0.9686 | 0.9557 | 0.9817 | 3.08E-06 | NA | NA | Discovery |
| RCN1 | Wald ratio | 1 | 1.0337 | 1.0022 | 1.0663 | 3.58E-02 | NA | NA | Discovery |
| REG3G | Inverse variance weighted | 2 | 1.0063 | 1.0033 | 1.0094 | 4.67E-05 | NA | NA | Discovery |
| RGS18 | Wald ratio | 1 | 0.9360 | 0.8766 | 0.9993 | 4.77E-02 | NA | NA | Discovery |
| RPE | Wald ratio | 1 | 1.0678 | 1.0163 | 1.1220 | 9.33E-03 | NA | NA | Discovery |
| RRM1 | Wald ratio | 1 | 1.1004 | 1.0300 | 1.1756 | 4.55E-03 | NA | NA | Discovery |
| RSPO3 | Wald ratio | 1 | 1.0387 | 1.0031 | 1.0755 | 3.28E-02 | NA | NA | Discovery |
| RSPO4 | Inverse variance weighted | 4 | 0.9916 | 0.9832 | 1.0000 | 5.00E-02 | 0.9448 | 0.8767 | Discovery |
| S100A2 | Inverse variance weighted | 2 | 1.0257 | 1.0207 | 1.0308 | 5.34E-24 | NA | NA | Discovery |
| SAA1 | Inverse variance weighted | 3 | 0.9864 | 0.9809 | 0.9920 | 2.03E-06 | NA | NA | Discovery |
| SAA1 | Weighted median | 3 | 0.9865 | 0.9756 | 0.9975 | 1.66E-02 | NA | NA | Discovery |
| SAA2 | Inverse variance weighted | 6 | 0.9881 | 0.9817 | 0.9946 | 3.22E-04 | 0.7054 | 0.9521 | Discovery |
| SCGB3A1 | Inverse variance weighted | 2 | 0.9781 | 0.9660 | 0.9903 | 4.48E-04 | NA | NA | Discovery |
| SCO2 | Wald ratio | 1 | 0.9226 | 0.8840 | 0.9629 | 2.22E-04 | NA | NA | Discovery |
| SCUBE3 | Inverse variance weighted | 2 | 1.0047 | 1.0025 | 1.0068 | 2.27E-05 | NA | NA | Discovery |
| SECTM1 | Wald ratio | 1 | 1.0245 | 1.0023 | 1.0472 | 3.07E-02 | NA | NA | Discovery |
| SELL | Inverse variance weighted | 2 | 1.0099 | 1.0046 | 1.0153 | 2.35E-04 | NA | NA | Discovery |
| SELP | Inverse variance weighted | 3 | 1.0157 | 1.0113 | 1.0202 | 3.89E-12 | NA | NA | Discovery |
| SELP | Weighted median | 3 | 1.0166 | 1.0015 | 1.0318 | 3.07E-02 | NA | NA | Discovery |
| SEMA5A | Inverse variance weighted | 2 | 1.0121 | 1.0022 | 1.0222 | 1.67E-02 | NA | NA | Discovery |
| SERPINA1 | Inverse variance weighted | 2 | 0.9945 | 0.9902 | 0.9987 | 1.07E-02 | NA | NA | Discovery |
| SERPINA11 | Inverse variance weighted | 4 | 1.0125 | 1.0043 | 1.0209 | 2.82E-03 | 0.6529 | 0.3809 | Discovery |
| SERPINA11 | Weighted median | 4 | 1.0127 | 1.0001 | 1.0255 | 4.76E-02 | 0.6529 | 0.3809 | Discovery |
| SERPINA12 | Inverse variance weighted | 2 | 0.9934 | 0.9896 | 0.9971 | 5.78E-04 | NA | NA | Discovery |
| SERPINA4 | Inverse variance weighted | 3 | 0.9902 | 0.9823 | 0.9981 | 1.51E-02 | NA | NA | Discovery |
| SERPINA5 | Inverse variance weighted | 3 | 1.0126 | 1.0043 | 1.0210 | 2.95E-03 | NA | NA | Discovery |
| SERPINB13 | Wald ratio | 1 | 0.9699 | 0.9457 | 0.9948 | 1.81E-02 | NA | NA | Discovery |
| SERPINF2 | Inverse variance weighted | 5 | 1.0198 | 1.0093 | 1.0304 | 2.11E-04 | 0.7303 | 0.2662 | Discovery |
| SERPINF2 | Weighted median | 5 | 1.0243 | 1.0058 | 1.0432 | 9.81E-03 | 0.7303 | 0.2662 | Discovery |
| SF3B4 | Wald ratio | 1 | 1.1315 | 1.0360 | 1.2358 | 6.01E-03 | NA | NA | Discovery |
| SFTPD | Inverse variance weighted | 4 | 1.0096 | 1.0062 | 1.0129 | 1.64E-08 | 0.9900 | 0.8130 | Discovery |
| SHANK3 | Wald ratio | 1 | 0.9689 | 0.9417 | 0.9968 | 2.92E-02 | NA | NA | Discovery |
| SHMT1 | Inverse variance weighted | 2 | 0.9782 | 0.9665 | 0.9901 | 3.55E-04 | NA | NA | Discovery |
| SIGLEC1 | Inverse variance weighted | 2 | 0.9871 | 0.9849 | 0.9892 | 7.55E-32 | NA | NA | Discovery |
| SIGLEC14 | Weighted median | 10 | 0.9882 | 0.9770 | 0.9996 | 4.26E-02 | 0.0208 | 0.4424 | Discovery |
| SLAMF6 | Wald ratio | 1 | 1.0349 | 1.0112 | 1.0591 | 3.75E-03 | NA | NA | Discovery |
| SMPDL3A | Inverse variance weighted | 2 | 1.0202 | 1.0094 | 1.0311 | 2.38E-04 | NA | NA | Discovery |
| ST3GAL1 | Inverse variance weighted | 3 | 1.0108 | 1.0021 | 1.0197 | 1.49E-02 | NA | NA | Discovery |
| STAT3 | Wald ratio | 1 | 1.0291 | 1.0012 | 1.0578 | 4.08E-02 | NA | NA | Discovery |
| STAT6 | Wald ratio | 1 | 0.9022 | 0.8581 | 0.9486 | 5.77E-05 | NA | NA | Discovery |
| STIM1 | Wald ratio | 1 | 0.9520 | 0.9118 | 0.9939 | 2.54E-02 | NA | NA | Discovery |
| SULT1A3 | Inverse variance weighted | 7 | 0.9779 | 0.9649 | 0.9910 | 1.02E-03 | 0.7201 | 0.6128 | Discovery |
| TAPBP | Inverse variance weighted | 2 | 0.9866 | 0.9810 | 0.9923 | 4.38E-06 | NA | NA | Discovery |
| TCEA2 | Wald ratio | 1 | 1.0529 | 1.0221 | 1.0847 | 6.74E-04 | NA | NA | Discovery |
| TCL1A | Inverse variance weighted | 2 | 0.9378 | 0.8854 | 0.9932 | 2.83E-02 | NA | NA | Discovery |
| TEC | Inverse variance weighted | 2 | 1.0262 | 1.0122 | 1.0403 | 2.21E-04 | NA | NA | Discovery |
| TES | Wald ratio | 1 | 1.0679 | 1.0086 | 1.1306 | 2.42E-02 | NA | NA | Discovery |
| TESC | Inverse variance weighted | 2 | 0.9462 | 0.9130 | 0.9806 | 2.38E-03 | NA | NA | Discovery |
| TFF2 | Inverse variance weighted | 3 | 0.9601 | 0.9226 | 0.9991 | 4.49E-02 | NA | NA | Discovery |
| TGM4 | Wald ratio | 1 | 1.1229 | 1.0374 | 1.2155 | 4.13E-03 | NA | NA | Discovery |
| TLR1 | Wald ratio | 1 | 1.0573 | 1.0220 | 1.0937 | 1.28E-03 | NA | NA | Discovery |
| TMEM106A | Wald ratio | 1 | 1.2897 | 1.2039 | 1.3815 | 4.20E-13 | NA | NA | Discovery |
| TMEM132B | Inverse variance weighted | 2 | 1.0043 | 1.0015 | 1.0071 | 3.05E-03 | NA | NA | Discovery |
| TMEM132C | Inverse variance weighted | 3 | 1.0106 | 1.0003 | 1.0211 | 4.37E-02 | NA | NA | Discovery |
| TMEM132D | Inverse variance weighted | 4 | 0.9593 | 0.9500 | 0.9687 | 6.25E-17 | 0.9218 | 0.8558 | Discovery |
| TMEM132D | Weighted median | 4 | 0.9573 | 0.9317 | 0.9836 | 1.58E-03 | 0.9218 | 0.8558 | Discovery |
| TMEM9 | Wald ratio | 1 | 1.1123 | 1.0495 | 1.1787 | 3.27E-04 | NA | NA | Discovery |
| TNFRSF17 | Wald ratio | 1 | 0.9658 | 0.9352 | 0.9974 | 3.44E-02 | NA | NA | Discovery |
| TNFRSF1A | Inverse variance weighted | 2 | 0.9779 | 0.9732 | 0.9826 | 9.78E-20 | NA | NA | Discovery |
| TNFRSF1B | Inverse variance weighted | 3 | 0.9915 | 0.9842 | 0.9989 | 2.44E-02 | NA | NA | Discovery |
| TNXB | Inverse variance weighted | 4 | 1.0161 | 1.0067 | 1.0257 | 7.55E-04 | 0.4643 | 0.3434 | Discovery |
| TNXB | Weighted median | 4 | 1.0152 | 1.0038 | 1.0267 | 8.81E-03 | 0.4643 | 0.3434 | Discovery |
| TTL | Wald ratio | 1 | 0.9188 | 0.8512 | 0.9918 | 2.99E-02 | NA | NA | Discovery |
| TYRO3 | Wald ratio | 1 | 1.1567 | 1.1118 | 1.2034 | 5.70E-13 | NA | NA | Discovery |
| UBE2F | Wald ratio | 1 | 1.0726 | 1.0128 | 1.1359 | 1.66E-02 | NA | NA | Discovery |
| UCMA | Inverse variance weighted | 9 | 1.0084 | 1.0034 | 1.0136 | 1.11E-03 | 0.8830 | 0.4272 | Discovery |
| UCMA | Weighted median | 9 | 1.0103 | 1.0008 | 1.0199 | 3.39E-02 | 0.8830 | 0.4272 | Discovery |
| UGDH | Inverse variance weighted | 2 | 1.0098 | 1.0080 | 1.0116 | 1.47E-26 | NA | NA | Discovery |
| UGT1A6 | Inverse variance weighted | 2 | 1.0123 | 1.0020 | 1.0227 | 1.94E-02 | NA | NA | Discovery |
| ULK3 | Wald ratio | 1 | 1.0706 | 1.0199 | 1.1239 | 5.84E-03 | NA | NA | Discovery |
| UROD | Wald ratio | 1 | 1.0173 | 1.0005 | 1.0344 | 4.30E-02 | NA | NA | Discovery |
| UROS | Inverse variance weighted | 3 | 1.0216 | 1.0026 | 1.0410 | 2.60E-02 | NA | NA | Discovery |
| UROS | Weighted median | 3 | 1.0185 | 1.0021 | 1.0352 | 2.65E-02 | NA | NA | Discovery |
| USP8 | Wald ratio | 1 | 1.1484 | 1.0901 | 1.2099 | 1.94E-07 | NA | NA | Discovery |
| UST | Inverse variance weighted | 4 | 0.9884 | 0.9784 | 0.9985 | 2.42E-02 | 0.9235 | 0.9388 | Discovery |
| VNN2 | Inverse variance weighted | 2 | 0.9880 | 0.9861 | 0.9899 | 8.90E-35 | NA | NA | Discovery |
| VOPP1 | Inverse variance weighted | 2 | 0.9874 | 0.9778 | 0.9971 | 1.10E-02 | NA | NA | Discovery |
| VWA1 | Inverse variance weighted | 2 | 0.9774 | 0.9692 | 0.9857 | 1.05E-07 | NA | NA | Discovery |
| VWC2 | Inverse variance weighted | 3 | 1.0115 | 1.0068 | 1.0163 | 1.48E-06 | NA | NA | Discovery |
| ACHE | Wald ratio | 1 | 1.0163 | 1.0029 | 1.0298 | 1.70E-02 | NA | NA | Validation |
| ADM | Wald ratio | 1 | 0.9807 | 0.9619 | 0.9998 | 4.78E-02 | NA | NA | Validation |
| AMY1A | Wald ratio | 1 | 0.9901 | 0.9807 | 0.9997 | 4.25E-02 | NA | NA | Validation |
| ANGPTL1 | Wald ratio | 1 | 0.9846 | 0.9752 | 0.9940 | 1.45E-03 | NA | NA | Validation |
| APOA5 | Wald ratio | 1 | 0.9519 | 0.9315 | 0.9727 | 7.66E-06 | NA | NA | Validation |
| APOH | Wald ratio | 1 | 1.0236 | 1.0076 | 1.0399 | 3.65E-03 | NA | NA | Validation |
| ARHGAP1 | Wald ratio | 1 | 0.9741 | 0.9510 | 0.9978 | 3.22E-02 | NA | NA | Validation |
| ASAH2;ASAH2B | Wald ratio | 1 | 1.0093 | 1.0019 | 1.0168 | 1.36E-02 | NA | NA | Validation |
| AZU1 | Wald ratio | 1 | 0.9798 | 0.9603 | 0.9997 | 4.68E-02 | NA | NA | Validation |
| C1QL1 | Wald ratio | 1 | 1.0160 | 1.0017 | 1.0305 | 2.79E-02 | NA | NA | Validation |
| CA1 | Wald ratio | 1 | 0.9649 | 0.9391 | 0.9914 | 9.77E-03 | NA | NA | Validation |
| CA3 | Wald ratio | 1 | 1.0254 | 1.0039 | 1.0473 | 2.04E-02 | NA | NA | Validation |
| CCL4 | Wald ratio | 1 | 1.0160 | 1.0039 | 1.0282 | 9.52E-03 | NA | NA | Validation |
| CD59 | Wald ratio | 1 | 0.9866 | 0.9743 | 0.9991 | 3.60E-02 | NA | NA | Validation |
| CDNF | Wald ratio | 1 | 1.0194 | 1.0010 | 1.0381 | 3.91E-02 | NA | NA | Validation |
| CREB3L4 | Wald ratio | 1 | 1.0196 | 1.0040 | 1.0354 | 1.34E-02 | NA | NA | Validation |
| CRISPLD2 | Wald ratio | 1 | 0.9665 | 0.9490 | 0.9843 | 2.60E-04 | NA | NA | Validation |
| CROT | Wald ratio | 1 | 1.0242 | 1.0022 | 1.0467 | 3.11E-02 | NA | NA | Validation |
| CRYZ | Wald ratio | 1 | 0.9952 | 0.9905 | 1.0000 | 4.95E-02 | NA | NA | Validation |
| CTSS | Wald ratio | 1 | 1.0131 | 1.0047 | 1.0216 | 2.19E-03 | NA | NA | Validation |
| ERLEC1 | Wald ratio | 1 | 0.9549 | 0.9334 | 0.9768 | 6.85E-05 | NA | NA | Validation |
| FCGR3B | Wald ratio | 1 | 0.9901 | 0.9804 | 0.9998 | 4.57E-02 | NA | NA | Validation |
| FCN1 | Wald ratio | 1 | 0.9911 | 0.9836 | 0.9988 | 2.29E-02 | NA | NA | Validation |
| FN1 | Wald ratio | 1 | 0.9894 | 0.9832 | 0.9956 | 8.57E-04 | NA | NA | Validation |
| GAA | Wald ratio | 1 | 0.9877 | 0.9769 | 0.9986 | 2.68E-02 | NA | NA | Validation |
| GDI2 | Wald ratio | 1 | 0.9418 | 0.9218 | 0.9622 | 4.19E-08 | NA | NA | Validation |
| GNLY | Wald ratio | 1 | 1.0090 | 1.0030 | 1.0151 | 3.43E-03 | NA | NA | Validation |
| GP1BA | Wald ratio | 1 | 0.9637 | 0.9425 | 0.9854 | 1.13E-03 | NA | NA | Validation |
| GSTO1 | Wald ratio | 1 | 0.9903 | 0.9859 | 0.9948 | 2.12E-05 | NA | NA | Validation |
| HIBCH | Wald ratio | 1 | 1.0097 | 1.0033 | 1.0161 | 2.78E-03 | NA | NA | Validation |
| HPGDS | Wald ratio | 1 | 1.0154 | 1.0061 | 1.0248 | 1.12E-03 | NA | NA | Validation |
| HS6ST1 | Wald ratio | 1 | 1.0317 | 1.0131 | 1.0507 | 7.94E-04 | NA | NA | Validation |
| HSPB1 | Wald ratio | 1 | 0.9856 | 0.9741 | 0.9973 | 1.62E-02 | NA | NA | Validation |
| IDUA | Wald ratio | 1 | 0.9911 | 0.9845 | 0.9978 | 9.47E-03 | NA | NA | Validation |
| IFNAR1 | Wald ratio | 1 | 0.9755 | 0.9564 | 0.9951 | 1.43E-02 | NA | NA | Validation |
| IL11RA | Wald ratio | 1 | 1.0197 | 1.0038 | 1.0358 | 1.51E-02 | NA | NA | Validation |
| IL7R | Wald ratio | 1 | 0.9843 | 0.9751 | 0.9936 | 9.70E-04 | NA | NA | Validation |
| ITIH5 | Wald ratio | 1 | 0.9850 | 0.9711 | 0.9991 | 3.67E-02 | NA | NA | Validation |
| KDELC2 | Wald ratio | 1 | 1.0707 | 1.0605 | 1.0809 | 1.48E-44 | NA | NA | Validation |
| KIAA1161 | Wald ratio | 1 | 1.0186 | 1.0052 | 1.0321 | 6.50E-03 | NA | NA | Validation |
| LCT | Wald ratio | 1 | 0.9868 | 0.9808 | 0.9928 | 1.61E-05 | NA | NA | Validation |
| LEAP2 | Wald ratio | 1 | 0.9717 | 0.9547 | 0.9891 | 1.50E-03 | NA | NA | Validation |
| LILRA5 | Wald ratio | 1 | 0.9918 | 0.9842 | 0.9996 | 3.86E-02 | NA | NA | Validation |
| LMAN2L | Wald ratio | 1 | 1.0165 | 1.0002 | 1.0331 | 4.68E-02 | NA | NA | Validation |
| LRP11 | Wald ratio | 1 | 1.0061 | 1.0012 | 1.0110 | 1.43E-02 | NA | NA | Validation |
| LRRC15 | Wald ratio | 1 | 0.9886 | 0.9783 | 0.9989 | 3.07E-02 | NA | NA | Validation |
| LY9 | Wald ratio | 1 | 1.0108 | 1.0008 | 1.0210 | 3.34E-02 | NA | NA | Validation |
| MANF | Wald ratio | 1 | 1.0196 | 1.0052 | 1.0341 | 7.29E-03 | NA | NA | Validation |
| MGAT2 | Wald ratio | 1 | 1.0224 | 1.0019 | 1.0432 | 3.18E-02 | NA | NA | Validation |
| NELL1 | Wald ratio | 1 | 0.9913 | 0.9831 | 0.9994 | 3.64E-02 | NA | NA | Validation |
| NMB | Wald ratio | 1 | 1.0370 | 1.0109 | 1.0639 | 5.24E-03 | NA | NA | Validation |
| NPPB | Wald ratio | 1 | 0.9759 | 0.9614 | 0.9907 | 1.50E-03 | NA | NA | Validation |
| NPW | Wald ratio | 1 | 1.0120 | 1.0009 | 1.0233 | 3.37E-02 | NA | NA | Validation |
| NT5C | Wald ratio | 1 | 0.9682 | 0.9534 | 0.9833 | 4.39E-05 | NA | NA | Validation |
| OBP2B | Wald ratio | 1 | 1.0162 | 1.0025 | 1.0301 | 2.00E-02 | NA | NA | Validation |
| ORM1 | Wald ratio | 1 | 0.9761 | 0.9575 | 0.9950 | 1.34E-02 | NA | NA | Validation |
| PCYOX1 | Wald ratio | 1 | 0.9923 | 0.9853 | 0.9993 | 3.12E-02 | NA | NA | Validation |
| PEAR1 | Wald ratio | 1 | 0.9816 | 0.9642 | 0.9993 | 4.16E-02 | NA | NA | Validation |
| PPP3CA;PPP3R1 | Wald ratio | 1 | 1.0138 | 1.0022 | 1.0254 | 1.93E-02 | NA | NA | Validation |
| PSAPL1 | Wald ratio | 1 | 1.0115 | 1.0021 | 1.0209 | 1.59E-02 | NA | NA | Validation |
| PSMB1 | Wald ratio | 1 | 0.9754 | 0.9659 | 0.9849 | 5.19E-07 | NA | NA | Validation |
| PTN | Wald ratio | 1 | 1.0182 | 1.0000 | 1.0368 | 5.00E-02 | NA | NA | Validation |
| PYY | Wald ratio | 1 | 1.0440 | 1.0163 | 1.0723 | 1.66E-03 | NA | NA | Validation |
| PZP | Wald ratio | 1 | 1.0120 | 1.0033 | 1.0208 | 6.52E-03 | NA | NA | Validation |
| RARRES2 | Wald ratio | 1 | 1.0208 | 1.0088 | 1.0330 | 6.38E-04 | NA | NA | Validation |
| RPN1 | Wald ratio | 1 | 0.9596 | 0.9484 | 0.9708 | 3.81E-12 | NA | NA | Validation |
| SECTM1 | Wald ratio | 1 | 1.0156 | 1.0014 | 1.0300 | 3.07E-02 | NA | NA | Validation |
| SELP | Wald ratio | 1 | 1.0082 | 1.0004 | 1.0161 | 4.02E-02 | NA | NA | Validation |
| SERPINA11 | Wald ratio | 1 | 1.0186 | 1.0003 | 1.0372 | 4.62E-02 | NA | NA | Validation |
| SERPINA4 | Inverse variance weighted | 2 | 1.0016 | 1.0006 | 1.0025 | 1.64E-03 | NA | NA | Validation |
| SERPINF1 | Wald ratio | 1 | 0.9709 | 0.9619 | 0.9800 | 4.83E-10 | NA | NA | Validation |
| SIRPA | Wald ratio | 1 | 1.0038 | 1.0005 | 1.0072 | 2.48E-02 | NA | NA | Validation |
| SWAP70 | Wald ratio | 1 | 0.9790 | 0.9641 | 0.9942 | 6.83E-03 | NA | NA | Validation |
| TCN2 | Wald ratio | 1 | 0.9929 | 0.9875 | 0.9983 | 1.02E-02 | NA | NA | Validation |
| TEPSIN | Wald ratio | 1 | 1.0305 | 1.0088 | 1.0527 | 5.64E-03 | NA | NA | Validation |
| TGFB1 | Wald ratio | 1 | 1.0229 | 1.0070 | 1.0390 | 4.51E-03 | NA | NA | Validation |
| TLR4;LY96 | Wald ratio | 1 | 1.0110 | 1.0018 | 1.0202 | 1.85E-02 | NA | NA | Validation |
| TNFRSF6B | Wald ratio | 1 | 1.1120 | 1.0854 | 1.1392 | 8.69E-18 | NA | NA | Validation |
| TYMP | Wald ratio | 1 | 0.8858 | 0.8651 | 0.9070 | 9.30E-24 | NA | NA | Validation |
| TYRO3 | Wald ratio | 1 | 1.1149 | 1.0852 | 1.1454 | 2.88E-15 | NA | NA | Validation |

**Notes:** “Proteins” column refers to the name of a protein; “Methods” column refers to the statistical method used to calculated the causal effect; “NSNP” column refers to the number of single nucleotide polymorphism used in causal estimates; “OR” column refers to the odds ratio; “Lower of 95%CI” column refers to the lower limit of 95% confidence interval; “Upper of 95%CI” refers to the upper limit of 95% confidence interval; “P” column refers to the p-value of odds ratio; “P_het_” column refers to the p-value of heterogeneity test; “P_pleio_” column refers to the p-value of horizontal pleiotropy test; “Stage” column refers to the “Discovery” or “Validation” stage.

**Supplementary Table 6: The results of enrichment analyses using circulating proteins closely associated with leukocyte telomere length.**

| **Gene Set** | **Description** | **Size** | **Expect** | **Ratio** | **P** | **Database** |
| --- | --- | --- | --- | --- | --- | --- |
| R-HSA-1566977 | Fibronectin matrix formation | 6 | 0.0056 | 177.6500 | 5.62E-03 | Reactome |
| R-HSA-76009 | Platelet Aggregation (Plug Formation) | 39 | 0.0366 | 54.6630 | 5.94E-04 | Reactome |
| R-HSA-114608 | Platelet degranulation | 129 | 0.1210 | 33.0520 | 4.99E-06 | Reactome |
| R-HSA-76005 | Response to elevated platelet cytosolic Ca2+ | 134 | 0.1257 | 31.8190 | 5.80E-06 | Reactome |
| R-HSA-76002 | Platelet activation, signaling and aggregation | 262 | 0.2458 | 20.3420 | 2.82E-06 | Reactome |
| R-HSA-8957275 | Post-translational protein phosphorylation | 108 | 0.1013 | 19.7390 | 4.46E-03 | Reactome |
| R-HSA-109582 | Hemostasis | 620 | 0.5817 | 8.5962 | 1.78E-04 | Reactome |
| C0040053 | Thrombosis | 45 | 0.0422 | 71.0620 | 9.11E-06 | DisGeNET |
| C0003504 | Aortic Valve Insufficiency | 30 | 0.0281 | 71.0620 | 3.50E-04 | DisGeNET |
| C0149721 | Left Ventricular Hypertrophy | 56 | 0.0525 | 38.0690 | 1.22E-03 | DisGeNET |
| C0013404 | Dyspnea | 70 | 0.0657 | 30.4550 | 1.90E-03 | DisGeNET |
| C1956346 | Coronary Artery Disease | 78 | 0.0732 | 27.3320 | 2.36E-03 | DisGeNET |
| C0018800 | Cardiomegaly | 115 | 0.1079 | 18.5380 | 5.04E-03 | DisGeNET |
| C0020538 | Hypertensive disease | 302 | 0.2833 | 14.1180 | 1.40E-04 | DisGeNET |
| DB01593 | Zinc | 121 | 0.1135 | 17.6190 | 5.57E-03 | DrugBank |
| DB00491 | Miglitol | 5 | 0.0047 | 213.1900 | 4.68E-03 | DrugBank |
| DB00999 | Hydrochlorothiazide | 5 | 0.0047 | 213.1900 | 4.68E-03 | DrugBank |
| DB01325 | Quinethazone | 5 | 0.0047 | 213.1900 | 4.68E-03 | DrugBank |
| DB06779 | Dalteparin | 5 | 0.0047 | 213.1900 | 4.68E-03 | DrugBank |

**Supplementary Table 7: Phenome-wide screening for traits associated with variants located in the target genes.**

| **Proteins** | **SNP** | **CHR** | **BP** | **A1** | **A2** | **EAF** | **N** | **BETA** | **SE** | **P** | **ID** | **Traits** |
| --- | --- | --- | --- | --- | --- | --- | --- | --- | --- | --- | --- | --- |
| SCARF2 | rs5763025 | 22 | 20786488 | C | A | 0.8030 | 321047 | 0.0269 | 0.0030 | 2.31E-19 | ebi-a-GCST007431 | Lung function (FEV1/FVC) |
|  | rs2108746 | 22 | 20788969 | G | A | 0.8030 | 321047 | 0.0268 | 0.0030 | 3.40E-19 | ebi-a-GCST007431 | Lung function (FEV1/FVC) |
|  | rs9610955 | 22 | 20790723 | G | C | 0.8028 | 321047 | 0.0192 | 0.0029 | 6.94E-11 | ebi-a-GCST007432 | FEV1 |
|  | rs5763025 | 22 | 20786488 | C | A | 0.8031 | 321047 | 0.0189 | 0.0029 | 1.13E-10 | ebi-a-GCST007432 | FEV1 |
|  | rs361566 | 22 | 20785639 | A | G | 0.1971 | 321047 | -0.0189 | 0.0029 | 1.17E-10 | ebi-a-GCST007432 | FEV1 |
|  | rs1364904419 | 22 | 20790723 | G | C | 0.8026 | 353315 | -0.0068 | 0.0011 | 8.50E-10 | ieu-b-106 | FEV1/FVC < 0.7 |
|  | rs2108746 | 22 | 20788969 | G | A | 0.8028 | 353315 | -0.0067 | 0.0011 | 1.30E-09 | ieu-b-106 | FEV1/FVC < 0.7 |
|  | rs361566 | 22 | 20785639 | A | G | 0.1974 | 353315 | 0.0066 | 0.0011 | 1.50E-09 | ieu-b-106 | FEV1/FVC < 0.7 |
|  | rs1164026694 | 22 | 20786488 | C | A | 0.8027 | 353315 | -0.0066 | 0.0011 | 1.60E-09 | ieu-b-106 | FEV1/FVC < 0.7 |
|  | rs9619753 | 22 | 20791821 | A | G | 0.2440 | 321047 | 0.0157 | 0.0028 | 1.79E-08 | ebi-a-GCST007431 | Lung function (FEV1/FVC) |
| FN1 | rs13423742 | 2 | 216287796 | G | C | 0.2710 | 321047 | -0.0172 | 0.0028 | 1.08E-09 | ebi-a-GCST007430 | Peak expiratory flow |
|  | rs1250258 | 2 | 216300185 | T | C | 0.7360 | 321047 | 0.0171 | 0.0029 | 2.17E-09 | ebi-a-GCST007430 | Peak expiratory flow |

Notes: SNP = single nucleotide polymorphism; CHR = chromosome; BP = base position from GRCh37 build; A1 = effect allele; A2 = other allele; EAF = effect allele frequency; N = sample size; BETA = effect size; SE = standard error; P = p-value of BETA; ID = identifier for a trait in the open GWAS database (<https://gwas.mrcieu.ac.uk/>).

**Supplementary Figure 1: The comparisons of genetic associations in the PTGFRN locus. A. locus zoom for PTGFRN and IPF. B. locus zoom for PTGFRN and COPD. C. locus zoom for PTGFRN and LTL.** Notes: PTGFRN = Prostaglandin F2 Receptor Inhibitor; IPF = idiopathic pulmonary fibrosis; COPD = chronic obstructive pulmonary disease; LTL = leukocyte telomere length; GWAS = genome-wide association study. Each point represents a single nucleotide polymorphism (SNP) and the r2 is the measurement of linkage disequilibrium. The original p-value was transformed using -log10.


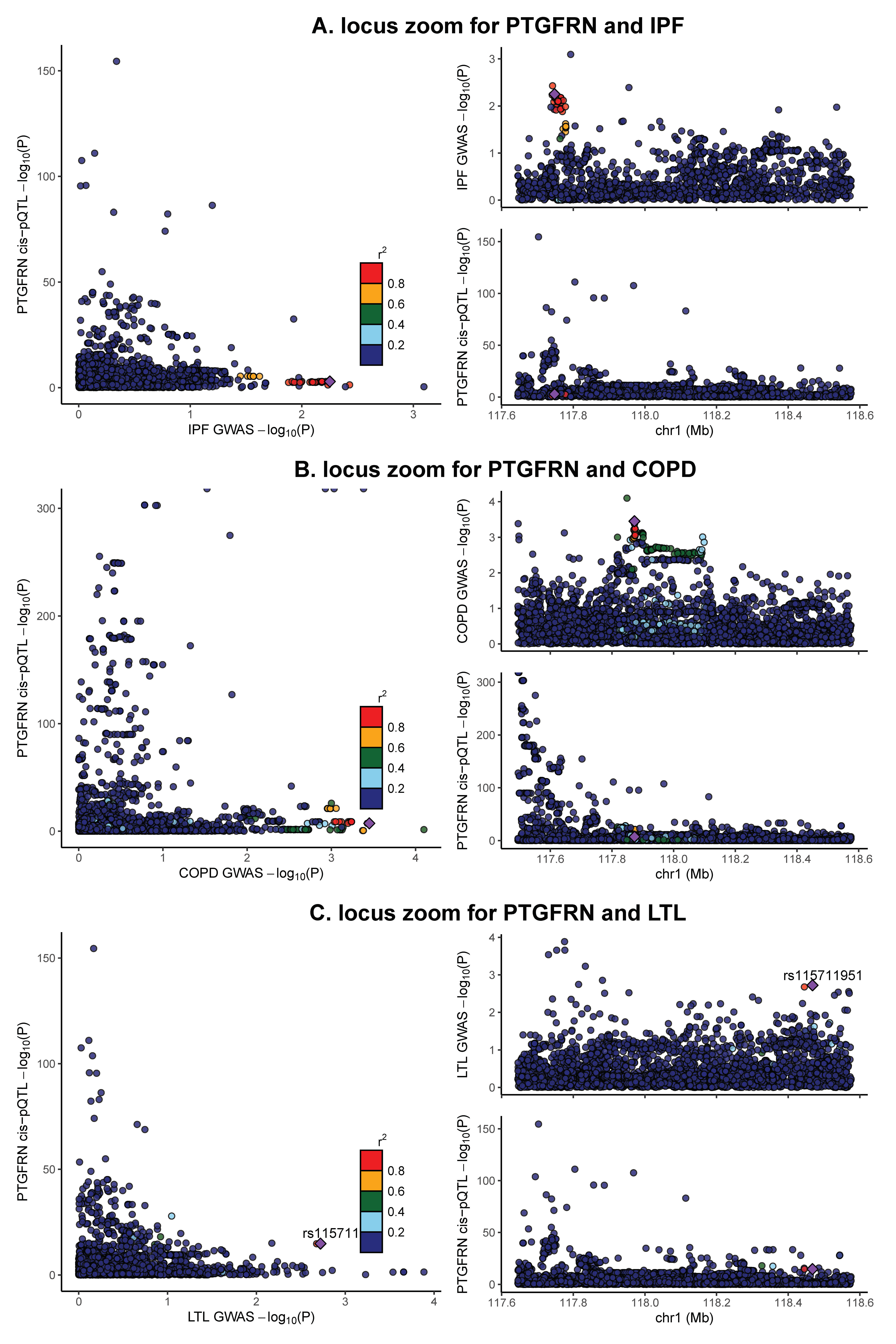


**Supplementary Figure 2: The comparisons of genetic associations in the FN1 locus. A. locus zoom for FN1 and IPF. B. locus zoom for FN1 and COPD. C. locus zoom for FN1 and LTL.** Notes: FN1 = Fibronectin 1; IPF = idiopathic pulmonary fibrosis; COPD = chronic obstructive pulmonary disease; LTL = leukocyte telomere length; GWAS = genome-wide association study. Each point represents a single nucleotide polymorphism (SNP) and the r2 is the measurement of linkage disequilibrium. The original p-value was transformed using -log10.


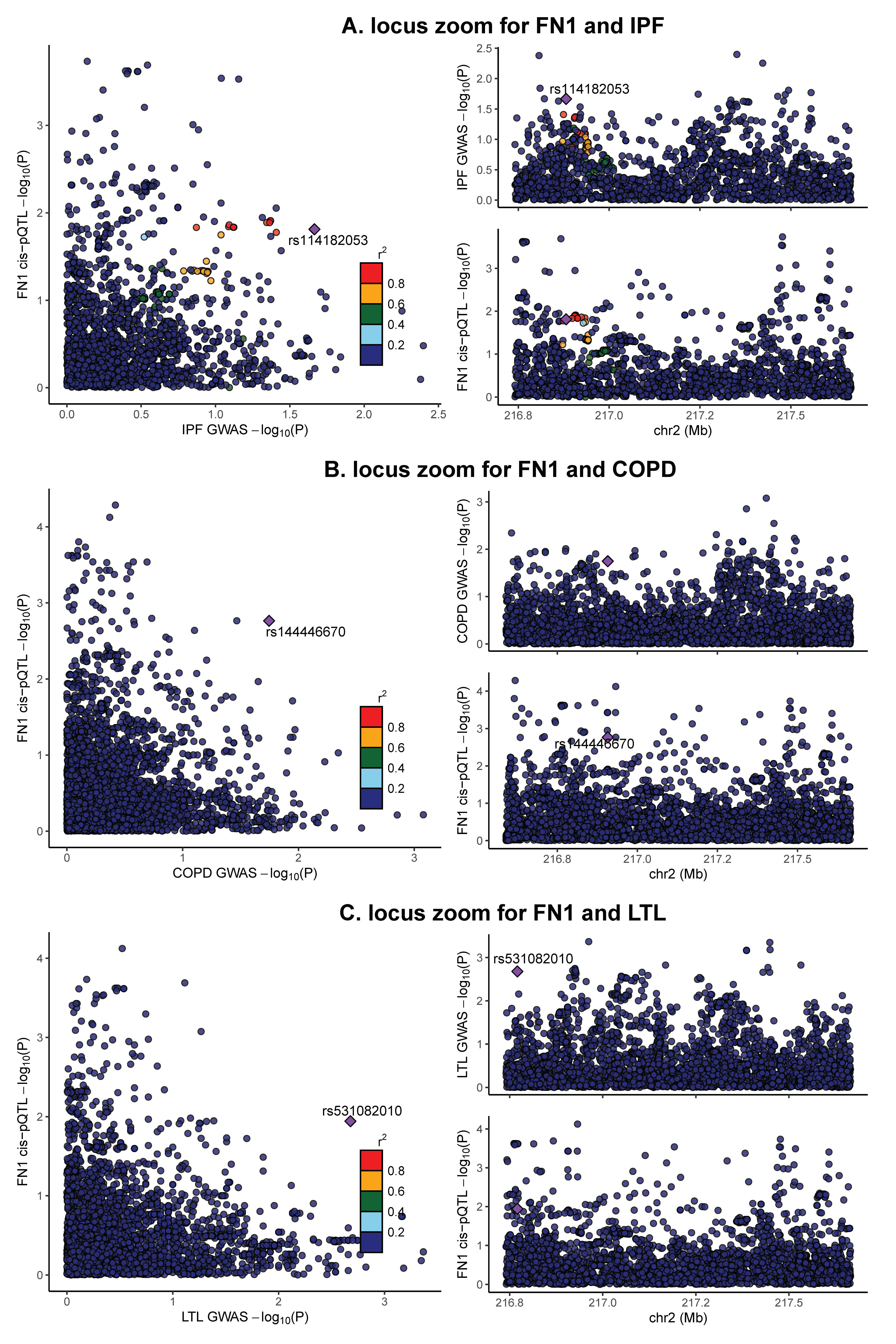


**Supplementary Figure 3: The comparisons of genetic associations in the APOA5 locus. A. locus zoom for APOA5 and IPF. B. locus zoom for APOA5 and COPD. C. locus zoom for APOA5 and LTL.** Notes: APOA5 = Apolipoprotein A5; IPF = idiopathic pulmonary fibrosis; COPD = chronic obstructive pulmonary disease; LTL = leukocyte telomere length; GWAS = genome-wide association study. Each point represents a single nucleotide polymorphism (SNP) and the r2 is the measurement of linkage disequilibrium. The original p-value was transformed using -log10.


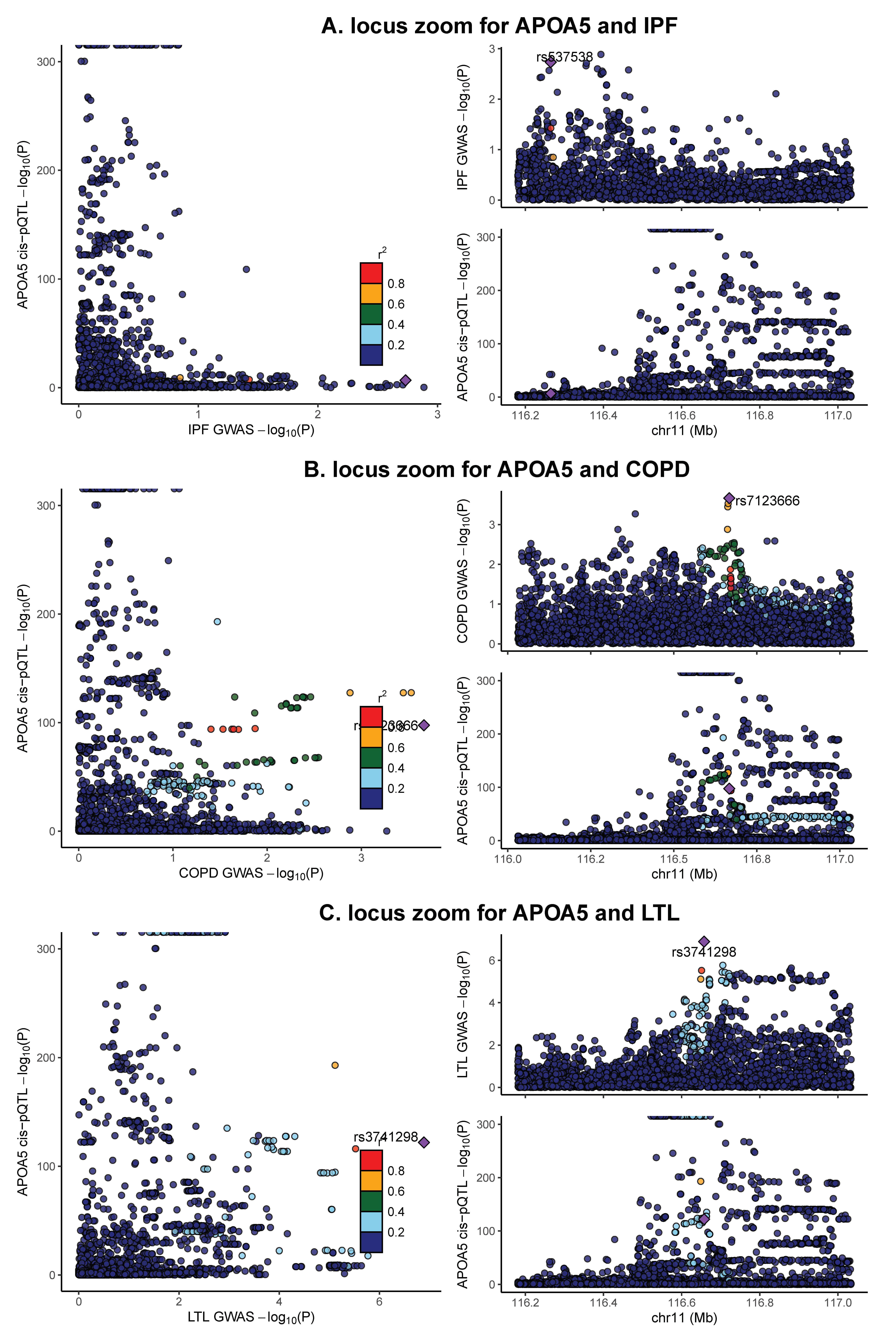

Supplement: Supplementary file 1 — Data S1: [file ACEL-23-e14266-s001.docx]
